# Mining RNAseq data reveals dynamic metaboloepigenetic profiles in human, mouse and bovine pre-implantation embryos

**DOI:** 10.1016/j.isci.2022.103904

**Published:** 2022-02-11

**Authors:** Marcella Pecora Milazzotto, Michael James Noonan, Marcia de Almeida Monteiro Melo Ferraz

**Affiliations:** 1 Center of Natural and Human Sciences, Federal University of ABC, São Paulo, 09210-580 Santo André, Brazil; 2 The Irving K. Barber School of Sciences, The University of British Columbia, Okanagan Campus, Kelowna, BC V1V 1V7, Canada; 3 Gene Center Munich, Ludwig-Maximilians University of Munich, 80539 Munich, Germany; 4 Clinic of Ruminants, Faculty of Veterinary Medicine Ludwig-Maximilians University of Munich, 80539 Munich, Germany

**Keywords:** Biological sciences, Molecular biology, Endocrinology, Omics, Transcriptomics

## Abstract

Metaboloepigenetic regulation has been reported in stem cells, germ cells, and tumor cells. Embryonic metaboloepigenetics, however, have just begun to be described. Here we analyzed RNAseq data to characterize the metaboloepigenetic profiles of human, mouse, and bovine pre-implantation embryos. In embryos, metaboloepigenetic reprogramming was species-specific, varied with the developmental stage and was disrupted with *in vitro* culture. Metabolic pathways and gene expressions were strongly correlated with early embryo DNA methylation and were changed with *in vitro* culture. Although the idea that the *in vitro* environment may influence development is not new, there has been little progress on improving pregnancy rates after decades using *in vitro* fertilization. Hence, the present data will contribute to understanding how the *in vitro* manipulation affects the metaboloepigenetic status of early embryos, which can be used to establish culture strategies aimed at improving the *in vitro* environment and, consequently, pregnancy rates and offspring health.

## Highlights

• Embryonic metaboloepigenetic reprogramming is stage- and species-specific
• *In vitro* culture disrupts the *in vivo* embryonic metaboloepigenetic reprogramming
• Metabolic genes and pathways are highly correlated with embryo methylome

## Graphical abstract

**Figure:**
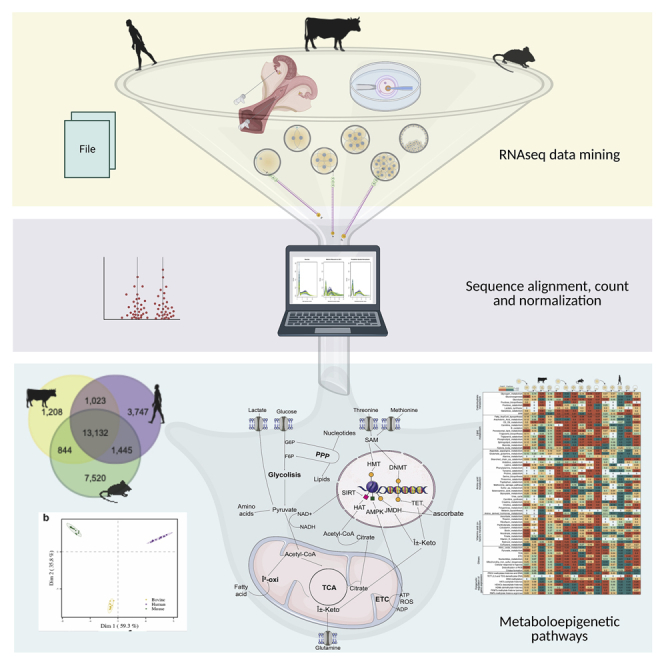

## Introduction

In mammals, epigenetic reprogramming is central to embryonic survival, cell differentiation, and ensuring the proper development of a new organism. Among the different layers of epigenetic control, the methylation of DNA and histone residues are vital to normal cell function and survival. In particular, DNA methylation is involved in the control of transposable elements, X chromosome inactivation, genomic imprinting and cell differentiation (Jansz, 2019; Li and Sasaki, 2011; Sharp et al., 2011). DNA demethylation is also essential for the loss of highly repressive markers on gametes and the establishment of new specific markers, allowing embryonic cells to become totipotent and promoting adequate cell differentiation. Histone methylation also undergoes intense modifications during gametogenesis and early embryogenesis and is crucial for the establishment of totipotency (Ross et al., 2008; Sarmento et al., 2004; Torres-Padilla et al., 2007; Zhang et al., 2012).

During the period over which they undergo epigenetic reprogramming, early embryos are highly sensitive to changes in environmental conditions both *in vivo* (maternal health, diet, medication, etc.) and *in vitro* (imposed by *in vitro* oocyte maturation, *in vitro* fertilization (IVF) and *in vitro* embryo production) (El Hajj and Haaf, 2013). Under *in vitro* conditions, gametes and embryos are washed and exposed to incubations in different culture media, temperatures and oxygen concentrations (Ménézo et al., 2015; Ventura-Juncá et al., 2015). Previous studies have indicated that the dynamics of epigenetics markers during early embryo development are markedly affected by *in vitro* oocyte maturation and *invitro* embryo culture in various species, including mice, cows, pigs and humans (Abdalla et al., 2009; Cao et al., 2014; Heras et al., 2017; Lee et al., 2014; Maalouf et al., 2008; Nelissen et al., 2014; Salvaing et al., 2012; Santos et al., 2010). Of special relevance is the fact that metabolites present in the culture media can modulate the *in vitro* embryo development. Consequently, a lot of attention has been devoted to understanding embryonic metabolomics in the past decade (reviewed by Botros et al., 2008; Bracewell-Milnes et al., 2017; Krisher et al., 2015; Nel-Themaat and Nagy, 2011; Singh and Sinclair, 2007).

The importance of metabolites in regulating the cellular epigenome, inducing long-term changes to the cells, the so-called ‘metaboloepigenetic regulation’, has been reported in different cell types, such as stem cells and tumor cells (Donohoe and Bultman, 2012, reviewed by Etchegaray and Mostoslavsky, 2016; Reid et al., 2017; Van Winkle and Ryznar, 2019). More recently, studies have shown that epigenetic reprogramming might also be dependent on the metabolic pattern during the early stages of embryonic development (Ispada et al., 2020; Zhang et al., 2019). In this sense, understanding the metaboloepigenetic profile of *in vivo* embryos and how it differs from *in vitro* derived embryos in different species is essential to guiding future studies on modifying *in vitro* culture systems to improve embryo viability and progeny health. To this end, we analyzed RNAseq data of bovine, human and mouse *in vivo* and *in vitro* derived single oocytes (metaphase II) and early embryos (2-cell, 4-cell, 8-cell, 16-cell, morula and blastocyst) with emphasis on transcripts related to metabolic pathways that are known to influence cellular epigenetics. The correlations between transcripts and metaboloepigenetic pathways with DNA methylation are also presented.

## Results

### RNAseq data of bovine, human and mouse oocytes and embryos

Raw RNAseq data from oocytes (metaphase II) and embryos at different stages of development (2-cell, 4-cell, 8-cell, 16-cell, morula, and blastocyst) from *in vivo* and *in vitro* bovines and mice and from *in vitro* humans were obtained from the Gene Expression Omnibus data repository. To allow for inter-specific comparisons, raw sequence data were annotated and gene expression quantified using the Galaxy by NetworkAnalyst 3.0 web browser using identical parameters across all species (Zhou et al., 2019). We then used Probabilistic Quotient Normalization (PQN; Dieterle et al., 2006) to ensure the data were comparable across species and developmental stages. We identified a total of 16,207 bovine, 19,347 human and 22,941 mouse annotated genes in at least one developmental stage. 13,132 genes were common to all three species and 1,208, 3,747 and 7,520 genes were unique to bovines, humans, and mice, respectively (Figure S1). Only the 13,132 genes detected in all three species were used in subsequent analyses (Data S1). Since *in vivo* data from humans could not be obtained, we first compared *in vivo* samples of bovine and mouse and human *in vitro* samples (n = 50). Using these gene expression profiles, a random forest model classified species with an accuracy of 100% (Figure S1). For humans, the transitions 4–8C (11.94%) and 8C-MO (10.05%) were the stages with most differentially expressed genes (DEGs - Table S1 and Data S2). In bovine *in vivo* embryos, the highest number of DEGs were observed in the 4–8C (17.33%) and 16C-BL (3.56%) transitions. Mouse *in vivo* embryos had the highest number of DEG between MII-2C (26.53%), 2–4C (29.77%, Table S1), and 16C-BL (41.15%).

### Embryonic metaboloepigenetic profiles are dynamic and distinct in humans, mice and bovines

**Figure 1:**
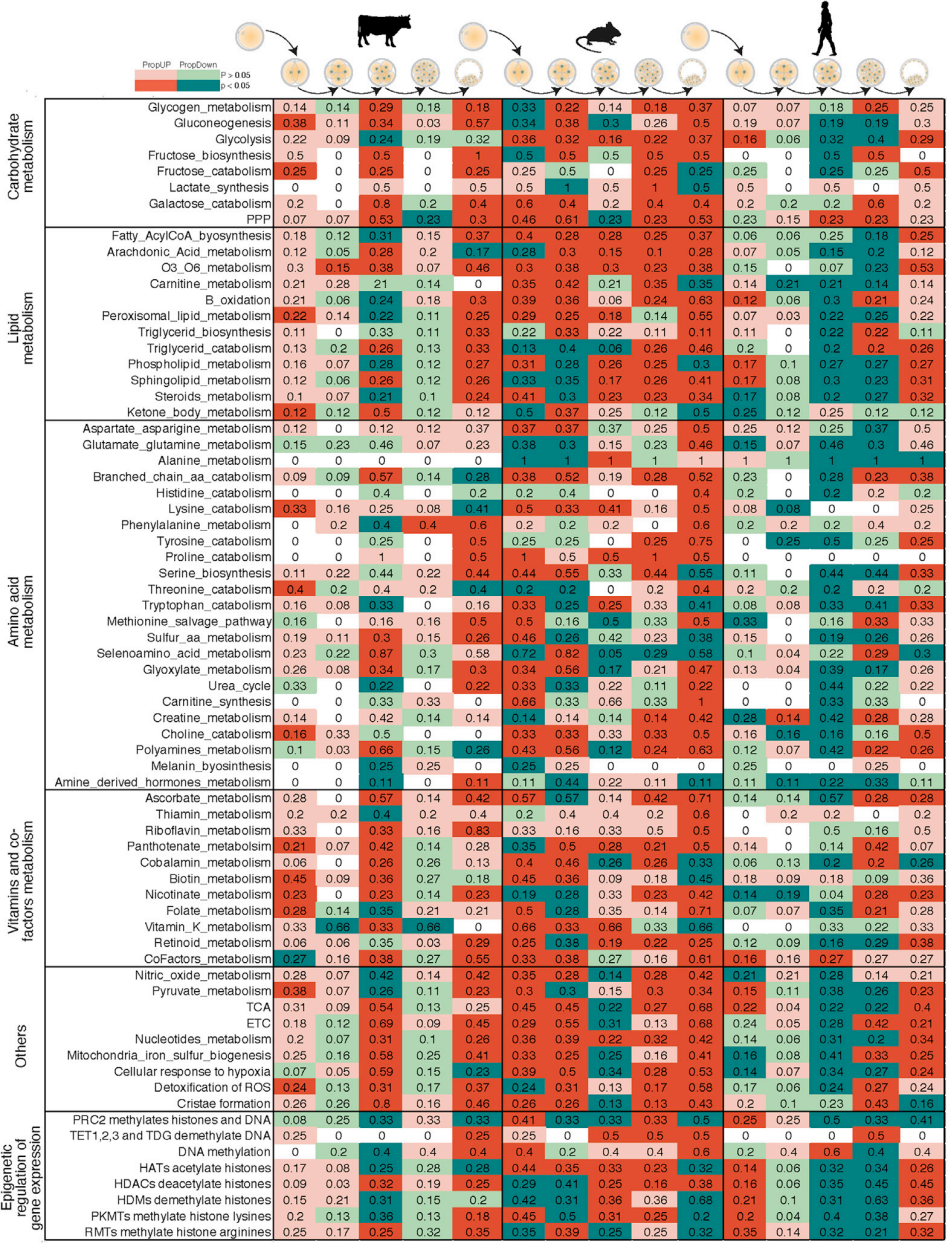
Metaboloepigenetic pathways are species and stage specific General analysis of human (*in vitro)*, mouse (*in vivo)* and bovine (*in vivo)* mature oocyte (MII) and embryos (2, 4, 8, 16C, MO and BL), showing proportion of up (PropUp, red) and down (PropDown, green) regulated metabolic and epigenetic pathways (part of Reactome terms “Metabolism” and “Epigenetic regulation of gene expression”) in 2C compared to MII, 4C compared to 2C, 8C compared to 4C, 16C compared to 8C and BL compared to 16C. Differences were calculated using ROAST, and significance determined using a one-sided directional p value < 0.05.

Metabolism is a relevant aspect for reprogramming and epigenetic control of cells. With that in mind, we performed RNAseq analysis on the different pre-implantation development stages with focus on 117 metabolic and epigenetic pathways (Data S3) detectable in the different stages and species both *in vitro* and *in vivo*. The rotation gene set testing (ROAST) tool (Wu et al., 2010) was used to assess the significance of changes in these metabolic and epigenetic pathways as a unit (differentially expressed pathways – DEP; Data S4). In bovine *in vivo* samples, corroborating the EGA that happens around the 8C stage (Graf et al., 2014a; Jiang et al., 2014), the 4–8C and 16C-BL transitions were the most variable (80 and 70% DEP, respectively; Figure 1 and Table S2). Similar to bovine, in *in vitro* human embryos, in which EGA occurs at 4–8C stage (Niakan et al., 2012), the majority of DEP were detected at the transition 4C–8C, 8C-MO and MO-BL (78%, 84 and 65%, respectively; Figure 1 and Table S2). Mouse samples were the most distinct, with 91% DEP observed in the MII-2C transition and 97% in the transition MO-BL (Figure 1 and Table S2).

**Figure 2:**
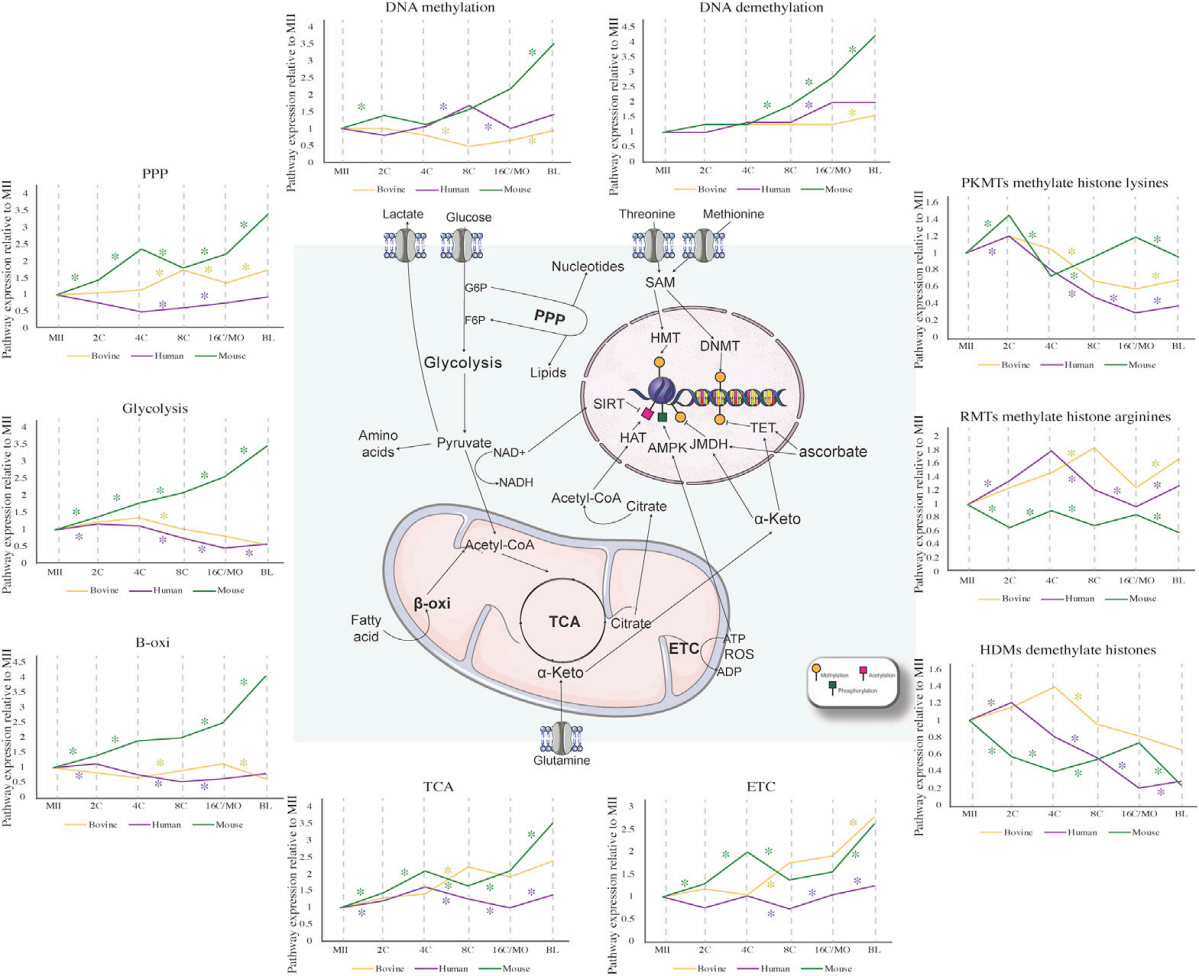
Changes on known metaboloepigenetic pathways are species and stage specific Analysis of human (*in vitro)*, mouse (*in vivo)* and bovine (*in vivo)* pre-implantation embryos (2, 4, 8, 16C/MO and BL). Known metaboloepigenetic pathways are depicted alongside each pathway expression pattern among the different pre-implantation stages (ROAST analysis data relative to MII expression) for human (purple), bovine (yellow) and mouse (green). Significant up and down regulated pathways between stages transition (MII-2C, 2C–4C, 4C–8C, 8C–16C/MO and 16C/MO-BL) intra-specie are marked with “∗” (one-sided p value < 0.05).

Close to the time of EGA and compaction in bovines and humans, embryos require more energy to support greater transcriptional activity, biosynthesis and cell proliferation, in addition to the blastocele formation and hatching, which marks the transition of an embryo composed of totipotent cells to a blastocyst containing pluripotent (inner cell mass –ICM) and differentiated (trophoblast – TB) cells. This increased metabolic demand can be seen by a change in the expression of the pentose phosphate pathway (PPP), glycolysis, beta-oxidation, electron transport chain (ETC, also known as oxidative phosphorylation) and the tricarboxylic acid cycle (TCA), which concurred with the reported changes in these metabolic pathways (Devreker, 2007; Gardner and Harvey, 2015; Guerif et al., 2013), specifically in the transition 4–8C in bovine and humans and 2–4C in mouse (Figure 2). Interestingly, in mice, this pattern was observed at MO-BL interval as well, which indicate that although DNA demethylation and major EGA occur at very early stages of development, the preferred metabolic pathways seem to follow the embryo's functional requirement for compaction and differentiation, and not the molecular demand.

Beyond changes in metabolic requirements, pre-implantation embryos also undergo a wave of global epigenetic reprogramming that is in agreement with the dynamic expression of DNA and histone (de)methylation pathways observed (Figure 2). Such dynamics were slightly different between species and likely related to the moment of EGA. In humans, the DNA methylation pathway starts increasing at the 4C, reaching its higher expression at the 8C, which is then reduced at the MO and maintained through the BL (Figure 2), while the DNA demethylation pathway is increased at the MO only (Figure 2). In mice, there was a significant increase in DNA methylation pathway expression at the 2C and at the BL stages (Figure 2). In contrast to humans, the mouse DNA demethylation pathway expression demonstrated a significant gradual increase from the 8C to the BL (Figure 2). In bovine, a decrease on DNA methylation was observed at the 8C, which was followed by an increase at the BL stage. Bovine DNA demethylation pathway only increased at the BL stage (Figure 2).

It has been previously shown that the lowest levels of global DNA methylation occur around the 16C stage for humans, bovines and mice (Duan et al., 2019; Graf et al., 2014b; Guo et al., 2014; Jukam et al., 2017). More specifically, human embryos exhibit low levels of DNA methylation after the first cell division and remain that way until the MO stage (Guo et al., 2014). In this case, the minor EGA is also initiated during the first cleavage while the major EGA takes place around the 8C stage (Jukam et al., 2017). In bovines the process is similar, with demethylation being more evident from 4C and resumed earlier, around the 16C stage (Duan et al., 2019). As no differences in expression of the DNA demethylation pathway were observed between the 2, 4 and 8C stages in neither humans nor bovines, nor at the 16C in bovines (Figure 2 and Data S4), it is likely that other players, such as metabolic factors, regulate such transitions in these species. As seen in Figure 2, the relationship between epigenetic events and metabolic aspects can occur at different levels within the cell.

Mouse embryos seemed to have the most distinct pattern. In this species, the global genome methylation is already low in 2C embryos in accordance with the major EGA, which also occurred at this stage. Unlike in humans and bovines, it was observed that mouse 8C, MO and BL stages have increased expression of the DNA demethylation pathway (Figure 2). This indicates that the action of demethylases was also responsible for such demethylation changes in mice. As for humans, *de novo* methylation will also be restored from the late BL (Jukam et al., 2017; Santos et al., 2002), which can be partially because of a significant increase on the expression of DNA methylation pathway at the BL stage in mice (Figure 2).

The dynamics of histone methylation are also crucial during gametogenesis and early embryogenesis, being the most abundant modification with the functional role dependent on the modified amino acid and the number of CH3 groups inserted (1, two or 3; Iwasaki et al., 2013; Ross et al., 2008; Sarmento et al., 2004; Torres-Padilla et al., 2007; Zhang et al., 2012), which leads to changes in the protein’s conformation, assembling or removing specific binding sites (Bannister and Kouzarides, 2011). For instance, H3K4 trimethylation (H3K4me3), primarily found around transcription initiate sites, is related to increased gene transcription and euchromatin formation (Sims et al., 2007; Wysocka et al., 2006), while H3K27 trimethylation (H3K27me3) leads to the opposite effect (Bártová et al., 2008). The patterns of H3K4me3 and H3K27me3 undergo dramatic changes during the initial embryogenesis, in accordance with its relevance for molecular events such as EGA and the establishment of totipotency. In the present work, we observed a significant decrease in the expression of the histone demethylases (HDMs) pathway during the transition 4–8C in bovine, while in humans this increased at MII-2C and at the MO-BL transitions and decreased at the 4–8C and 8C-MO transitions (Figure 2). In mice, the increase was observed in the transition 4–8C and decreased in MII-2C, 2C–4C and MO-BL transitions (Figure 2). Notably, it is clear from our findings that the embryonic metaboloepigenetic profile is a dynamic process, that varies not only inter-species, but also between the developmental stages intra-species.

To better elucidate these variations, we specifically characterized the patterns of transcripts belonging to methylation/demethylation of DNA and H3K4 and H3K27 residues, as well as the metabolic pathways known to be related to these processes: TCA cycle, one-carbon cycle (folate and methionine cycles) and methionine salvage pathway. The levels of these transcripts were analyzed in the MII-2C, 2C–4C, 4C–8C, 8C–16C/8C-MO and 16C-BL/MO-BL intervals in bovine and murine embryos produced *in vivo* and *in vitro* (except for mouse MII-2C *in vivo*), as well as in the MII-2C, 2C–4C, 4C–8C, 8C-MO and MO-BL intervals in human embryos produced *in vitro*. Human embryos are not discussed in detail because of the limited variation between the different stages, which can be explained by the greater variability and molecular heterogeneity caused by morphophysiological and chromosomal aberrations characteristic of human embryonic samples, which are, normally, surplus of IVF cycles and/or discarded samples (Liu et al., 2009).

### Mouse embryo metaboloepigenetic profile is dynamic between stages and varies between *in vivo* and *in vitro*

**Figure 3:**
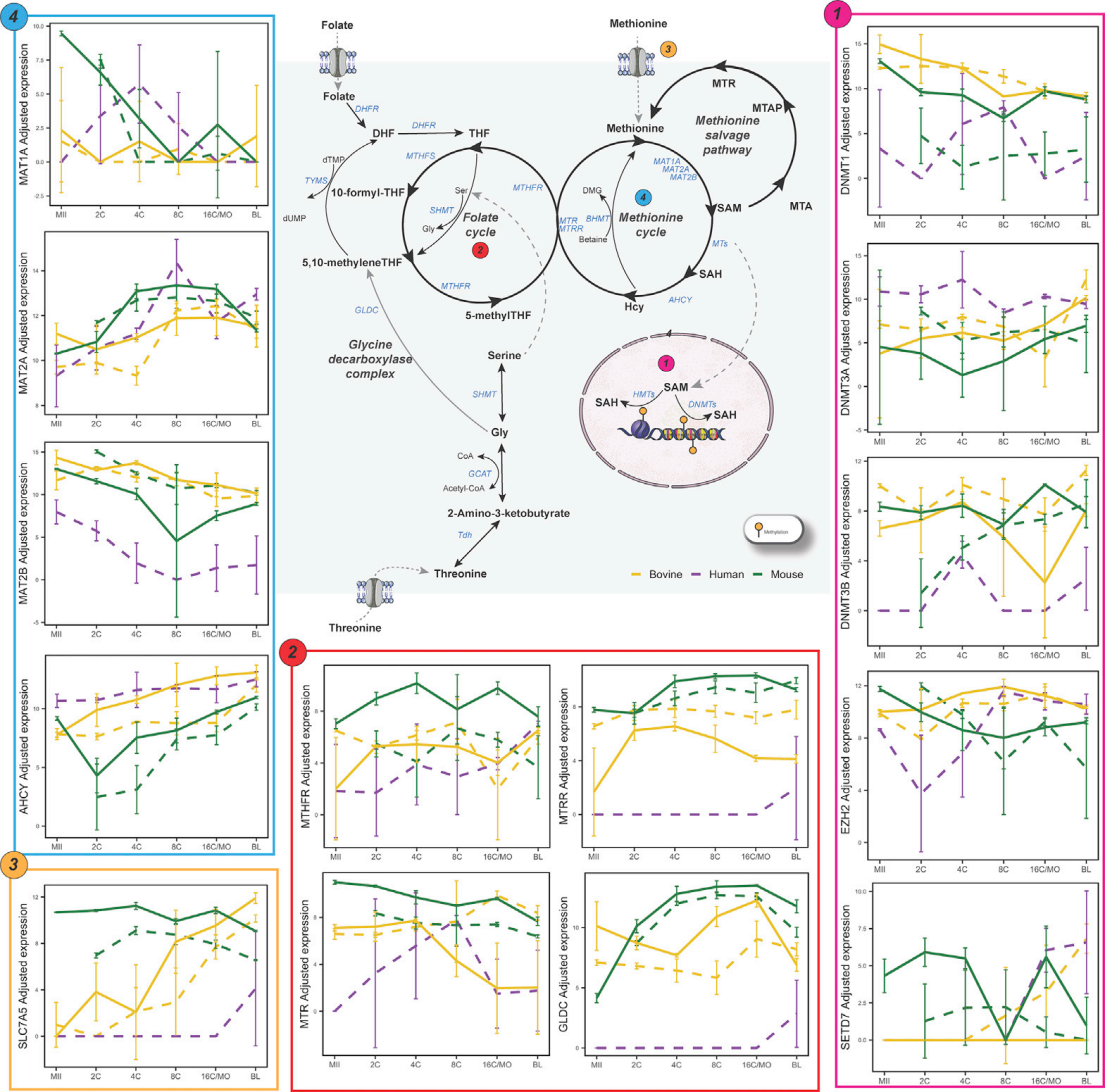
Metaboloepigenetic control of DNA and histone methylation Analysis of human (purple), mouse (green) and bovine (yellow) *in vivo* (full lines) and *in vitro* (dashed lines) pre-implantation stages (MII, 2, 4, 8, 16C/MO and BL). The connection between DNA and histone methylation pathways with the one carbon cycle are depicted alongside selected expression pattern among the different pre-implantation stages for each species of selected genes (mean ± SD). For significant up and down regulated genes between stages and species see Figure S2.

The relationship between methylation events and metabolic aspects can occur at different levels within the cells. The first is related to the generation of methyl donors. Although DNA and histone methylation processes are catalyzed by different sets of enzymes, both use a common methyl donor, S-adenosylmethionine (SAM) (Stover and Caudill, 2008). The synthesis of SAM involves the one-carbon cycle metabolism which integrates two main pathways: the folate cycle and the methionine cycle (Figure 3). In embryonic stem cells, the maintenance of pluripotency is dependent on H3K4me3 and this mark dramatically decreases when threonine is not available in the culture media, although DNA methylation and other lysine residues remain unchanged, suggesting that in these cells, threonine is metabolized to selectively promote H3K4me3 (Shyh-Chang et al., 2013). In addition, in mice, the restriction of methionine intake also triggers a decrease in H3K4 methylation, reinforcing the sensitive relationship between intracellular SAM levels and epigenetic control (Mentch et al., 2015) (Figure 3).

**Figure 4:**
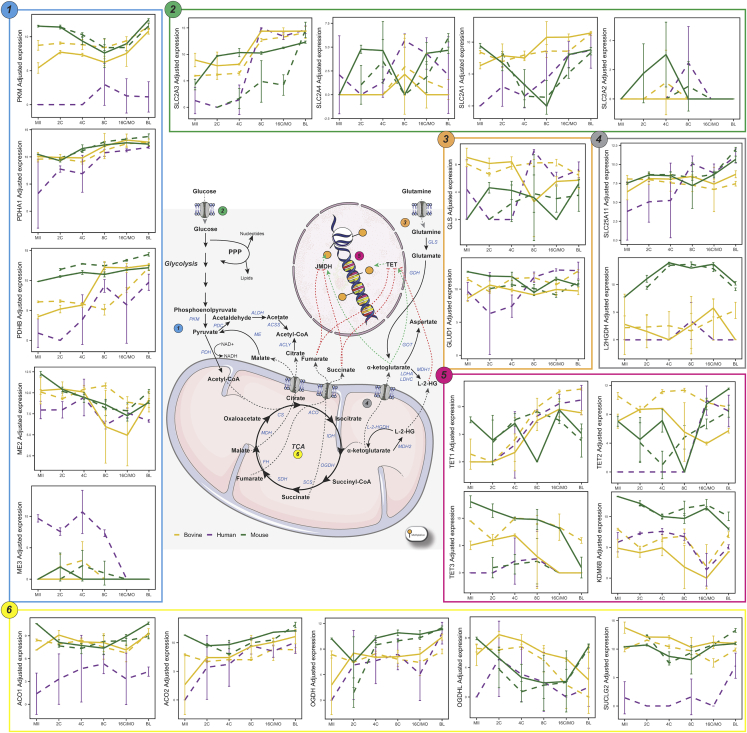
DNA and histone demethylation are controlled by metabolism Connection between metabolism and DNA and histone demethylation of human (purple), mouse (green) and bovine (yellow) *in vivo* (full lines) and *in vitro* (dashed lines) pre-implantation stages (MII, 2, 4, 8, 16C/MO and BL). Pathways related to glucose, pyruvate, α-ketoglutarate and glutamine, metabolism and the TCA cycle, that can directly influence DNA and histone demethylation are depicted alongside each gene expression (mean ± SD pattern among the different pre-implantation stages for each species. For significant up and down regulated genes between stages intra-species see Figure S3.

In addition to the relationship between metabolism and SAM generation, intermediates of metabolic pathways also interfere with the activity of enzymes responsible for DNA and histone demethylation (Figure 4). A key molecule in this process is α-ketoglutarate (α-KG), an intermediate in the tricarboxylic acid cycle. In embryonic stem cells the α-KG:succinate ratio affects pluripotency, and the accumulation of succinate and fumarate inhibits the enzymatic activity of TET demethylases, leading to higher levels of DNA methylation and, consequently, the maintenance of a more differentiated state. On the other hand, the high αKG:succinate ratio, results in greater TET activity and reduced DNA methylation, maintaining the less differentiated state. Similar to DNA demethylation, histone demethylase enzymes also use α-KG as a co-factor to remove methyl groups from histones residues. Although α-KG is crucial for histone demethylation, it has been shown that the accumulation of succinate within the cell may antagonize the activity of histone demethylases and promote cell differentiation from embryonic stem cells (Carey et al., 2015; Ispada et al., 2020; TeSlaa et al., 2016).

Regarding these related pathways, in mice we verified marked differences in transcripts levels already at the MII-2C interval, which can be explained by the EGA that occurs around the 2C stage in this species (26.53% DEG *in vivo*). More specifically, at the beginning of development, *in vivo* embryos present a global scenario of demethylation, both for DNA and K4 (already in 2C) and K27 (from 8C) residues of histone 3. This was corroborated by the higher levels of *Kdm6a*, a H3K27 demethylase. On the other hand, *Tet* levels remained unchanged and there was a decrease in the levels of transcripts related to H3K4 demethylation (*Kdm5a* and *Kdm5b*) (Figure 4). Therefore, changes in both the availability of co-factors for demethylases and methyl donors could explain these demethylation processes *in vivo*. In fact, there was an increase in *L2hgdh*, a FAD-dependent enzyme that oxidizes L-2-hydroxyglutarate to α-KG, together with an increase in *Gls* that catalyzes the conversion of glutamine in L-glutamate which can be further converted into α-KG, a co-factor of both TETs and JmjC-domain containing demethylases (Figure 4). A high α-KG:succinate ratio promotes DNA and histone demethylases activity, leading to chromatin modifications related to hyper-transcription in embryonic stem cells (Turner, 2008). This possible increase in demethylases activity would be accompanied by the lower availability of SAM, evidenced by a lower *Mtr, Mat1a* and *Mat1b* (Figure 3). The decline of *Mtr* decrease THR to SAM flux and, in addition to the decrease in *Mat1a* and *Mat1b*, there would be less generation of SAM, the main donor of methyl groups to methylations (Shyh-Chang et al., 2013).

During 2–4C interval, there was still an intense dynamic in the synthesis of transcripts *in vitro* and *in vivo* (45.87 and 29.77% DEG, respectively), which decreased in the 4–8C and 8C-MO intervals (*in vitro:* 7.22 and 4.68% and *in vivo*: 2.73 and 4.69%, respectively Table S1) suggesting a transcriptional stability phase for these pathways, which underwent marked changes again in the MO-BL range (*in vitro: 31.99*% and *in vivo*: 41.15% DEG). During BL formation, *in vivo*, several histone and DNA methylation processes reinstate, preferentially in the cells of the ICM. Supporting the observed increase in methylation, there was, in general, a reduction in transcripts for demethylases and an increase in transcripts for histone and DNA methylases, except for the increase in *Tet2* (Figure 4). Among the TET enzymes, TET3 is the most involved in the global genome demethylation during the earliest stages of development, while TET1 and, mainly, TET2 participate in the maintenance of pluripotency in cells of ICM (Ito et al., 2010; Koh et al., 2011), explaining the greater levels in this interval (Figure 4). Both for *in vivo* and *in vitro* BL there was an increase in *Ahcy*, which converts SAH, an inhibitor of methyltransferases, to homocysteine, preventing its accumulation (Figure 3). It is worth noting that, in several tissues, such as the liver, it is not the greater availability of SAM that creates a supportive environment for methylation, but rather the SAM:SAH ratio, the methylation index (Hoffman et al., 1980). Thus, for both *in vivo* and *in vitro* embryos, higher levels of *Ahcy* might be decreasing intracellular SAH, providing an environment more prone to methylation. Whereas in *in vivo* BL there was a decrease in *Mat2a* and an increase in *Mat2b*, in *in vitro* BL *Mat2a* remained unchanged and there was a decrease in *Mat2b*, favoring the synthesis of SAM, which could explain the hypermethylated DNA status of the latter in relation to the former (Wright et al., 2011).

### Before EGA bovine embryo metaboloepigenetic profiles remained stable *in vivo* but were divergent *in vitro*

In bovine embryos, active and passive DNA demethylation also occurs from the first cleavage, reaching its lowest levels between 8 and 16C (Duan et al., 2019). Although the major EGA already occurs at 2C in mice, in bovines this occurs only around 8C (Graf et al., 2014b). As expected, there were no marked differences in the transcript levels in MII-2C and 2–4C intervals for *in vivo* embryos (0.69 and 0.26% DEG, respectively), and it was only in the 4–8C interval that substantial differences could be identified (17.33% DEG; Table S1). The 8–16C interval showed minimal variation (0.43% DEG), which remained in the 16C-BL range (3.56% DEG).

In further contrast to mice, where differences in metaboloepigenetic transcripts were already observed in the early cleavage stages (MII-2C, 2C–4C and 4–8C), differences were mainly observed when early stages (MII/2C/4C) were compared to the BL stages in bovines, agreeing with the lower differences in number of DEG in early cleavage stages (MII-8C) in this species. The DNA demethylation that remarkably occurs until the 8-16C stage was confirmed by an increased expression of demethylation factors (*TET2* and *TET3*, Figure 4) and a decrease in the maintenance DNA methylase *DNMT1*, in early stages (MII and 4C) compared to the BL stage (Figure 3). Metabolically, a lower level of the methionine transport Solute Carrier Family seven Member 5 (*SLC7A5)*, was observed in early-stage embryos (MII, 2 and 4C) compared to BL (Figure 3), which would lead to lower methionine levels and, consequently, less SAM as substrate for DNA methylation.

Interestingly, *in vitro* embryos presented relevant changes in both the MII-2C and 2–4C intervals (35.18 and 34.66% DEG, respectively). More specifically, in the MII-2C interval *in vitro*, several enzymes from the TCA cycle appear upregulated, with the exception of *ACO*, that catalyzes the isomerization of citrate to isocitrate, and *OGDH*, that catalyzes the oxidative decarboxylation of α-KG to Succinyl-CoA. *GLS* and *GLUD1*, which participate in the conversion of glutamine and glutamate to α-KG, were downregulated, as well as several histone and DNA methylases (Figures 3 and 4, Data S2). Surprisingly, in the 2–4C interval *in vitro*, the global scenario was exactly the opposite, with TCA cycle enzymes being overexpressed, except *GLS*, *GLUD1* and *OGDHL*, while there was an overall increase in methylases and demethylases.

*In vitro* 8–16C interval showed slightly variation (4.49% DEG), which increased in the 16C-BL range (25.89% DEG). In the latter, there was a substantial increase in transcripts related to the TCA cycle. Still, there were lower levels of *MAT2A* and higher levels of *AHCY* (Figure 3), suggesting the lower synthesis of SAM, but still maintaining the favorable methylation environment, typical of this phase, which is corroborated by the increase in *DNMT3A* and *DNMT3B* (DNA *de novo* methylation; Figure 3 and Data S2). It is important to note that for H3K27, which also begins to be globally methylated in this phase *in vitro*, there was a decrease in *EZH2* methylase (Figure 3) and an increase in *KDM6B* demethylase (Figure 4), suggesting an additional control beyond the amount of enzymes for this methylation process. Furthermore, the decrease in *MAT2A* might explain the increase in hypomethylated genomic loci in embryos cultured *in vitro* when compared to *in vivo* (Salilew-Wondim et al., 2018).

Some important elements must be considered in relation to these results: i) there was an inverse relationship between the transcription of TCA cycle enzymes and those related to methylation/demethylation; ii) there was an increase in the levels of transcripts related to these pathways, which is not expected before the major EGA. The interpretation of this phenomenon in light of the metabolic and epigenomic changes that take place in this phase is quite challenging as the decrease in the amount of transcripts may be related to less transcription or greater translation. Nevertheless, the increase in transcripts levels before the major EGA for *in vitro* embryos and differently from *in vivo* embryos suggests a lack of molecular control with possible consequences to both the metabolic activity and the epigenome reprogramming. The earliest stages of development are extremely sensitive to the microenvironment. *In vivo*, this environment is highly influenced by the maternal state, while *in vitro*, the culture conditions have a remarkable impact on their development potential, impairing their developmental competence, and disturbing the subsequent maternal-embryonic communication, leading to failures in the establishment of pregnancy and long-term implications to the offspring (Krisher et al., 1999; Leroy et al., 2015; Lucy et al., 2014; Thompson et al., 1996; Velazquez, 2015).

This lack of molecular control may be a consequence of *in vitro* culture conditions which, despite presenting an acceptable relative efficiency, still report a high arrest rate up to 16C (Antunes et al., 2010). Thus, the inadequate supply of metabolites in culture media in supra physiological concentrations could require the embryo to activate metabolic pathways in advance, in an attempt to survive. In fact, when compared to oviduct and uterus fluids, SOFaa (Holm et al., 1999), a conventional culture medium for bovine embryos, offers lower amounts of glutamine but reaches five times more glutamate, three times more methionine, and almost twice more threonine (Hugentobler et al., 2007).

The results presented here point to differences in relation to the dynamics of transcript synthesis throughout development, characteristic of both species (mouse vs bovine) and microenvironment (*in vivo* vs *in vitro*). The latter, in particular, can be indicative of the molecular status of embryos and contribute to: i) the understanding of the effect of the environment on the metabolic, molecular and epigenomic status in the early stages of embryonic development; and ii) in the development of new strategies for assisted reproductive technologies (ART) protocols aimed at obtaining blastocysts that are increasingly similar to those *in vivo* for further transfer. Because of this importance, the impact of *in vitro* embryo production (IVP) on the embryonic metaboloepigenetic features were investigated in more detail.

### Metaboloepigenetic DEG and DEP are correlated with early embryo DNA methylation

**Figure 5:**
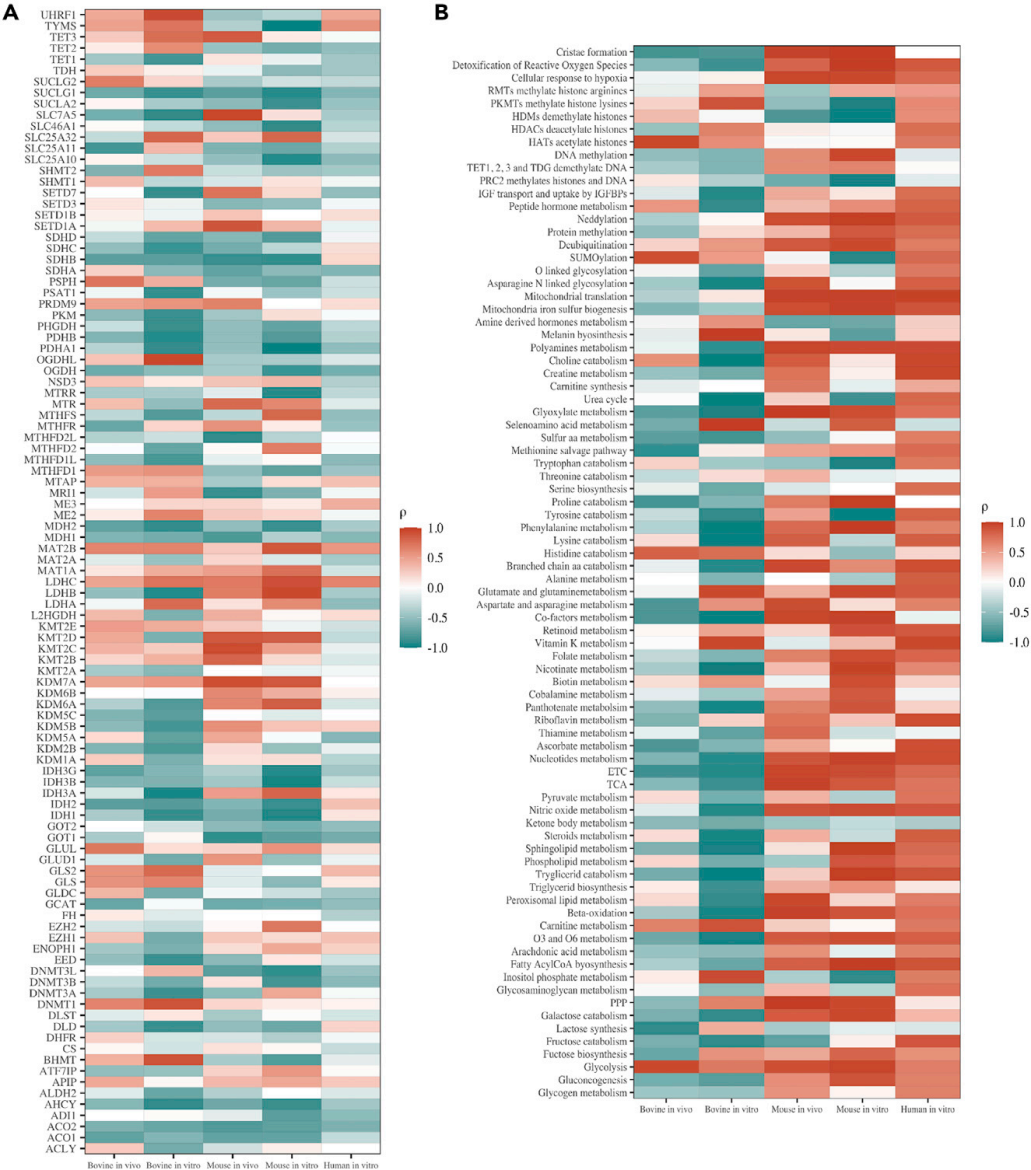
DNA methylation is correlated with metaboloepigenetic pathways and genes Correlation between DNA methylation and metaboloepigenetic genes expression (A) and metaboloepigenetic Reactome pathways (B) for bovine, human and mouse *in vivo* and *in vitro* pre-implantation embryos.

In an attempt to better evaluate the differences in metaboloepigenetic gene and pathway expressions among developmental stages, we evaluated the correlation between them with DNA methylation levels reported for bovine (Duan et al., 2019; Ivanova et al., 2020; Jiang et al., 2018), mouse (Guo et al., 2017; Shirane et al., 2013; Smith et al., 2012; Wang et al., 2014) and human (Guo et al., 2014; Zhu et al., 2018) *in vivo* and *in vitro* pre-implantation embryos (Figure 5 and Data S5).

Regarding Reactome metabolic and epigenetic pathways, bovine *in vivo* embryos had ten strong negatively correlated pathways (ρ<−0.7) with DNA methylation (Figure 5b and Data S5), worthy noting are “ascorbate metabolism” (ρ = −0.789), “ETC” (ρ = −0.850) and “methionine salvage pathway” (ρ = −0.885). Increased ascorbate metabolism can lead to higher ascorbate in the cell, which is known to stimulate TET activity, therefore the negative correlation with DNA methylation (Minor et al., 2013). Similarly, higher ETC pathway leads to increased ROS concentrations, which would deviate methionine from producing SAM to ROS scavenger, resulting in lower DNA methylation (Salilew-Wondim et al., 2018). Increasing methionine salvage pathway reduces availability of SAM to be used by methylases and also confirms the negative correlation with DNA methylation found. Moreover, four pathways exhibited a strong positive correlation (ρ> 0.7) with DNA methylation. Of note is the correlation between methylation and “glycolysis” (ρ = 0.937). These data, together with the lack of a strong correlation between DNA methylation and the Reactome pathways “DNA methylation” and “TET1, 2, and 3 and TDG demethylate DNA”, indicates that metabolism is a key regulator of epigenetic modifications in the early *in vivo* bovine embryo, which is also shown by the lack of strong correlation between DNA methylation and the expression of the DNA methylation genes *DNMT1*, *DNMT3a*, *DNMT3b*, *TET1*, *TET2*, and *TET3*.

On the other hand, *in vitro* bovine embryos had 33 strong negatively and eight strong positively correlated pathways with DNA methylation. Altogether, these data suggest that the *in vitro* environment plays an important role on embryo metabolism, with strong correlation to embryonic DNA methylation.

Mouse *in vivo* embryos, also lacked strong correlations between DNA methylation with the Reactome pathways “DNA methylation” and “TET1, 2, and 3 and TDG demethylate DNA” and the expression of the DNA (de)methylation genes *Dnmt1*, *Dnmt3a*, *Dnmt3b*, *Tet1* and *Tet2*. Nevertheless, 30 pathways exhibited strong positive correlations with DNA methylation whereas two pathways had strong negative correlations. This suggests that metabolism is also a key regulator of epigenetic modifications in the early *in vivo* mouse embryo. Of note is the strong positive correlation we observed between DNA methylation and the TCA (ρ = 0.974) and glycolysis (ρ = 0.913; Figure 5b and Data S5) Reactome pathways.

*In vitro*, mouse embryos showed 37 strong positively and nine strong negatively correlated pathways with DNA methylation, also demonstrating that the *in vitro* environment plays an important role on embryo metabolism, with strong correlation to embryonic DNA methylation. *In vitro* human embryos had 44 strong positively correlated pathways with DNA methylation. Similar to mouse and bovine embryos, human embryos also lacked strong correlations between DNA methylation with the “DNA methylation” and “TET1, 2, and 3 and TDG demethylate DNA” Reactome pathways as well as the expression of the DNA methylation genes *DNMT1*, *DNMT3a*, *DNMT3b*, *TET1*, *TET2*, and *TET3*, again demonstrating that metabolism is a key player for DNA methylation.

### *In vitro* environment impairs the metaboloepigenetic profile of pre-implantation embryos

During pre-implantation development, both metabolism and epigenetic reprogramming are considered to be extremely sensitive to changes in environmental conditions, such as those imposed by IVP systems. To better verify the impact of *in vitro* conditions on the proper epigenome reprogramming, we anew assessed the levels of transcripts related to DNA/histone methylation/demethylation, as well as those from metabolic routes related to these modifications. This time, the levels of these transcripts were analyzed throughout the development in mouse and bovine embryos, with special attention to the blastocyst stage.

**Figure 6:**
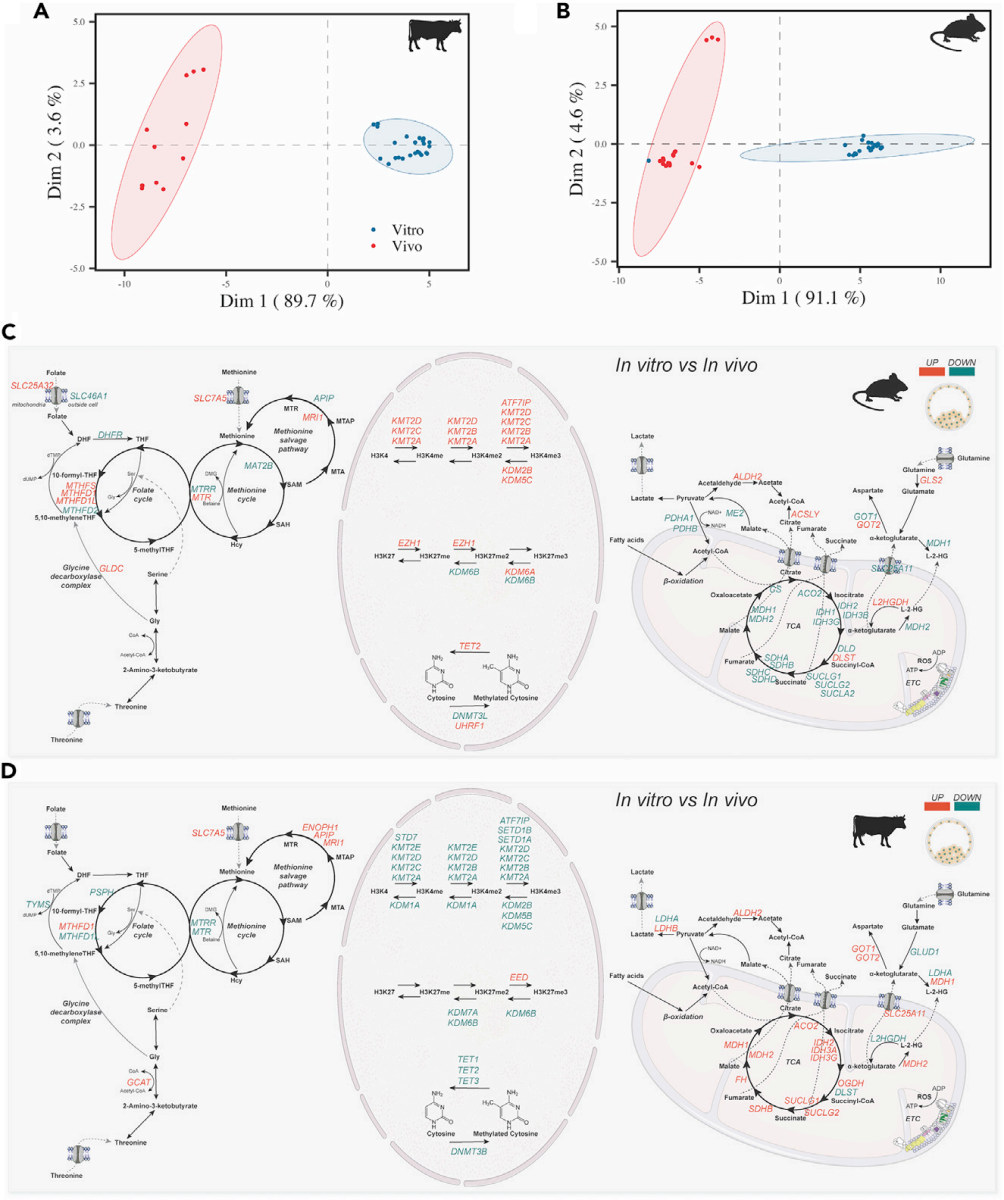
*In vitro* culture influences metaboloepigenetic genes expression (A–D) Embryo General analysis of *in vitro* bovine and mouse mature oocyte (MII) and embryos (2, 4, 8, 16C, MO and BL) gene expression of metabolism and epigenetic genes. PCA comparing bovine (A) and mouse (B) *in vivo* and *in vitro* samples. Metaboloepigenetic genes up (red) and down (green) regulated (adjusted p value < 0.05) comparing *in vitro* vs *in vivo* blastocysts from bovine (C) and mouse (D).

A clear distinction between *in vivo* and *in vitro* oocytes and embryos from different developmental stages was observed in both bovine and mice (Figures 6A, 6B, and S4). Unlike *in vivo*, *in vitro* bovine pre-implantation development presented three distinct segments: the first including the MII, 2, 4 and 8C stages; the second including the 16C stage; and the third including the BL stage (Figure S4). Similar to *in vivo*, *in vitro* mouse pre-implantation development presented three distinct segments: the first including the 2C, the second including the 4C, 8C and MO stages, and the third including the BL stage (Figure S4). The *in vivo and in vitro* observed differences of bovine and mouse pre-implantation development was supported by a random forest model, which classified the gene expression of 71 samples (32 from bovine and 39 from mice) with an accuracy of 100% for bovine and 97.5% for mice. Model diagnostics revealed that inter-species variation in the expression of *NRSN2*, *TXN* and *GSPT1* were the variables of primary importance for differentiating between collection method (*in vitro* v s*in vivo*; Figure S4). In contrast to *in vivo samples*, *in vitro* bovine embryos had the highest number of DEG between MII-2C (35.18%), 2–4C (34.66%) and 16C-BL (25.89%), while mouse *in vitro* had the highest number of DEG between 2 and 4C (45.87%) and MO-BL (31.99%, Table S1). Considering developmental stages individually, *in vitro* produced bovine and mouse embryos had, in average, 4.47 and 9.58% DEG, respectively, compared to *in vivo* (Table S3). MII and BL were the stages with more DEG in bovine (5.61 and 5.79%, respectively), while in the mouse the 2C, MO and BL had the most DEG (11.77, 12.58 and 12.65%, respectively; Table S3).

**Figure 7:**
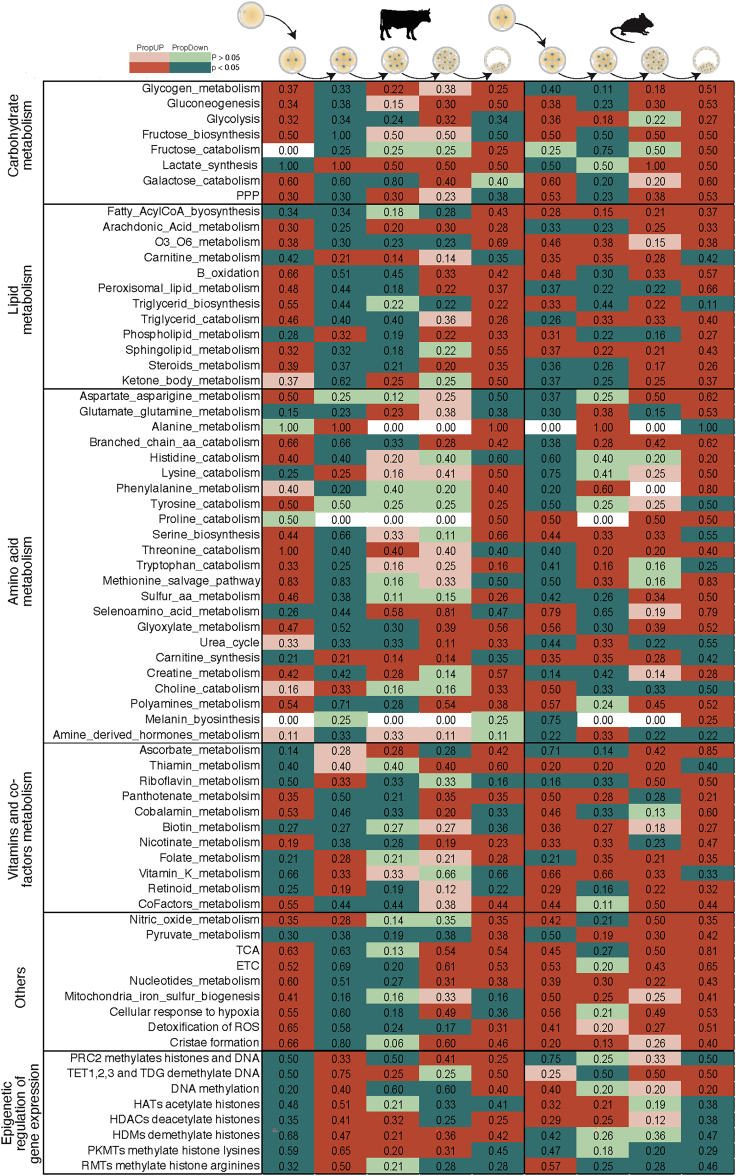
Metaboloepigenetic pathways are modified *in vitro* General analysis of *in vitro* bovine and mouse mature oocyte (MII) and embryos (2, 4, 8, 16C, MO and BL), showing proportion of up (PropUp, red) and down (PropDown, green) regulated metabolic and epigenetic pathways (part of Reactome terms “Metabolism” and “Epigenetic regulation of gene expression”) in 2C compared to MII (bovine only), 4C compared to 2C, 8C compared to 4C, 16C compared to 8C and BL compared to 16C. Differences on PropUp and PropDown were calculated using ROAST, and significance determined using a the one-sided directional p value < 0.05

DEG for individual stages were mainly intricated in the metaboloepigenetic pathways analyzed (Figure 7), with bovine *in vitro* having 85.34% of DEP and mouse *in vitro* having 85.21% of DEP (Table S4). In the bovine, BL was the stage with the highest DEP (93.97%), while in mouse it was the 2C (96.52%; Table S4). Independent of the developmental stage, the majority of metabolic pathways that were up-regulated in bovine *in vitro*, were down regulated in mice *in vitro*: such as TCA, ETC, methionine salvage pathway, gluconeogenesis, beta-oxidation, glutamate and glutamine metabolism, pyruvate metabolism, among others (Figure S5). In contrast, epigenetic pathways were mainly down regulated in bovine *in vitro* and up regulated in mouse *in vitro,* also independently of the developmental stage (Figure S5). These results indicate that the *in vitro* environment has an important effect on the metaboloepigenetic control of bovine and mouse embryos. Moreover, it corroborates the fact that bovine and mouse embryos have different developmental kinetics and are, therefore, differentially affected by the *in vitro* environment.

**Table 1:** Oocyte and embryo developmental stage, method of collection and number of replicates for RNAseq

		MII	2C	4C	8C	16C	MO	BL	Total
Bovine	*In vivo*	2	2	2	2	2		2	12
	*In vitro*	3	3	3	3	3		9	24
Mouse	*In vivo*	2	4	4	3		2	3	18
	*In vitro*		4	4	3		6	4	21
Human	*In vitro*	3	3	5	3		3	3	20
	Total	10	16	18	14	5	11	21	95

**Table 2:** Oocyte and embryo developmental stage, method of collection and sequencing for DNA methylation

Specie	Stage	Collection	Sequencing method	Equipment	Reference
Bovine	MII, 2, 4, 8 and 16C	*In vivo*	WGBS	Illumina HiSeq4000	(Duan et al., 2019)
Bovine	MII, 2C, 4C 8C, 16C and BL	*In vitro*	post-bisulfite adapter tagging (PBAT)	Illumina HiSeq 1000	(Ivanova et al., 2020)
Bovine	MII, 2, 4, 8C, BL	*In vivo*	Reduced representation bisulfite sequencing (RRBS)	Illumina Hiseq2500	(Jiang et al., 2018)
Human	MII, 2, 4, 8C, MO, ICM and TE	*In vitro*	Single-cell whole-genome bisulfite sequencing WGBS)	Illumina HiSeq 2500 or HiSeq 4000	(Zhu et al., 2018)
Human	MII, 2, 4, 8C, MO and ICM	*In vitro*	WGBS	HiSeq2000 sequencer	(Guo et al., 2014)
Mouse	MII	*In vivo*	WGBS were prepared using the PBAT method	Illumina HiSeq 2000	(Shirane et al., 2013)
Mouse	MII and 2C	*In vivo*	MethylC-Seq	Illumina HiSeq 2000	(Wang et al., 2014)
Mouse	2, 4, 8C and ICM	*In vitro*	Reduced representation bisulfite sequencing	Illumina Genome Analyzer IIx	(Smith et al., 2012)
Mouse	MII, 2, 4, 8C, MO, ICM and TE	*In vivo*	Single-cell multi-omics sequencing technology (single-cell COOL-seq)	Illumina HiSeq 2500	(Guo et al., 2017)

Mouse blastocysts produced *in vitro* also showed several metabolic indicators that confirmed their hypermethylated DNA status compared to blastocysts produced *in vivo* (Wright et al., 2011) since this change was not explained by differences in transcripts related to methylases and demethylases (Figure 6C). Among them, we point out the highest amount of transcripts of the folate cycle and those connecting the folate cycle to methionine cycle, such as *Mthfs, Mthfd, Glcs* and *Mtr* (Figure 6C). In addition, *in vitro* embryos had higher levels of *Slc7a5*, a methionine transporter, and *Ahcy,* together with lower levels of *Mat2b*, reinforcing this environment more conducive to methylation (Figure 6C). Indeed, when correlating gene expression with DNA methylation levels, *Ahcy* had a strong negative correlation (ρ = −0.875) with *in vitro* mouse DNA methylation and *Mat2b* had a strong positive correlation (ρ = 0.879), whereas no strong correlation (−0.7 >ρ< 0.7) was observed between DNA methylation with DNA demethylases (*Tet1, Tet2 and Tet3*) and DNA methylases (*Dnmt1 and Dnmt3a*) expressions (Figure 5A and Data S5).

In the case of bovine blastocyst, similarly, the differences in methylation reported in the literature were not explained by differences in the transcripts of methylases and demethylases (Figure 6D), which were not strongly correlated (−0.7 >ρ< 0.7) with DNA methylation *in vivo* (Figure 5A) and had contradictory correlation with DNA methylation *in vitro*: strong positive correlation for *DNMT1* (ρ = 0.899) and *TET3* (ρ = 0.763), with strong negative correlation for *DNMT3A* (ρ = −0.862) and *TET1* (ρ = −0.824) (Figure 5A and Data S5). In this species, however, unlike the mouse, there were lower levels of *MTR* and *MTRR*, despite the increase in *GCAT*, *SLC7A5* and transcripts of the methionine salvage pathway (Figure 6D). This scenario could indicate an active metabolism of methionine, however not necessarily related to methylation of DNA and histones. Methionine is a direct target of reactive oxygen species, acting as scavenger and protecting cells from oxidative stress (Luo and Levine, 2009). ART protocols are known to induce higher oxidative stress because the gametes and embryos must be manipulated during maturation, fertilization and embryo development in environments that generate reactive oxygen species (ROS) (Torres-Osorio et al., 2019), which could lead to methionine deviation for ROS scavenger, decreasing the availability of SAM for methylation of DNA and histones and conferring a hypomethylated status to these embryos (Salilew-Wondim et al., 2018). This deviation to ROS scavenger can be substantiated by an increase in the metabolic pathway “detoxification of ROS” not only in *in vitro* produced BL, but also in MII, 4, 8 and 16C embryos (Figure S5).

We also observed a relationship between TCA cycle transcripts and those related to epigenetic events. In the case of *in vitro* mouse blastocysts, 16/20 transcripts from the TCA cycle were downregulated and 11/30 methylase and demethylase transcripts were upregulated when compared to *in vivo* ones (Figure 6C). It was also seen in a reduction of the metabolic pathway “TCA” in all developmental stages and in an increase in the epigenetic pathway “TET1, 2, and 3 and TDG demethylate DNA” and in the “DNA methylation” in all but the 8C stage (Figure S5). In bovine blastocysts, this relationship was reversed; *in vitro* blastocysts had 12/20 transcripts from the TCA cycle upregulated and 19/30 methylase and demethylase transcripts downregulated when compared to their *in vivo* counterparts (Figure 6D). Oppositely to mouse, these changes could be corroborated by an increase in the metabolic pathway “TCA” in all but the 2C stage, and a reduction in the epigenetic pathway “TET1, 2, and 3 and TDG demethylate DNA” in all stages (Figure S5). A comparison of the *in vivo* versus *in vitro* 2, 4, 8 and 16C/MO metaboloepigenetic genes for mice and bovines can be seen in Figures S6 and S7, respectively.

These data point to metabolism, especially pathways related to the TCA cycle, as an unequivocal regulator of epigenetic processes in early embryos beyond the synthesis of succinate or α-KG, as already described for other cells and tissues. For instance, acetyl-CoA, generated from oxidation of pyruvate, fatty acid oxidation or amino acid degradation, acts as an acetyl donor-group for histone acetylation, affecting global histone acetylation and gene expression, with possible consequences to the methylation status (Moussaieff et al., 2015). Also, 2-Hydroxyglutarate, an α-KG derived metabolite increased in hypoxic conditions, targets α-KG-dependent dioxygenases, inhibiting their function (Xu et al., 2011). Fumarate, another intermediate of TCA cycle, causes DNA hypermethylation by inhibiting TET activity (Sciacovelli et al., 2016). These data reinforce the environment in which embryos are produced as essential for proper metabolic control, which increasingly impose themselves as a determinant of the epigenomic control of the embryo.

## Discussion

As aptly stated by (Harvey et al., 2016), “metabolism is at the heart of cell-sensing mechanisms”. Beyond simply providing ATP to maintain homeostasis and cell replication, metabolism generates intermediate products that form the basic building blocks for cell proliferation, and modulate signaling pathways and gene expression (reviewed by Donohoe and Bultman, 2012; Harvey et al., 2016; Milazzotto et al., 2020; Spyrou et al., 2019). Further, metabolites have the capacity to regulate the cellular epigenome, inducing long-term changes to cells via a process known as metaboloepigenetic regulation (Donohoe and Bultman, 2012). Here, we assembled a collection of embryonic RNAseq data in order to understand the molecular control of the metaboloepigenetic profile of bovine, mouse, and human *in vitro* and *in vivo* pre-implantation embryos. Our analyses have shown that the embryonic metaboloepigenetic profile is unique for each species, is highly dynamic between developmental stages, and is modified in *in vitro* cultured embryos.

The idea that the environment where oocytes and embryos develop may influence their outcome is certainly not new. Strategies to boost the numbers of transferable embryos by altering the availability of molecules in culture media in a search for the right formulations or culture conditions that mimic conditions found inside the reproductive tract have been an active area of research since the development of IVF 43 years ago (Cebrian-Serrano et al., 2013; Ferraz et al., 2018; Leese et al., 2008; Menezo et al., 2018; Sturmey et al., 2009). Despite decades of research, however, there have been only limited improvements in terms of developmental rates pre- and post-transfer (Simopoulou et al., 2018). Furthermore, although live births from ARTs (such as hormonal stimulation, embryo production by intracytoplasmic sperm injection and IVF) are now routine, ARTs are increasingly being shown to lead to postnatal ailments including premature births, delayed development, and metabolic disorders such as insulin resistance, and increased fasting blood glucose (Chen et al., 2013, 2014; Coussa et al., 2020). Consequently, understanding the molecular events that coordinate early embryonic development may assist in determining important pathways and processes for this moment, as well as identifying changes induced by ART (Menezo et al., 2018). Of special relevance is the fact that metabolites have been shown to modulate the *in vitro* embryo development, and a lot of attention has been devoted to understanding embryonic metabolomics in the past decade (reviewed by Botros et al., 2008; Bracewell-Milnes et al., 2017; Krisher et al., 2015; Nel-Themaat and Nagy, 2011; Singh and Sinclair, 2007). Interestingly, our findings have shown that, while the average number of DEG between *in vivo* and *in vitro* embryos were relatively low (4.47 and 9.58% for bovines and mice, respectively), these DEG were implicated in metaboloepigenetic pathways, accounting for an average difference of more than 80% DEP for both bovines and mice. These results demonstrate just how sensitive embryonic metabolism is to environmental conditions.

With these findings in mind, it is striking to consider that, even after four decades effort, most of the protocols for embryo culture still rely on a one-step supply of nutrients. Consequently, the dramatic differences we noted between the metaboloepigenetic profiles of *in vivo* and *in vitro* embryos was not overly surprising. As shown here, the embryonic metaboloepigenetic profile is stage dependent, which is not represented by current static IVP protocols. Moreover, our findings clearly show that the mechanisms leading to an incorrect epigenome reprogramming after IVF are under metabolic control. Thus, IVP protocols should also consider the dynamic nature of metabolic profiles in order to further improve their success. This is especially important given that cell metabolism, which has been remarkably affected trough changes in ART systems, seems to be the key in embryo epigenetic reprograming. To this end, and perhaps more important than accurately reproducing the physiological environment, we suggest that the focus should be on identifying systems and/or protocols that allow the embryos to respond the same way they would normally do *in vivo*. The embryonic metaboloepigenetic profiles mapped here are fundamental to developing new dynamic protocols that recreate the *in vivo* metaboloepigenetic profile, becoming a unique tool for the future of *in vitro* production of embryos and the birth of healthy offspring.

### Conclusions

The dataset compiled, normalized, and made comparable, represents a comparison of metabolic and epigenetic pathways in MII oocytes, 2-cells, 4-cells, 8-cells, 16-cells/morula and blastocysts of three different species and two conditions (*in vivo* versus *in vitro*). Our findings shows that bovine, human and mouse embryos present a species specific dynamic of metaboloepigenetic related transcriptional profile, which follows embryo's molecular and functional requirements. Our findings also show how metabolic pathways and their corresponding genes are correlated with DNA methylation. It was notable that the *in vitro* environment has an important effect on the metaboloepigenetic control of bovine and mouse embryos, showing that metabolism, is an unequivocal regulator of epigenetic processes in early embryos.

### Limitations of the study

For ethical and legal reasons, collecting *in vivo* human embryos is not an option. All of our analyses in humans were therefore based on *in vitro* embryos, which are known to be different from *in vivo*. Our RNAseq analysis was performed by curating available raw RNAseq data obtained from the Gene Expression Omnibus (GEO). As such, it is likely that there was noise in the data related to different embryo collection, culture, RNA extraction protocols, library preparation, and RNAseq analysis. Although this limitation was mostly overcome via our data normalization protocols, a fully controlled study would have ensured that this noise was minimized. Furthermore, while this represents the largest collection of embryos RNAseq data compared to date, the number of samples per cell stage within each species were limited. As such, small sample sizes limited the scope and scale of the analysis and our ability to accurately detect DEG. Therefore, our findings may include false negatives/positives for both DEG and DEP. Despite these limitations, the present results have an invaluable implication to the advances of the IVF field.

## STAR★Methods

### Key resources table

**Table:** 

REAGENT or RESOURCE	SOURCE	IDENTIFIER
**Deposited data**		

Bovine *in vivo* RNAseq raw data	Gene Expression Omnibus (GEO) Jiang et al., 2014	GSE59186
Bovine *in vitro* RNAseq raw data	Gene Expression Omnibus (GEO) Chitwood et al., 2013 and Graf et al., 2014a	GSE44023, GSE121227∗ and GSE52415
Mouse *in vivo* RNAseq raw data	Gene Expression Omnibus (GEO) Wang et al., 2018 and Wu et al., 2018	GSE97778 and GSE113164
Mouse *in vitro* RNAseq raw data	Gene Expression Omnibus (GEO)	GSE159484∗
Human *in vitro* RNAseq raw data	Gene Expression Omnibus (GEO) Xue et al., 2013 and Dang et al., 2016	GSE44183 and GSE71318

**Software and algorithms**		

R software	R Core Team (2020)	https://www.R-project.org/
Raw data importing, genome alignment and counting using Galaxy by NetworkAnalyst 3.0 web browser	Zhou et al., 2019	https://galaxy.networkanalyst.ca/
RNAseq quantification using Galaxy by NetworkAnalyst 3.0 web browser function Kallisto	Bray et al., 2016	https://galaxy.networkanalyst.ca/
Limma analysis using R	Ritchie et al., 2015	R package limma
ROAST analysis using R	Wu et al., 2010	mroast() function from the limma R package
PQN analysis using R	Dieterle et al., 2006	DOI: https://doi.org/10.1021/ac051632c
Random Forest model analysis using R	Ho, 1995	R package randomForest (ver. 4.6-14; RColorBrewer)

**Other**		

Compilation of genes part of Reactome metabolic and epigenetic pathways	Jassal et al., 2020	https://reactome.org/
R code necessary to reproduce RNAseq and Pearson correlations analyses	This paper	Data S6

∗Unpublished

### Resource availability

#### Lead contact

Further information and requests for resources should be directed to and will be fulfilled by the lead contact, Marcia de Almeida Monteiro Melo Ferraz (m.ferraz@lmu.de).

#### Materials availability

This study did not generate new unique reagents.

### Experimental models and subject details

All experimental models from data mined and used here are presented in the original articles and the GEO entries. Briefly, embryos were *in vivo* collected or *in vitro* produced from human - *Homo sapiens* (Dang et al., 2016; Xue et al., 2013), bovine - *Bos taurus* (Chitwood et al., 2013; Graf et al., 2014a; Jiang et al., 2014) and mouse - *Mus musculus* (Wang et al., 2018; Wu et al., 2018).

### Method details

#### Data mining

RNAseq raw data were downloaded from Gene Expression Omnibus (GEO), accession numbers are described in the key resources table. Raw data was imported using Galaxy by NetworkAnalyst 3.0 web browser (https://galaxy.networkanalyst.ca/) (Zhou et al., 2019). Abundance of RNAseq transcripts was quantified using the function Kallisto (Bray et al., 2016). Genes with low counts (<5) were removed. For further analysis, only genes that were expressed in at least one stage of each species were used (13,132 genes).

#### RNAseq data normalization

Data were transformed using EdgeR: log2(CPM+c) (c = 4). These data were obtained from GEO, and, as such, it is likely that there was noise in the data related to different embryo collection, culture, and RNA extraction protocols, library preparation, and RNAseq analysis. To render the data comparable across species and developmental stages, EdgeR transformed data were scaled using Probabilistic Quotient Normalisation (PQN; (Dieterle et al., 2006)), which calibrates individual gene expression profiles against the median profile. Notably, analyses on PQN transformed data have been shown to have low false-positive rates, and can accurately recover groups of interest without introducing artefactual differences (Noonan et al., 2018).

#### Differentially expressed genes analysis

Differentially expressed genes (DEG) of normalized data were identified using the R package limma (Ritchie et al., 2015). An adjusted p-value to correct for multiple testing was calculated using the Benjamini–Hochberg method (Benjamini and Hochberg, 1995) that aims to control the false discovery rate across significant genes and is the most widely used correction for genomic studies. The R code necessary to reproduce these analyses is presented in Data S6.

#### Epigenetic and metabolic genes analysis using the rotation gene set testing

We accessed information of all epigenetic and metabolic pathways for humans (Reactome terms: “epigenetic regulation of gene expression”,“metabolism”,“metabolism of proteins” and “metabolism of RNA”) from the Reactome pathways database version 74 (Jassal et al., 2020). Reactome pathways are arranged into several tiers, the Reactome term “epigenetic regulation of gene expression” (Reactome ID: R-HSA-212165.2), included curated pathways involving 122 genes; the Reactome term “metabolism” (Reactome ID: R-HSA-1430728.10) involved 2,210 genes; the curated pathways of the Reactome term “metabolism of proteins” (Reactome ID: R-HSA-392499.7) involved 2,095 genes; and the curated pathways of the Reactome term “metabolism of RNA” (Reactome ID: R-HSA-8953854.4) involved 739 genes. Rotation gene set testing (ROAST) was used to perform self-contained gene set analysis of each metabolic pathway, for the different developmental stages and species (Wu et al., 2010). The ROAST analysis was performed using the mroast() function from the limma R package and the R code necessary to reproduce these analyses is presented in Data S6.

### Quantification and statistical analysis

Number of samples per species, collection method and stage used are depicted in Table 1. For DEG, an adjusted p value < 0.05 was considered significant. For DEP, an one-sided directional p-value < 0.05 was considered significant.

A random forest (RF) model (Ho, 1995) was used to classify intra- and inter-species gene expression profiles according to developmental stages and collection method (*in vivo* or *in vitro*), with scaled gene expression values as the prediction variables. These analyses were conducted using the R package randomForest (ver. 4.6-14; RColorBrewer) (Liaw and Wiener, 2018). We chose RF modeling as it does not require any parameter reduction prior to analysis (Cutler et al., 2007), and has been shown to provide reliable results for biomarker identification (Chen et al., 2017), particularly on PQN transformed data (Noonan et al., 2018). We used the RF variable importance values to identify the key genes important for classifying groups of interest (i.e., species, collection type, developmental stage) in each RF model.

We also calculated Pearson correlation coefficients between the proportion of methylated genes (mined from data shown in Table 2) and both gene expression values and the proportion of up/down regulated genes in the Reactome pathways across the different cellular stages.

The R code necessary to reproduce these analyses is presented in Data S6.

## Data Availability

• This paper analyzes existing, publicly available data. All RNA-seq data were obtained from GEO and accession numbers are listed in the key resources table (Chitwood et al., 2013; Dang et al., 2016; Graf et al., 2014a; Jiang et al., 2014; Wang et al., 2018; Wu et al., 2018; Xue et al., 2013). Oocyte and embryonic stages and number of samples are depicted in Table 1. Overview of culture conditions used for *in vitro* embryo production, of embryos which data were used in the present study are depicted on Table S5. • The normalised data are included as Data S1. The DNA methylation data were obtained from the published sources listed in Table 2. • All original code is available in this paper’s supplemental information. • All software used are freely available and are listed in the key resources table. • This paper does not report original code and any additional information required to reanalyze the data reported in this paper is available from the lead contact upon request.

## Supplemental information

Document S1. Figures S1–S7, Tables S1–S5, and Data S6

Data S1. Normalized expression value from the current analysis of all GEO datasets, related to Figures 3, 4, and 6

Data S2. Log2 fold change, p-values and adjusted p-values for all differently expressed genes, related to Figures 3, 4, and 6

Data S3. Metabolic and epigenetic Reactome terms used for ROAST analysis, related to Figures 1, 2, and 7

Data S4. Full ROAST analysis data, related to Figures 1, 2, and 7

Data S5. Correlations between gene expression and Reactome pathways with DNA methylation, related to Figure 5
