# Supplementary material for: Mining RNAseq data reveals dynamic metaboloepigenetic profiles in human, mouse and bovine pre-implantation embryos
Source: iScience. 2022 Feb 11;25(3):103904. doi: 10.1016/j.isci.2022.103904 (PMC8889150; doi:10.1016/j.isci.2022.103904)
Supplement: Document S1. Figures S1–S7, Tables S1–S5, and Data S6 [file mmc1.pdf]

iScience, Volume *25*

Supplemental information

Mining RNAseq data reveals dynamic metaboloepigenetic profiles in human, mouse and bovine pre-implantation embryos

Marcella Pecora Milazzotto, Michael James Noonan, and Marcia de Almeida Monteiro Melo Ferraz

Table S1. Overview of up and down significant differentially expressed genes (DEG) from Limma analysis of *in vivo* and *in vitro* pre-implantation development stages (adjusted p-value < 0.05); related to Figures 1 and 7. Up: number of up-regulated pathways; Down: number of down regulated pathways; NS: number of non-significant pathways.

Species	Collection	Stage	Up	Down	NS	DEG (%)	Average DEG (%)
**Human**	in vitro	*2C-MII*	135	25	12972	1.22	5.6
		4C-2C	6	110	13016	0.88	
		8C-4C	141	1427	11564	11.94	
		MO-8C	348	972	11812	10.05	
		BL-MO	419	91	12622	3.88	
**Bovine**	in vivo	*2C-MII*	88	3	13041	0.69	4.45
		4C-2C	27	7	13098	0.26	
		8C-4C	1151	1125	10856	17.33	
		16C-8C	30	26	13076	0.43	
		BL-16C	271	196	12665	3.56	
	in vitro	*2C-MII*	2284	2336	8512	35.18	20.97
		4C-2C	2373	2178	8581	34.66	
		8C-4C	400	208	12524	4.63	
		16C-8C	269	320	12543	4.49	
		BL-16C	1664	1736	9732	25.89	
**Mouse**	in vivo	*2C-MII*	2007	1477	9648	26.53	21.37
		4C-2C	2035	1875	9222	29.77	
		8C-4C	480	135	12517	4.68	
		MO-8C	494	122	12516	4.69	
		BL-MO	2736	2668	7728	41.15	
	in vitro	*4C-2C*	2880	3143	7109	45.87	21.95
		8C-4C	561	387	12184	7.22	
		MO-8C	217	142	12773	2.73	
		BL-MO	2391	1810	8931	31.99	

Table S2. Overview of up and down differentially expressed pathways (DEP) from ROAST analysis of *in vivo* and *in vitro* pre-implantation development stages (one-sided directional p-value < 0.05); related to Figures 2 and 7. Up: number of up-regulated pathways; Down: number of down regulated pathways; NS: number of non-significant pathways.

Species	Collection	Stage	Up	Down	NS	DEP (%)	Average DEP (%)
**Human**	*in vitro*	*2C-MII*	24	21	71	38.79	55.17
		4C-2C	2	8	106	8.62	
		8C-4C	4	87	25	78.45	
		MO-8C	30	68	18	84.48	
		BL-MO	67	9	40	65.52	
**Bovine**	*in vivo*	*2C-MII*	15	2	99	14.66	34.07
		4C-2C	0	1	115	0.86	
		8C-4C	64	29	23	80.17	
		16C-8C	2	2	112	3.45	
		BL-16C	58	24	34	70.69	
	in vitro	*2C-MII*	66	40	10	91.38	83.45
		4C-2C	29	80	7	93.97	
		8C-4C	35	42	39	66.38	
		16C-8C	60	20	36	68.97	
		BL-16C	64	48	4	96.55	
**Mouse**	*in vivo*	*2C-MII*	74	32	10	91.38	82.06
		4C-2C	67	36	13	88.79	
		8C-4C	43	35	38	67.24	
		MO-8C	64	12	40	65.52	
		BL-MO	79	34	3	97.41	
	in vitro	*4C-2C*	67	46	3	97.41	81.89
		8C-4C	42	43	31	73.28	
		MO-8C	49	18	49	57.76	
		BL-MO	86	29	1	99.14	

Table S3. Overview of up and down differentially expressed genes (DEG) from Limma analysis of *in vivo versus in vitro* bovine and mouse pre-implantation development stages, related to Figure 6. Up: number of up-regulated genes; Down: number of down regulated genes.

Species	Stage	Up	Down	DEG %	Average DEG (%)
**Bovine**	*MII*	3317	1104	5.61	4.47
	2C	1557	1480	3.85	
	4C	1720	1021	3.48	
	8C	1786	1011	3.55	
	16C	2450	1136	4.55	
	BL	2879	1680	5.79	
**Mouse**	*2C*	2258	5467	11.77	9.58
	4C	1885	5126	10.68	
	8C	54	98	0.23	
	MO	1328	6934	12.58	
	BL	4126	4177	12.65	

Table S4. Overview of up and down differentially expressed pathways (DEP) from ROAST analysis comparing *in vivo* and *in vitro* bovine and mouse pre-implantation development stages (one-sided directional p-value < 0.05); related to figure 7. Up: number of up-regulated pathways; Down: number of down regulated pathways; NS: number of non-significant pathways.

Species	Stage	Up	Down	NS	DEP (%)	Average DEP (%)
**Bovine**	*MII*	51	51	14	87.93	85.35
	2C	37	49	30	74.14	
	4C	75	22	19	83.62	
	8C	67	29	20	82.76	
	16C	66	38	12	89.66	
	BL	77	32	7	93.97	
**Mouse**	*2C*	48	63	5	96.52	85.22
	4C	58	51	7	94.78	
	8C	18	35	63	46.09	
	MO	64	43	9	93.04	
	BL	51	59	6	95.65	

Table S5. Overview of culture conditions used for *in vitro* embryo production, of embryos which data were used in the present study; related to star methods.

Species	GEO entry	Culture media	Culture conditions	Reference
**Human**	GSE44183	Cleavage Medium (SAGE)	5% CO_2 and 5% O_2	Xue et al., 2013
	GSE71318	G1.3 and G2 Media (Vitrolife)	5% CO_2 and 5% O_2	Dang et al., 2016
**Bovine**	GSE44023	KSOMaa +5% serum	5% CO_2 and 5% O_2	Chitwood et al., 2013
	GSE121227	Not available	Not available	Unpublished
	GSE52415	SOF + 5% serum	5% CO_2 and 5% O_2	Graf et al., 2014
**Mouse**	GSE159484	KSOM	Not available	Unpublished

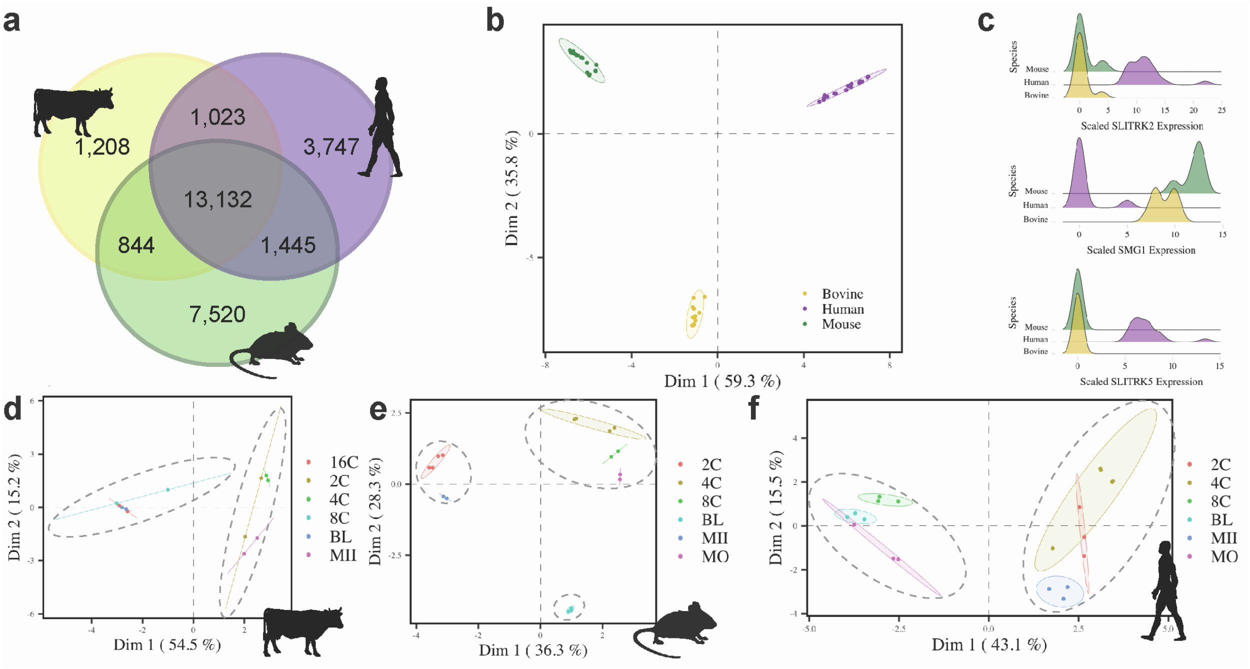

Figure S1. General analysis of *in vivo* bovine and mouse, and *in vitro* human mature oocytes (MII) and embryos (2C, 4C, 8C, 16C, MO and BL) gene expression; related to figures 1 and 2. The Venn diagram in **a** shows patterns of overlap in genes expressed between bovines, humans and mice. The scatter plot in **b** depicts the first two dimensions (Dim) of a principal component analysis (PCA) across the proximity matrix of a random forest model classifying species based on gene expression profiles. Ellipses depict the means and covariances of the first two dimensions of the PCA for each species. In **c**, density plots of the genes of primary importance for classifying inter-specific variation are shown. Principal component analyses (PCAs) showed clear patterns of (dis)similarities in gene expression across developmental stages for *in vivo* (bovine and mouse) and *in vitro* (human) oocyte and embryos These PCAs revealed two distinct clusters of *in vivo* bovine (**d**) and *in vitro* human (**f**) pre-implantation development: the first including the oocyte (MII), 2-cell (2C) and the 4-cell (4C) stages; and the second including the 8-cell (8C), the 16-cell (16C) or the morula (MO), and the blastocyst (BL) stages. In mice (**e**), in contrast, *in vivo* pre-implantation development presented three distinct clusters: the first included the MII and the 2C stages; the second the 4C, the 8C and the MO stages; and the third the BL stage.

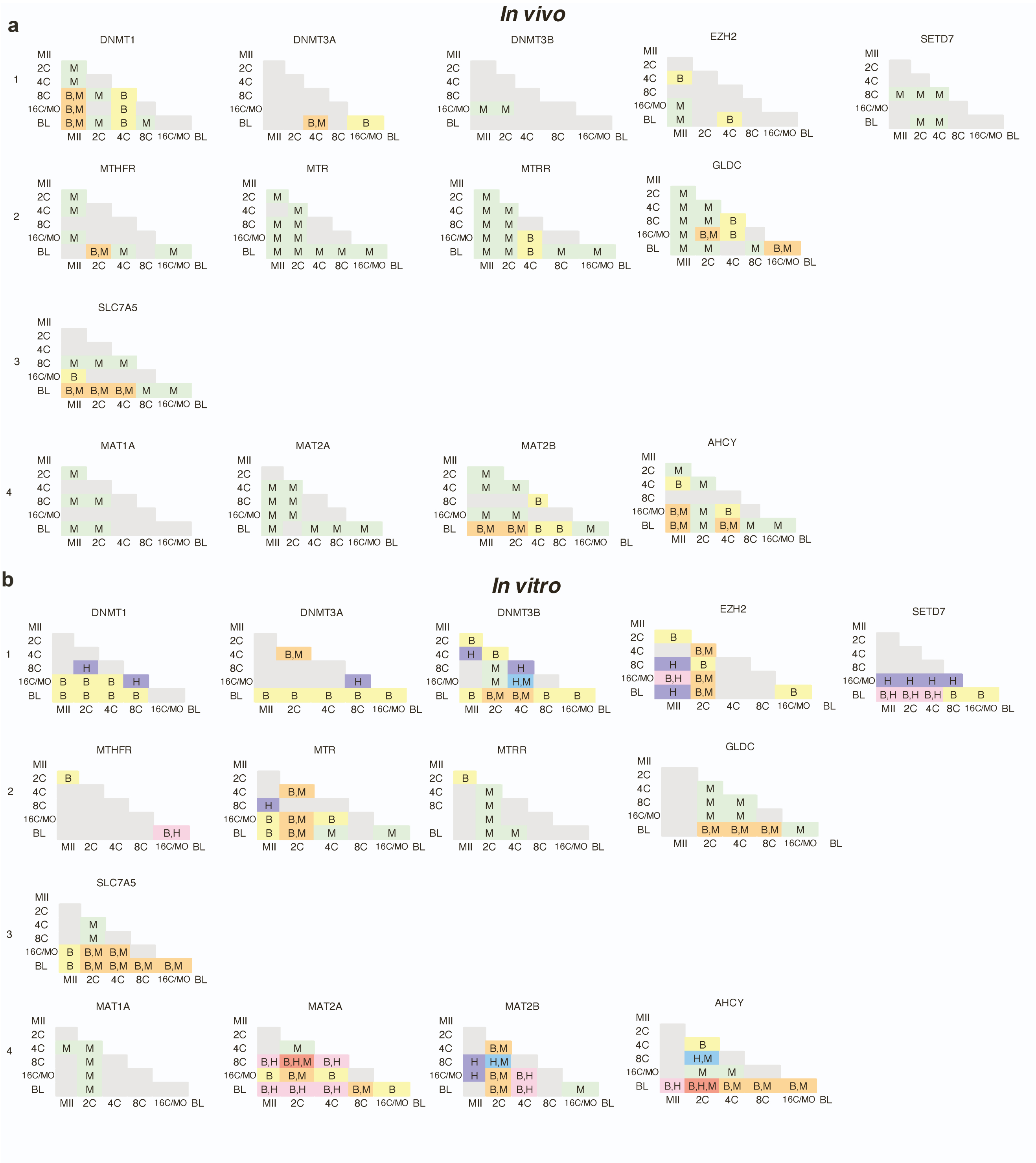

Figure S2. Significance of up and down regulated genes presented in Figure 5. Comparison of different stages for each species: human (H), bovine (B) and mouse (M) *in vivo* (a) and *in vitro* (b). Boxes marked with the species letter (H, B and/or M) indicates significant difference between stages for the corresponding specie(s) (Limma, adjusted p-value <0.05)

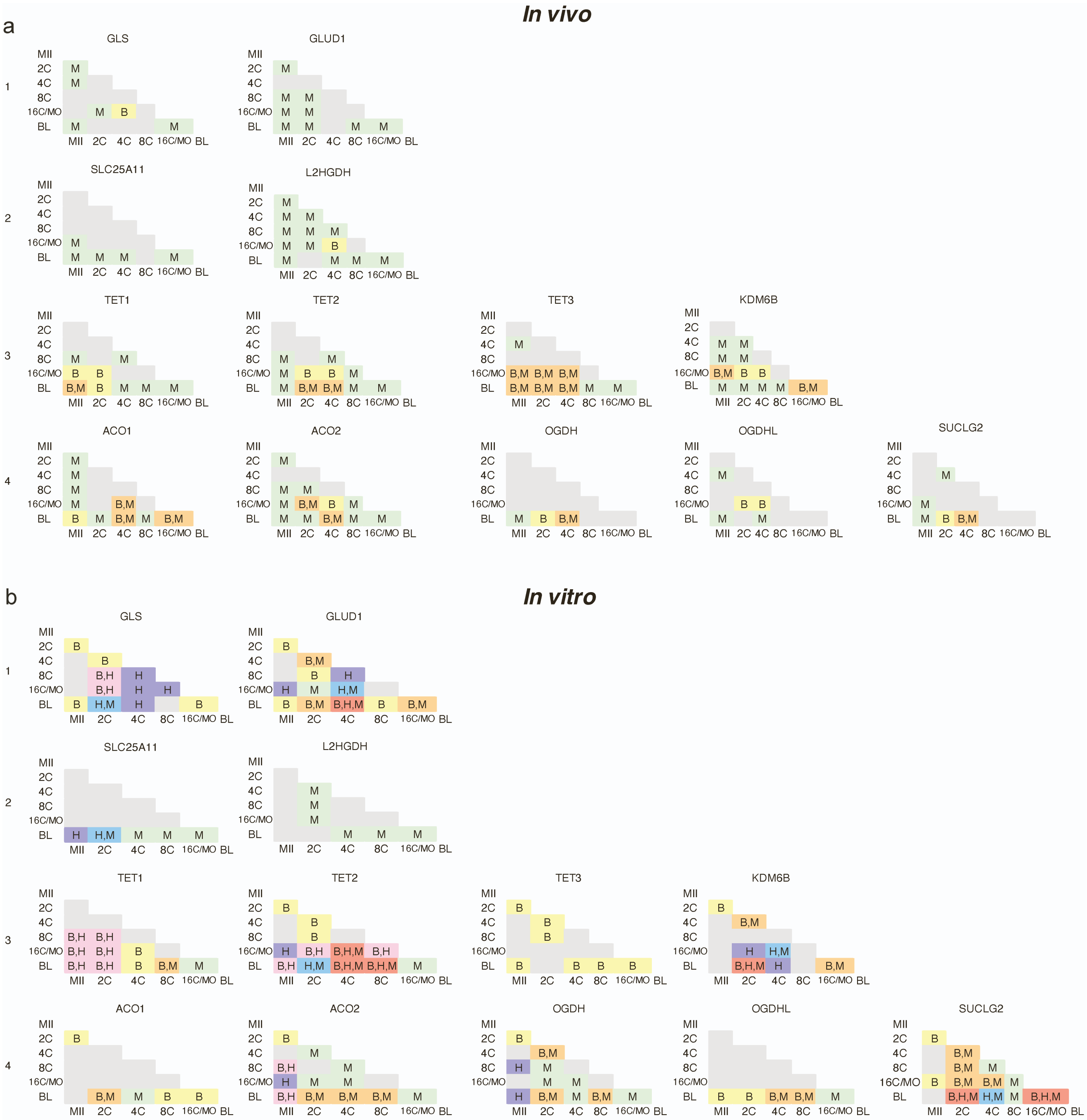

Figure S3. Significance of up and down regulated genes presented in Figure 6. Comparison of different stages for each species: human (H), bovine (B) and mouse (M) *in vivo* (a) and *in vitro* (b). Boxes marked with the species letter (H, B and/or M) indicates significant difference between stages for the corresponding specie(s) (Limma, adjusted p-value <0.05)

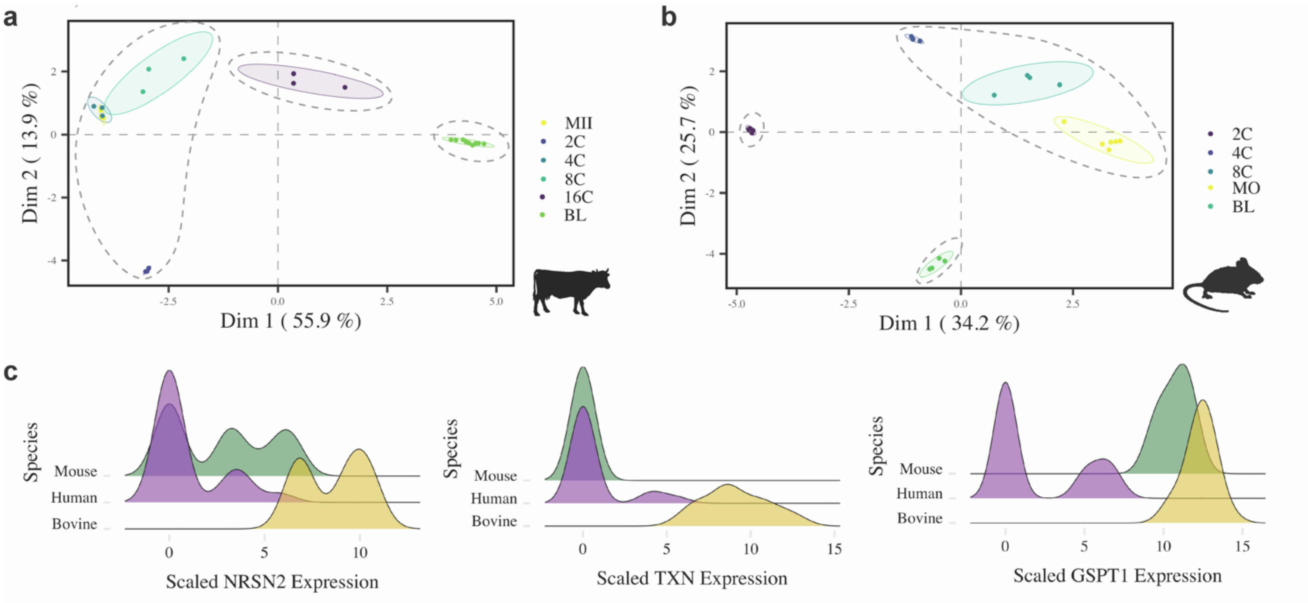

Figure S4. *In vitro* embryos differ from *in vivo* embryos; related to figure 6. General analysis of *in vitro* bovine and mouse mature oocyte (MII) and embryos (2C, 4C, 8C, 16C, MO and BL) gene expression. PCA of bovine (**a**) and mouse (**b**) *in vitro* developmental stages. In **c**, density plots of the genes of primary importance for classifying inter-species variation.

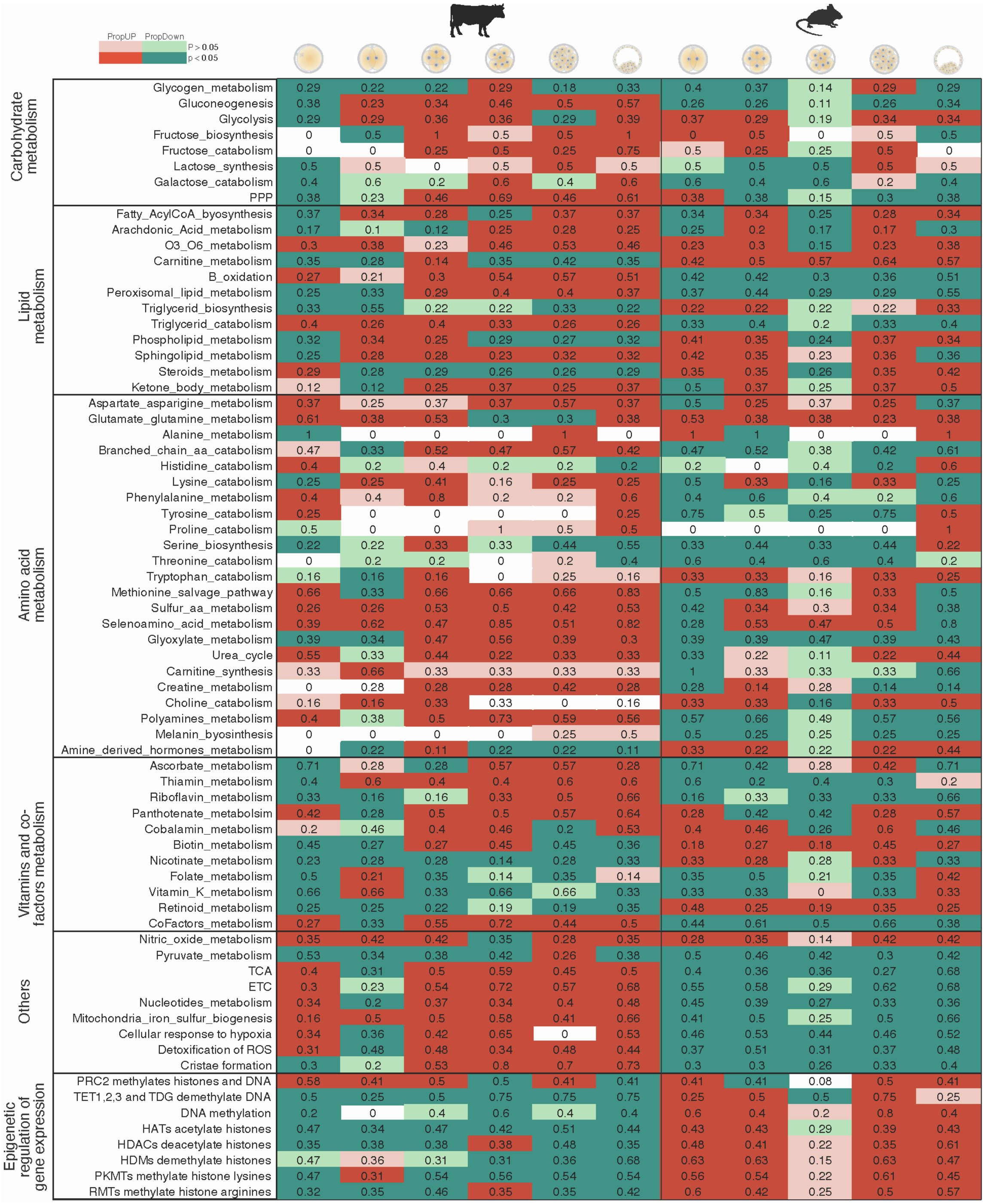

Figure S5. ***In vitro*** **metaboloepigenetic pathways are stage and species specific; related to Figure 7.** General analysis of mouse and bovine *in vitro* mature oocyte (MII) and embryos (2C, 4C, 8C, 16C, MO and BL) gene expression of metabolism and epigenetic genes, showing proportion of up (PropUp, red) and down (PropDown, green) regulated metabolic pathways (part of Reactome terms "Metabolism" and "Epigenetic regulation of gene expression") in 2C compared to MII, 4C compared to 2C, 8C compared to 4C, 16C compared to 8C and BL compared to 16C. Differences on PropUp and PropDown were calculated using the rotation gene set testing (ROAST), significant up and down regulated pathways presented the one-sided directional p-value < 0.05.

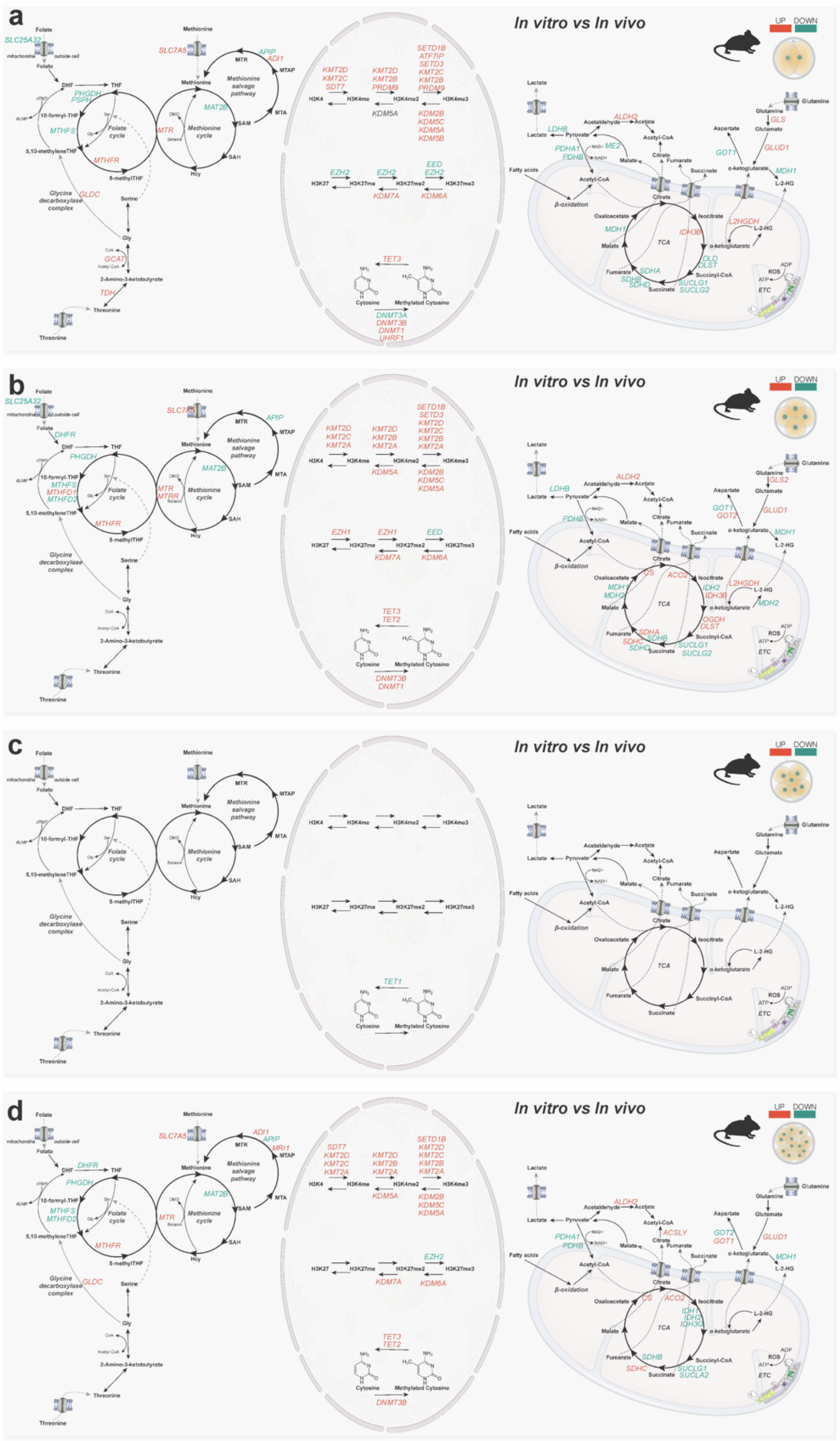

Figure S6. Metaboloepigenetic genes are differently expressed between mouse developmental stages; related to Figure 6. Metaboloepigenetic genes up (red) and down (green) regulated (adjusted p-value < 0.05) comparing *in vitro vs in vivo* mouse MII (**a**), 2C (**b**), 4C (**c**), 8C (**d**) and 16C (**e**).

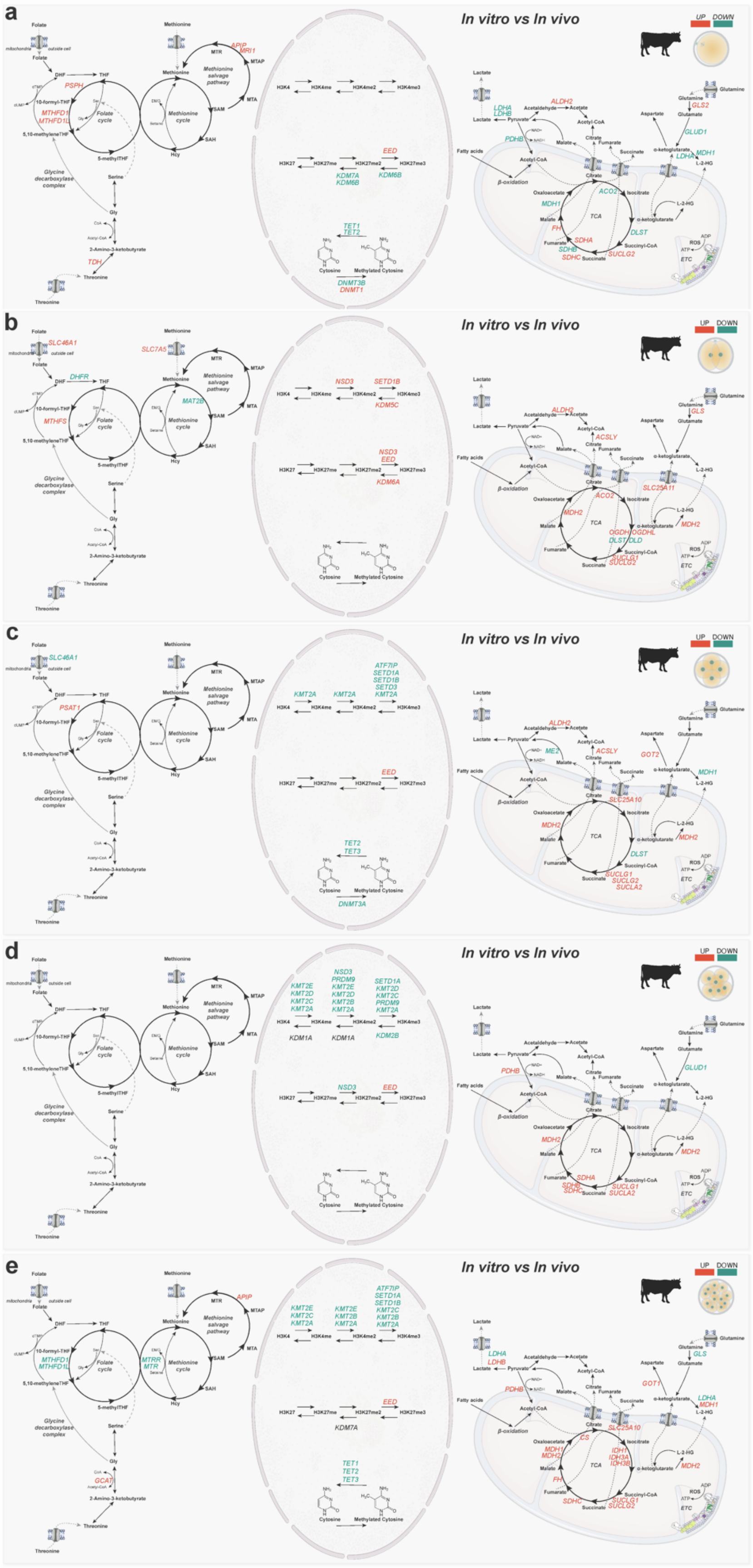

Figure S7. Metaboloepigenetic genes are differently expressed between bovine developmental stages; related to Figure 6. Metaboloepigenetic genes up (red) and down (green) regulated (adjusted p-value < 0.05) comparing *in vitro vs in vivo* bovine MII (**a**), 2C (**b**), 4C (**c**), 8C (**d**) and 16C (**e**).

Data S6: R script for reproducing the RNAseq and Pearson correlation analysis results presented in the main text; related to all figures, datasets and Star Methods.

31/01/2022

0.1 Data scaling

The first step was to transform the raw data so that they would be on the same scale. To render the data comparable across species and developmental stages, EdgeR transformed data were scaled using Probabilistic Quotient Normalisation (PQN; Dieterle et al. 2006), which calibrates individual gene expression profiles against the median profile. Notably, analyses on PQN transformed data have been shown to have low false-positive rates, and can accurately recover groups of interest without introducing artefactual differences (Noonan et al. 2018).

```
#Load in the packages
library(FactoMineR)
library(ggplot2)
library(ellipse)
library(randomForest)
library(caret)
library(ggridges)
library(viridis)
library(gridExtra)
library(limma)

#Load in the raw data
data <- read.csv("All_Merged_Selected.csv")
data <- data[1:13134,]
SPECIES <- data[1,-1]
GROUP <- data[2,-1]
NAMES <- data[-c(1:2),1]
data <- data.frame(t(data[-c(1:2),-1]))

data <- as.data.frame(do.call(cbind,lapply(1:ncol(data), function(t)
  data[,t] <- as.numeric(data[,t])
)))

colnames(data) <- NAMES

raw_data <- data

################################################################
# Normalisation by the methods of Brawand et al.
```

```r
##############################################################

RANGES <- apply(data, 1, IQR)
MED <- apply(data, 1, median)
MAX <- MED + RANGES/2
MIN <- MED - RANGES/2

#Which expression levels are within the min/max interquartile range
INSIDE <- list()
for(i in 1:nrow(data)){
  INSIDE_2 <- vector()
  for(j in 1:ncol(data)){
    INSIDE_2[j] <- (data[i,j] > MIN[i] && data[i,j] < MAX[i])
  } #Closes the loop over the columns
  INSIDE[[i]] <- INSIDE_2
} #Closes the loop over the rows

insides <- as.data.frame(do.call(rbind, INSIDE))

#Identify the 1000 most conserved genes
SUMS <- apply(insides, 2, sum)
names(SUMS) <- names(data)
SUMS <- sort(SUMS, decreasing = TRUE)
TOP <- data[,SUMS[1:1000]]

#Calculate the scaling factor based on the 1000 most conserved genes
FACTOR <- apply(TOP, 1, median)/mean(apply(TOP, 1, median))

#Transform the data based on the scaling factor and fix all unexpressed genes
to 0
data_scaled <- data
for(i in 1:nrow(data_scaled)){
  data_scaled[i,] <- data_scaled[i,]/FACTOR[i]
  data_scaled[i,][data_scaled[i,] == min(data_scaled[i,])] <- 0
}


##############################################################
# PQN Normalisation of the data
##############################################################

#Calculate the median of each gene's expression to generate a reference
sample
ref <- apply(data, 2, median)

for(i in 1:nrow(data)){
  QUOTIENTS <- data[i,]/ref
  m_j <- median(t(QUOTIENTS))
  data[i,] <- data[i,]/m_j
```

```r
  data[i,][data[i,] == min(data[i,])] <- 0
}


########################
# Generate the figure

par(mfrow = c(1,3))

#Visualise the raw data
for(i in 1:nrow(raw_data)){
  if(i == 1){plot(density(as.matrix(raw_data[i,])),
                  ylim = c(0,0.8),
                  col = viridis::viridis(nrow(raw_data))[i],
                  main = "Raw data",
                  xlab = "Raw expression values")
  } else {
    lines(density(as.matrix(raw_data[i,])),
          ylim = c(0,0.7),
          col = viridis::viridis(nrow(raw_data))[i])
  }
}

#Visualise the poorly normalised data
for(i in 1:nrow(data_scaled)){
  if(i == 1){plot(density(as.matrix(data[i,])),
                  ylim = c(0,0.8),
                  col = viridis::viridis(nrow(data))[i],
                  main = "Method of Brawand et al. 2011",
                  xlab = "Normalised expression values")
  } else {
    lines(density(as.matrix(data_scaled[i,])),
          ylim = c(0,0.7),
          col = viridis::viridis(nrow(data_scaled))[i])
  }
}

#Visualise the PQN normalised data
for(i in 1:nrow(data)){
  if(i == 1){plot(density(as.matrix(data[i,])),
                  ylim = c(0,0.8),
                  col = viridis::viridis(nrow(data))[i],
                  main = "Probabilistic Quotiont Normalisation",
                  xlab = "Normalised expression values")
  } else {
    lines(density(as.matrix(data[i,])),
          ylim = c(0,0.7),
          col = viridis::viridis(nrow(data))[i])
```

```
  }
}
```

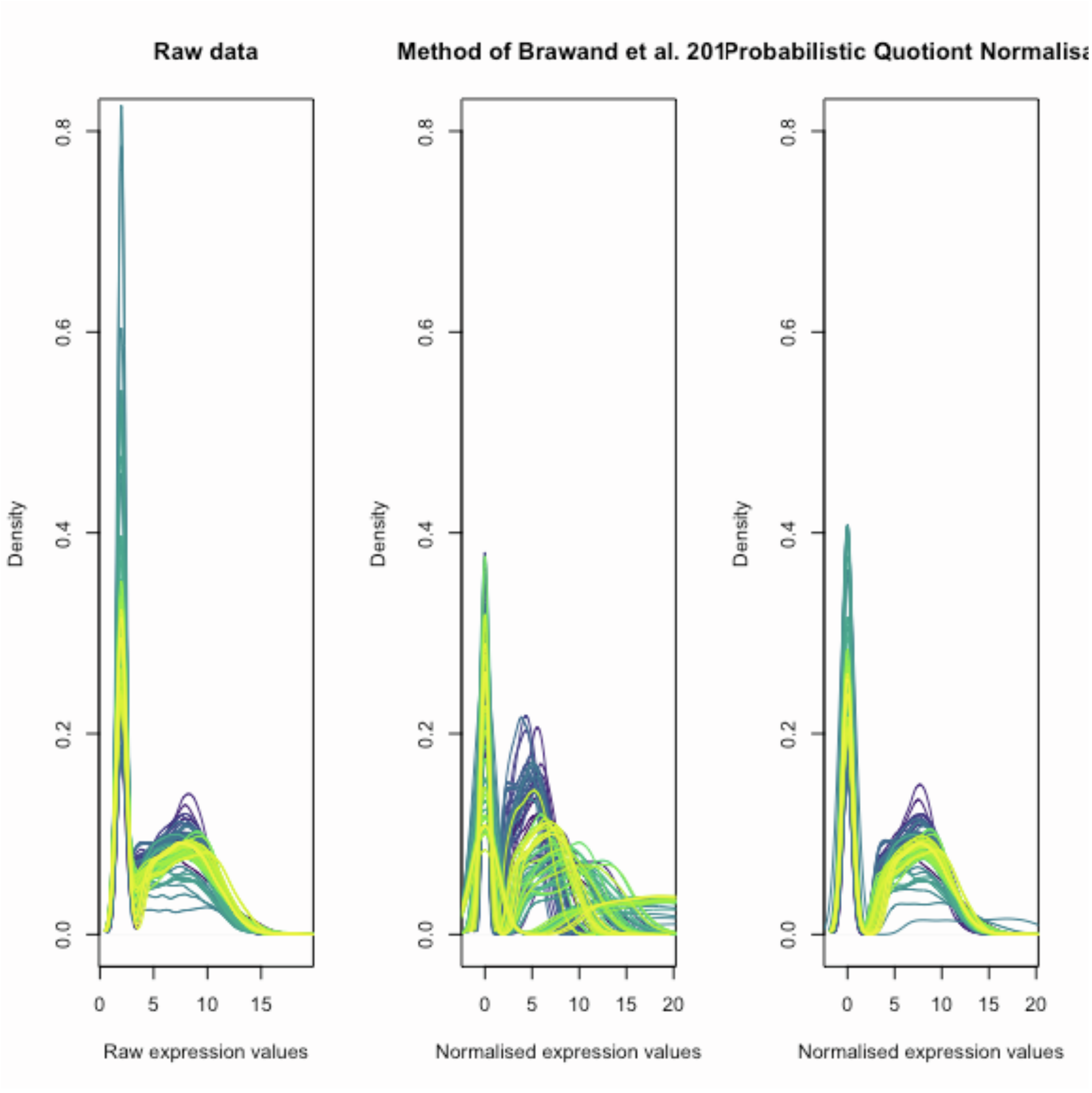


```
dev.off()

## null device
##           1
```

0.2 PCA on all data

First step of the analyses was to run a PCA on all of the data to predict species and plot the results to visualise any clustering that might be occurring.

```
#Load in the meta data
IDs <- na.omit(read.csv("all_extra.csv"))
```

```r
res.pca <- PCA(data, graph = FALSE) # Conduct a PCA on the data
PC1 <- res.pca$ind$coord[,1] #Store individual coordinates of PC1 as a vector
PC2 <- res.pca$ind$coord[,2] #Store individual coordinates of PC2 as a vector
PCs.ID <- data.frame(cbind(PC1,PC2)) #Bind the coordinates together as a
dataframe
PCs.ID$Species <- (IDs$Species) #Add in Species to the df

#Define axis labels based on % of data explained across each dimension of the
PCA
DIM_1 <- paste("PCA Dimension 1 (", round(res.pca$eig[1,2], 1), "%)")
DIM_2 <- paste("PCA Dimmension 2  (", round(res.pca$eig[2,2], 1), "%)")

#Draw ellipses around the cluster
centroids <- aggregate(cbind(PC1,PC2)~Species,PCs.ID,mean)
conf.rgn  <- do.call(rbind,lapply(unique(PCs.ID$Species),function(t)
  data.frame(Species=as.character(t),
             ellipse(cov(PCs.ID[PCs.ID$Species==t,1:2]),

centre=as.matrix(centroids[which(unique(PCs.ID$Species)==t),2:3]),
                     level=0.95),
             stringsAsFactors=FALSE)))

#Then make the figure
ggplot(PCs.ID, aes(x=PC1, y=PC2, color = Species), guide = FALSE) +
  geom_path(data=conf.rgn, alpha=0.2, size = 0, show.legend = FALSE) +
  geom_polygon(data=conf.rgn,
               aes(fill = Species),
               alpha=0.1, size = 0.1,
               show.legend = FALSE) +
  geom_hline(aes(yintercept=0), linetype="dashed", lwd = 0.1) +
  geom_vline(aes(xintercept=0), linetype="dashed", lwd = 0.1) +
  theme_bw() +
  geom_point(size=0.4, aes(color = Species)) +
  scale_color_manual(labels=c("Bovine", "Human", "Mouse"),
                     values = c("#e6c141", "#8a3bb8", "#3c7a47")) +
  scale_fill_manual(labels=c("Bovine", "Human", "Mouse"),
                    values = c("#e6c141", "#8a3bb8", "#3c7a47"),
                    guide = FALSE) +
  ylab(DIM_2) +
  xlab(DIM_1) +
  theme(panel.grid.major = element_blank(),
        panel.border = element_rect(colour = "black", size=1),
        panel.grid.minor = element_blank(),
        axis.title.x  = element_text(size=10, family = "serif"),
        axis.title.y  = element_text(size=10, family = "serif"),
        plot.title = element_text(size=8, hjust = 0, family = "serif"),
        axis.text.y  = element_text(size=5, family = "serif"),
        axis.text.x  = element_text(size=5, family = "serif"),
```

```
        legend.position=c(0.15,0.9),
        legend.background = element_blank(),
        legend.title = element_blank(),
        legend.text = element_text(size=8, family = "serif"),
        legend.key.size = unit(0.3, "cm"),
        legend.key = element_blank())
```

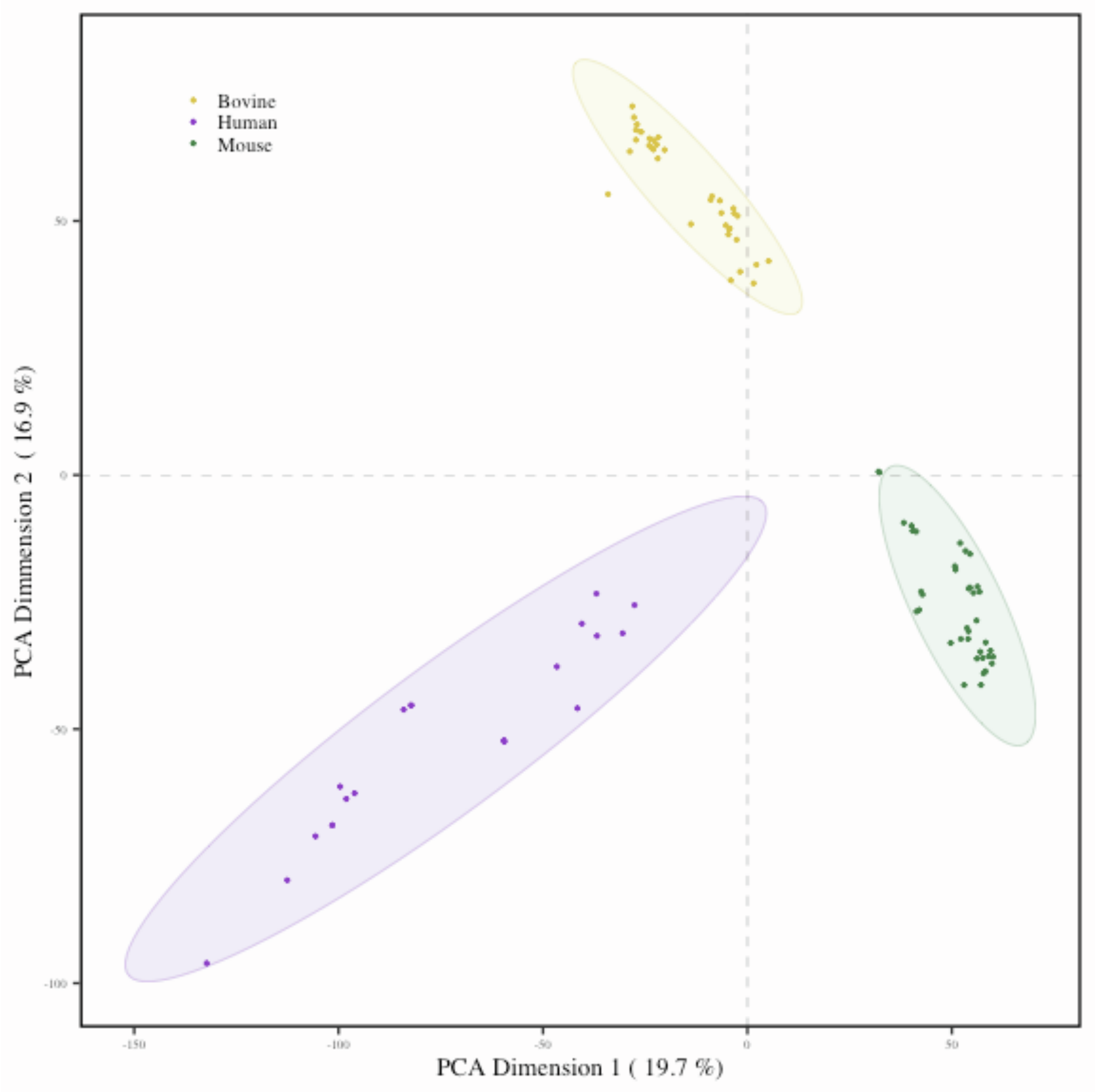


```
ggsave(file="Figures/Species_PCAF.png",
       width = 3.23,
       height=3,
       units = "in",
       dpi = 600)
```

0.3 Random Forest Classification

After this initial data visualisation, a random forest (RF) model (Ho 1995) was used to classify intra- and inter-species gene expression profiles according to developmental stages and collection method (in vivo or in vitro), with scaled gene expression values as the prediction variables. This allowed us to determine how well information contained within the expression data could be used to predict classes of interest. These analyses were conducted using the R package randomForest (RColorBrewer and Liaw, 2018). We chose RF modeling as it does not require any parameter reduction prior to analysis (Cutler et al. 2007), and has been shown to provide reliable results for biomarker identification (Chen et al. 2017; Noonan et al. 2018). Identification of genes important for classifying groups of interest in each RF model was carried out using RF variable importance values.

0.3.1 Classify species with in vivo data

Our first RF model aimed to classify species based on the data collected *in vivo*. Because data from humans were not collected *in vivo*, the *in vitro* data for humans were used here for comparison.

```
#Create a dataset that contains only the in vivo data (except for humans)
Species_Data <- data
Species_Data$Species <- as.factor(IDs$Species)
names(Species_Data) <- make.names(names(Species_Data))
Species_Data <- Species_Data[-which(IDs$Collection == "vitro" & IDs$Species
== "mouse"),]
Species_Data <- Species_Data[-which(IDs$Collection == "vitro" & IDs$Species
== "cow"),]

#Run the random forest model identifying species
Species.mod <- randomForest(y = Species_Data$Species,
                            x = Species_Data[, colnames(Species_Data) !=
"Species"],
                            mtry =  5,
                            ntree= 20000,
                            importance=TRUE,
                            proximity = TRUE,
                            keep.forest=TRUE,
                            replace = TRUE)

Species.mod

##
## Call:
##  randomForest(x = Species_Data[, colnames(Species_Data) != "Species"],
y = Species_Data$Species, ntree = 20000, mtry = 5, replace = TRUE,
importance = TRUE, proximity = TRUE, keep.forest = TRUE)
##                Type of random forest: classification
##                      Number of trees: 20000
## No. of variables tried at each split: 5
```

```
##
##         OOB estimate of  error rate: 0%
## Confusion matrix:
##        cow human mouse class.error
## cow     12     0     0           0
## human    0    20     0           0
## mouse    0     0    17           0
```

```
varImpPlot(Species.mod, type=1, scale = FALSE)
```

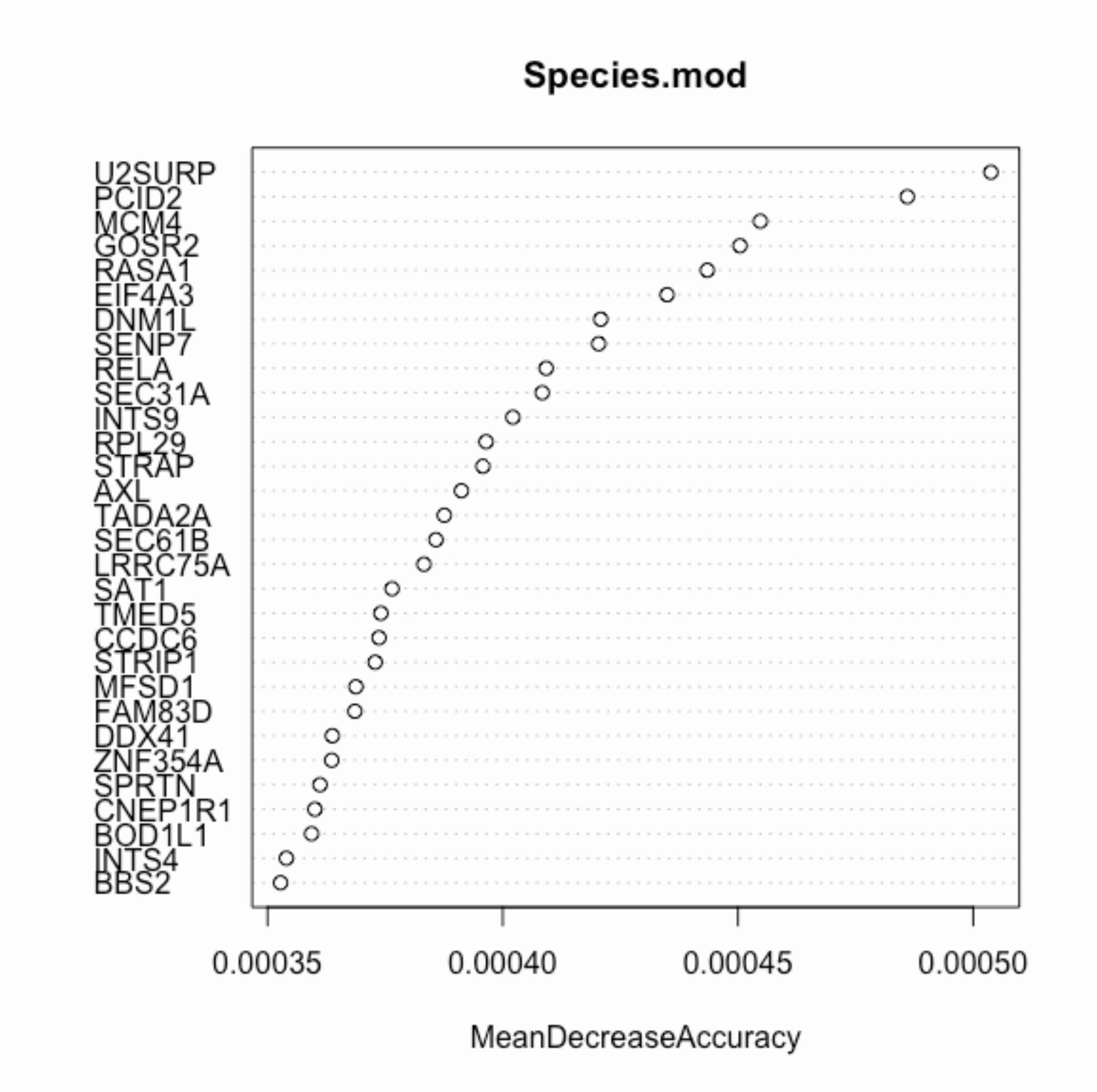


```
#Get top 20 genes
PRIME_GENES <-
row.names(Species.mod$importance)[order(Species.mod$importance[,"MeanDecrease
Accuracy"],
                                                                      decreasing =
TRUE)][1:20]
PRIME_GENES
```

```
##  [1] "U2SURP"  "PCID2"   "MCM4"    "GOSR2"   "RASA1"   "EIF4A3"  "DNM1L"
##  [8] "SENP7"   "RELA"    "SEC31A"  "INTS9"   "RPL29"   "STRAP"   "AXL"
## [15] "TADA2A"  "SEC61B"  "LRRC75A" "SAT1"    "TMED5"   "CCDC6"
```

```
res.pca <- PCA(Species.mod$proximity, graph = FALSE) # Conduct a PCA on the
proximity matrix
PC1 <- res.pca$ind$coord[,1] #Store individual coordinates of PC1 as a vector
PC2 <- res.pca$ind$coord[,2] #Store individual coordinates of PC2 as a vector
PCs.ID <- data.frame(cbind(PC1,PC2)) #Bind the coordinates together as a
dataframe
PCs.ID$Subject <- Species_Data$Species #Add in species to the df

#Define axis labels based on % of data explained across each dimension of the
PCA
DIM_1 <- paste("Dim 1 (", round(res.pca$eig[1,2], 1), "%)")
DIM_2 <- paste("Dim 2 (", round(res.pca$eig[2,2], 1), "%)")

#Draw ellipses around the clusters and highlighting the elipsoids rather than
the data points
centroids <- aggregate(cbind(PC1,PC2)~Subject,PCs.ID,mean)
conf.rgn  <- do.call(rbind,lapply(unique(PCs.ID$Subject),function(t)
  data.frame(Subject=as.character(t),
             ellipse(cov(PCs.ID[PCs.ID$Subject==t,1:2]),
                     centre=as.matrix(centroids[t,2:3]),
                     level=0.95),
             stringsAsFactors=FALSE)))

#Then make the figure
PCA_FIG_Species <-
  ggplot(PCs.ID, aes(x=PC1, y=PC2, color = Subject)) +
  geom_hline(aes(yintercept=0), linetype="dashed", lwd = 0.1) +
  geom_vline(aes(xintercept=0), linetype="dashed", lwd = 0.1) +
  geom_path(data=conf.rgn, alpha=0.2, size = 0) +
  geom_polygon(data=conf.rgn,
               aes(fill = Subject),
               alpha=0.1,
               size = 0.1,
               show.legend = FALSE) +
  geom_point(size=0.4) +
  theme_bw() +
  ylab(DIM_2) +
  xlab(DIM_1) +
  geom_point(size=0.4, aes(color = Subject)) +
  scale_color_manual(labels=c("Bovine", "Human", "Mouse"),
                     values = c("#e6c141", "#8a3bb8", "#3c7a47")) +
  scale_fill_manual(labels=c("Bovine", "Human", "Mouse"),
                    values = c("#e6c141", "#8a3bb8", "#3c7a47"),
                    guide = FALSE) +
  ylab(DIM_2) +
  xlab(DIM_1) +
```

```
  theme(panel.grid.major = element_blank(),
        panel.border = element_rect(colour = "black", size=1),
        panel.grid.minor = element_blank(),
        axis.title.x  = element_text(size=10, family = "serif"),
        axis.title.y  = element_text(size=10, family = "serif"),
        plot.title = element_text(size=8, hjust = 0, family = "serif"),
        axis.text.y  = element_text(size=5, family = "serif"),
        axis.text.x  = element_text(size=5, family = "serif"),
        legend.position=c(0.8,0.15),
        legend.background = element_blank(),
        legend.title = element_blank(),
        legend.text = element_text(size=8, family = "serif"),
        legend.key.size = unit(0.3, "cm"),
        legend.key = element_blank())

ggsave(PCA_FIG_Species,
       file="Figures/Species_PCA_RF_Vivo.png",
       width = 3.23,
       height=3,
       units = "in",
       dpi = 600)

#Figure of the primary genes
X_LAB <- paste("Scaled", PRIME_GENES[1], "Expression")

a <-
  ggplot(Species_Data, aes(x=Species_Data[,PRIME_GENES[1]], y=Species, fill =
Species)) +
  geom_density_ridges(scale = 5, alpha=0.6, size = 0.2) +
  theme_ridges() +
  scale_fill_manual(labels=c("Bovine", "Human", "Mouse"),
                    values = c("#e6c141", "#8a3bb8", "#3c7a47")) +
  scale_y_discrete(expand = c(0.1, 0), labels=c("Bovine", "Human", "Mouse"))
+
  labs(x=X_LAB, y="Species")+
  ggtitle("a)")+
  theme(plot.title = element_text(hjust = 0, size = 10, family = "serif"),
        panel.grid.major = element_blank(),
        panel.border = element_rect(colour = "black", size=1),
        panel.grid.minor = element_blank(),
        axis.title.x  = element_text(hjust = 0.5, size=10, family = "serif"),
        axis.title.y  = element_text(hjust = 0.5, size=10, family = "serif"),
        axis.text.y  = element_text(size=8, family = "serif"),
        axis.text.x  = element_text(size=8, family = "serif"),
        legend.position=c("none"))

X_LAB <- paste("Scaled", PRIME_GENES[2], "Expression")
b <-
  ggplot(Species_Data, aes(x=Species_Data[,PRIME_GENES[2]], y=Species, fill =
```

```
Species)) +
  geom_density_ridges(scale = 5, alpha=0.6, size = 0.2) +
  theme_ridges() +
  scale_fill_manual(labels=c("Bovine", "Human", "Mouse"),
                    values = c("#e6c141", "#8a3bb8", "#3c7a47")) +
  scale_y_discrete(expand = c(0.1, 0), labels=c("Bovine", "Human", "Mouse"))
+
  labs(x=X_LAB, y="Species")+
  ggtitle("b)")+
  theme(plot.title = element_text(hjust = 0, size = 10, family = "serif"),
        panel.grid.major = element_blank(),
        panel.border = element_rect(colour = "black", size=1),
        panel.grid.minor = element_blank(),
        axis.title.x  = element_text(hjust = 0.5, size=10, family = "serif"),
        axis.title.y  = element_text(hjust = 0.5, size=10, family = "serif"),
        axis.text.y  = element_text(size=8, family = "serif"),
        axis.text.x  = element_text(size=8, family = "serif"),
        legend.position=c("none"))

X_LAB <- paste("Scaled", PRIME_GENES[3], "Expression")
c <-
  ggplot(Species_Data, aes(x=Species_Data[,PRIME_GENES[3]], y=Species, fill =
Species))+
  geom_density_ridges(scale = 5, alpha=0.6, size = 0.2) +
  theme_ridges() +
  scale_fill_manual(labels=c("Bovine", "Human", "Mouse"),
                    values = c("#e6c141", "#8a3bb8", "#3c7a47")) +
  scale_y_discrete(expand = c(0.1, 0), labels=c("Bovine", "Human", "Mouse"))
+
  labs(x=X_LAB, y="Species")+
  ggtitle("c)")+
  theme(plot.title = element_text(hjust = 0, size = 10, family = "serif"),
        panel.grid.major = element_blank(),
        panel.border = element_rect(colour = "black", size=1),
        panel.grid.minor = element_blank(),
        axis.title.x  = element_text(hjust = 0.5, size=10, family = "serif"),
        axis.title.y  = element_text(hjust = 0.5, size=10, family = "serif"),
        axis.text.y  = element_text(size=8, family = "serif"),
        axis.text.x  = element_text(size=8, family = "serif"),
        legend.position=c("none"))

FIG <- arrangeGrob(a, b, c, ncol = 1)

grid.arrange(a, b, c, ncol = 1)
```

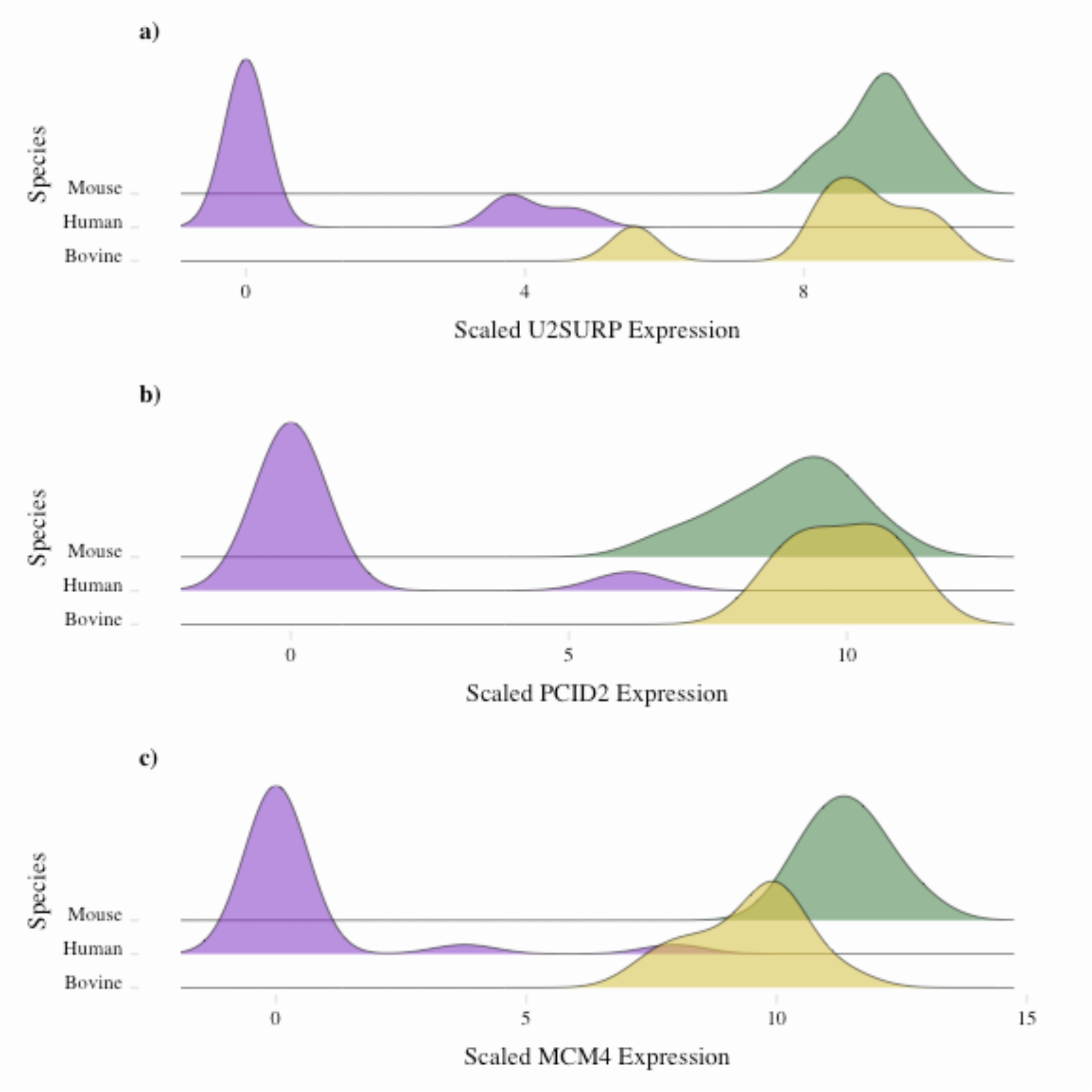


```
ggsave(FIG,
       file="Figures/Species_Density_Plot_Vivo.png",
       width = 3.23,
       height=6,
       units = "in",
       dpi = 600)
```

0.3.2 Classify species with in vitro data

Our next RF model aimed to classify species based on the data collected *in vitro*.

```
#Create a dataset that contains on the in vitro data
Species_Data <- data
Species_Data$Species <- as.factor(IDs$Species)
names(Species_Data) <- make.names(names(Species_Data))
Species_Data <- Species_Data[-which(IDs$Collection == "vivo" & IDs$Species ==
"mouse"),]
```

```
Species_Data <- Species_Data[-which(IDs$Collection == "vivo" & IDs$Species ==
"cow"),]

#Run the random forest model identifying species
Species.mod <- randomForest(y = Species_Data$Species,
                            x = Species_Data[, colnames(Species_Data) !=
"Species"],
                            mtry =  5,
                            ntree= 20000,
                            importance=TRUE,
                            proximity = TRUE,
                            keep.forest=TRUE,
                            replace = TRUE)
Species.mod

##
## Call:
##  randomForest(x = Species_Data[, colnames(Species_Data) != "Species"],
y = Species_Data$Species, ntree = 20000, mtry = 5, replace = TRUE,
importance = TRUE, proximity = TRUE, keep.forest = TRUE)
##                Type of random forest: classification
##                      Number of trees: 20000
## No. of variables tried at each split: 5
##
##         OOB estimate of  error rate: 0%
## Confusion matrix:
##       cow human mouse class.error
## cow    24     0     0           0
## human   0    20     0           0
## mouse   0     0    22           0

varImpPlot(Species.mod, type=1, scale = FALSE)
```

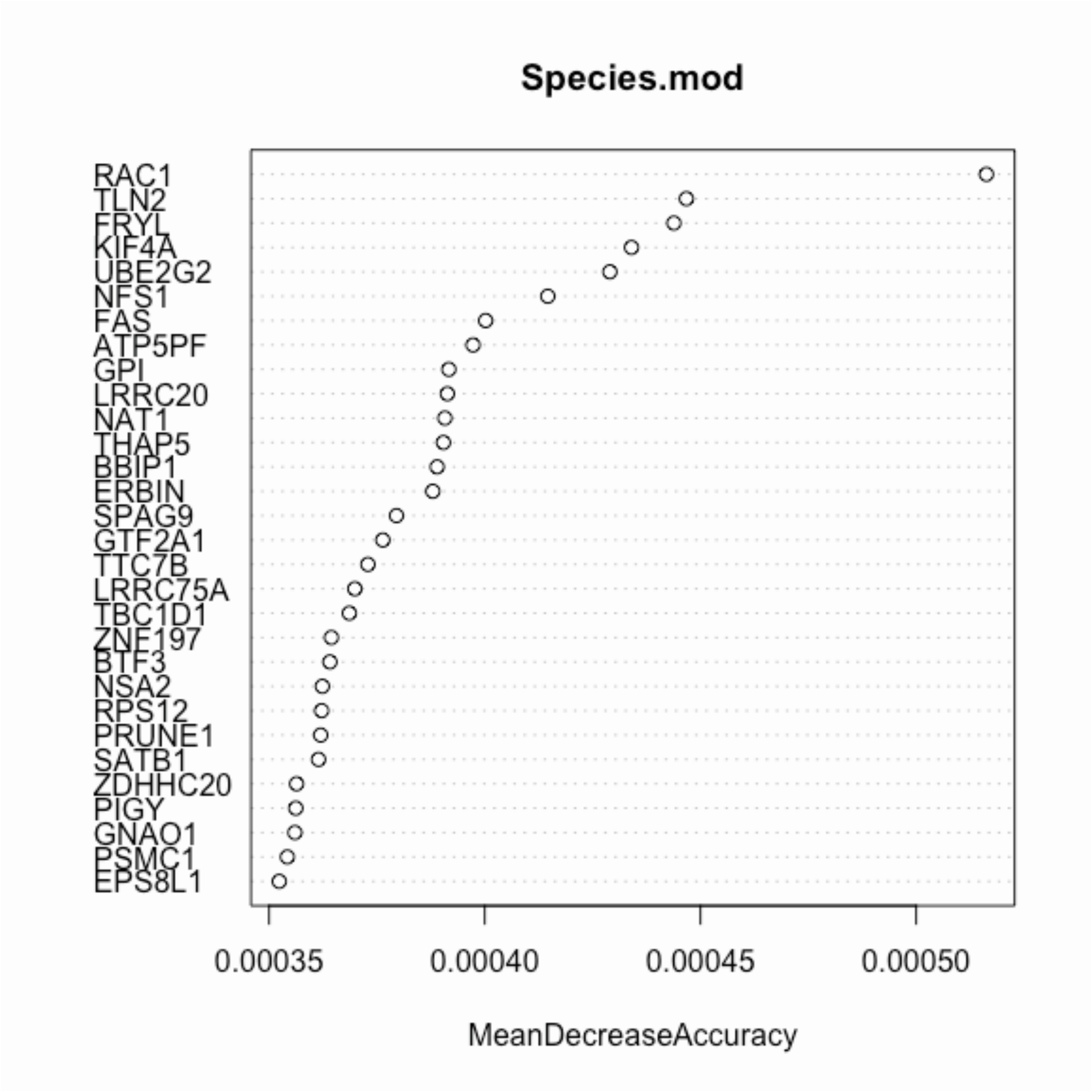


```
#Get top 20 genes
PRIME_GENES <-
row.names(Species.mod$importance)[order(Species.mod$importance[,"MeanDecrease
Accuracy"],
                                                                    decreasing =
TRUE)][1:20]
PRIME_GENES

##  [1] "RAC1"    "TLN2"    "FRYL"    "KIF4A"   "UBE2G2"  "NFS1"    "FAS"
##  [8] "ATP5PF"  "GPI"     "LRRC20"  "NAT1"    "THAP5"   "BBIP1"   "ERBIN"
## [15] "SPAG9"   "GTF2A1"  "TTC7B"   "LRRC75A" "TBC1D1"  "ZNF197"

res.pca <- PCA(Species.mod$proximity, graph = FALSE) # Conduct a PCA on the
proximity matrix
PC1 <- res.pca$ind$coord[,1] #Store individual coordinates of PC1 as a vector
PC2 <- res.pca$ind$coord[,2] #Store individual coordinates of PC2 as a vector
PCs.ID <- data.frame(cbind(PC1,PC2)) #Bind the coordinates together as a
```

```
dataframe
PCs.ID$Subject <- Species_Data$Species #Add in species to the df

#Define axis labels based on % of data explained across each dimension of the
PCA
DIM_1 <- paste("Dim 1 (", round(res.pca$eig[1,2], 1), "%)")
DIM_2 <- paste("Dim 2 (", round(res.pca$eig[2,2], 1), "%)")

#Draw ellipses around the clusters and highlighting the elipsoids rather than
the data points
centroids <- aggregate(cbind(PC1,PC2)~Subject,PCs.ID,mean)
conf.rgn  <- do.call(rbind,lapply(unique(PCs.ID$Subject),function(t)
  data.frame(Subject=as.character(t),
             ellipse(cov(PCs.ID[PCs.ID$Subject==t,1:2]),
                     centre=as.matrix(centroids[t,2:3]),
                     level=0.95),
             stringsAsFactors=FALSE)))



#Then make the figure
ggplot(PCs.ID, aes(x=PC1, y=PC2, color = Subject)) +
  geom_hline(aes(yintercept=0), linetype="dashed", lwd = 0.1) +
  geom_vline(aes(xintercept=0), linetype="dashed", lwd = 0.1) +
  geom_path(data=conf.rgn, alpha=0.2, size = 0) +
  geom_polygon(data=conf.rgn,
               aes(fill = Subject),
               alpha=0.1,
               size = 0.1,
               show.legend = FALSE) +
  geom_point(size=0.4) +
  theme_bw() +
  ylab(DIM_2) +
  xlab(DIM_1) +
  geom_point(size=0.4, aes(color = Subject)) +
  scale_color_manual(labels=c("Bovine", "Human", "Mouse"),
                     values = c("#e6c141", "#8a3bb8", "#3c7a47")) +
  scale_fill_manual(labels=c("Bovine", "Human", "Mouse"),
                    values = c("#e6c141", "#8a3bb8", "#3c7a47"),
                    guide = FALSE) +
  ylab(DIM_2) +
  xlab(DIM_1) +
  theme(panel.grid.major = element_blank(),
        panel.border = element_rect(colour = "black", size=1),
        panel.grid.minor = element_blank(),
        axis.title.x  = element_text(size=10, family = "serif"),
        axis.title.y  = element_text(size=10, family = "serif"),
        plot.title = element_text(size=8, hjust = 0, family = "serif"),
        axis.text.y  = element_text(size=5, family = "serif"),
        axis.text.x  = element_text(size=5, family = "serif"),
```

```
        legend.position=c(0.8,0.15),
        legend.background = element_blank(),
        legend.title = element_blank(),
        legend.text = element_text(size=8, family = "serif"),
        legend.key.size = unit(0.3, "cm"),
        legend.key = element_blank())
```

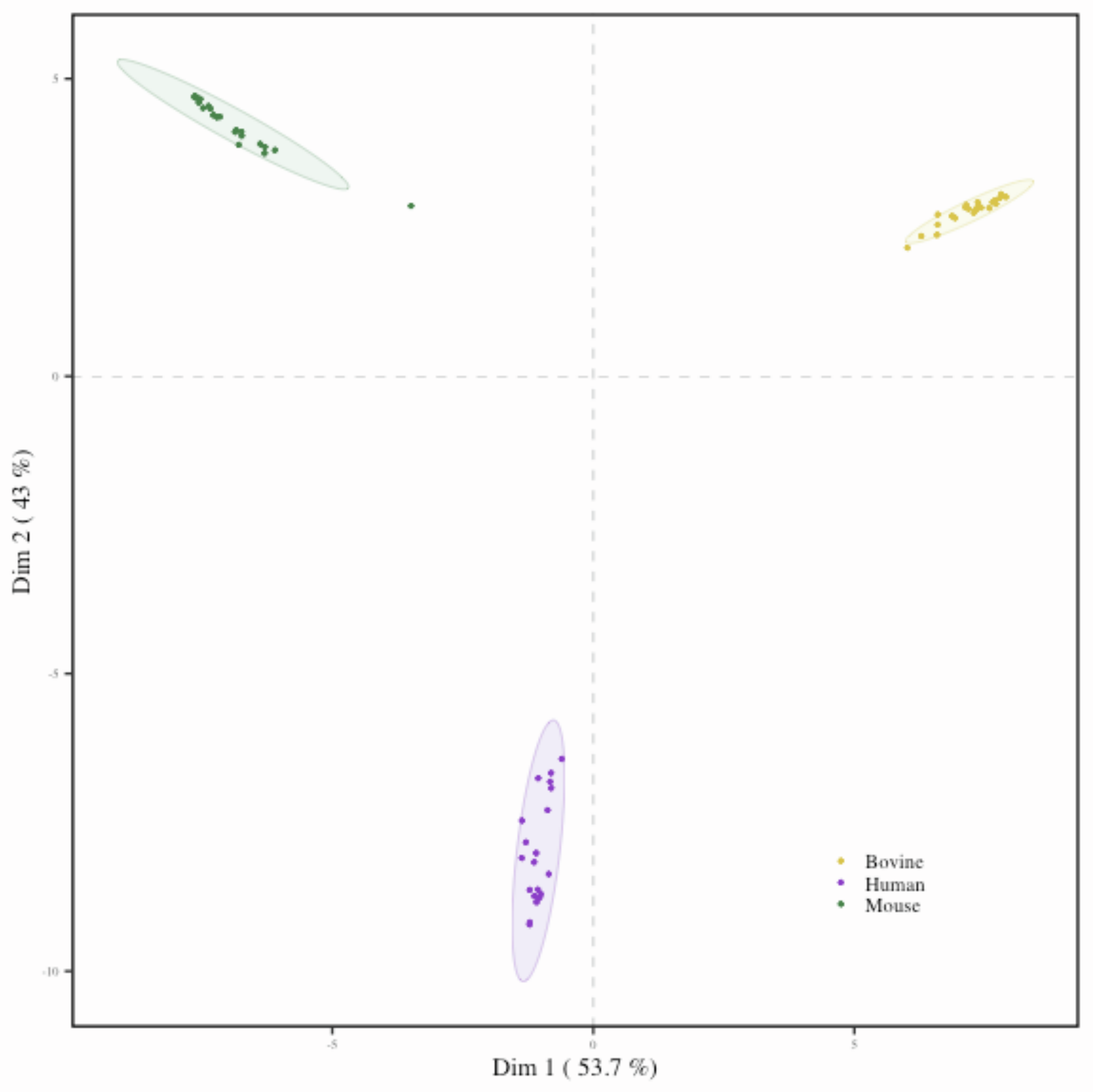


```
ggsave(file="Figures/Species_PCA_RF_Vitro.png",
       width = 3.23,
       height=3,
       units = "in",
       dpi = 600)

X_LAB <- paste("Scaled", PRIME_GENES[1], "Expression")
a <-
  ggplot(Species_Data, aes(x=Species_Data[,PRIME_GENES[1]], y=Species, fill =
Species))+
```

```
  geom_density_ridges(scale = 5, alpha=0.6, size = 0.2) +
  theme_ridges() +
  scale_fill_manual(labels=c("Bovine", "Human", "Mouse"),
                    values = c("#e6c141", "#8a3bb8", "#3c7a47")) +
  scale_y_discrete(expand = c(0.1, 0), labels=c("Bovine", "Human", "Mouse"))
+
  labs(x=X_LAB, y="Species")+
  ggtitle("a)")+
  theme(plot.title = element_text(hjust = 0, size = 10, family = "serif"),
        panel.grid.major = element_blank(),
        panel.border = element_rect(colour = "black", size=1),
        panel.grid.minor = element_blank(),
        axis.title.x  = element_text(hjust = 0.5, size=10, family = "serif"),
        axis.title.y  = element_text(hjust = 0.5, size=10, family = "serif"),
        axis.text.y  = element_text(size=8, family = "serif"),
        axis.text.x  = element_text(size=8, family = "serif"),
        legend.position=c("none"))

X_LAB <- paste("Scaled", PRIME_GENES[2], "Expression")
b <-
  ggplot(Species_Data, aes(x=Species_Data[,PRIME_GENES[2]], y=Species, fill =
Species))+
  geom_density_ridges(scale = 5, alpha=0.6, size = 0.2) +
  theme_ridges() +
  scale_fill_manual(labels=c("Bovine", "Human", "Mouse"),
                    values = c("#e6c141", "#8a3bb8", "#3c7a47")) +
  scale_y_discrete(expand = c(0.1, 0), labels=c("Bovine", "Human", "Mouse"))
+
  labs(x=X_LAB, y="Species")+
  ggtitle("b)")+
  theme(plot.title = element_text(hjust = 0, size = 10, family = "serif"),
        panel.grid.major = element_blank(),
        panel.border = element_rect(colour = "black", size=1),
        panel.grid.minor = element_blank(),
        axis.title.x  = element_text(hjust = 0.5, size=10, family = "serif"),
        axis.title.y  = element_text(hjust = 0.5, size=10, family = "serif"),
        axis.text.y  = element_text(size=8, family = "serif"),
        axis.text.x  = element_text(size=8, family = "serif"),
        legend.position=c("none"))

X_LAB <- paste("Scaled", PRIME_GENES[3], "Expression")
c <-
  ggplot(Species_Data, aes(x=Species_Data[,PRIME_GENES[3]], y=Species, fill =
Species))+
  geom_density_ridges(scale = 5, alpha=0.6, size = 0.2) +
  theme_ridges() +
  scale_fill_manual(labels=c("Bovine", "Human", "Mouse"),
                    values = c("#e6c141", "#8a3bb8", "#3c7a47")) +
  scale_y_discrete(expand = c(0.1, 0), labels=c("Bovine", "Human", "Mouse"))
+
```

```
  labs(x=X_LAB, y="Species")+
  ggtitle("c)")+
  theme(plot.title = element_text(hjust = 0, size = 10, family = "serif"),
        panel.grid.major = element_blank(),
        panel.border = element_rect(colour = "black", size=1),
        panel.grid.minor = element_blank(),
        axis.title.x  = element_text(hjust = 0.5, size=10, family = "serif"),
        axis.title.y  = element_text(hjust = 0.5, size=10, family = "serif"),
        axis.text.y  = element_text(size=8, family = "serif"),
        axis.text.x  = element_text(size=8, family = "serif"),
        legend.position=c("none"))

FIG <- arrangeGrob(a, b, c, ncol = 1)

grid.arrange(a, b, c, ncol = 1)
```

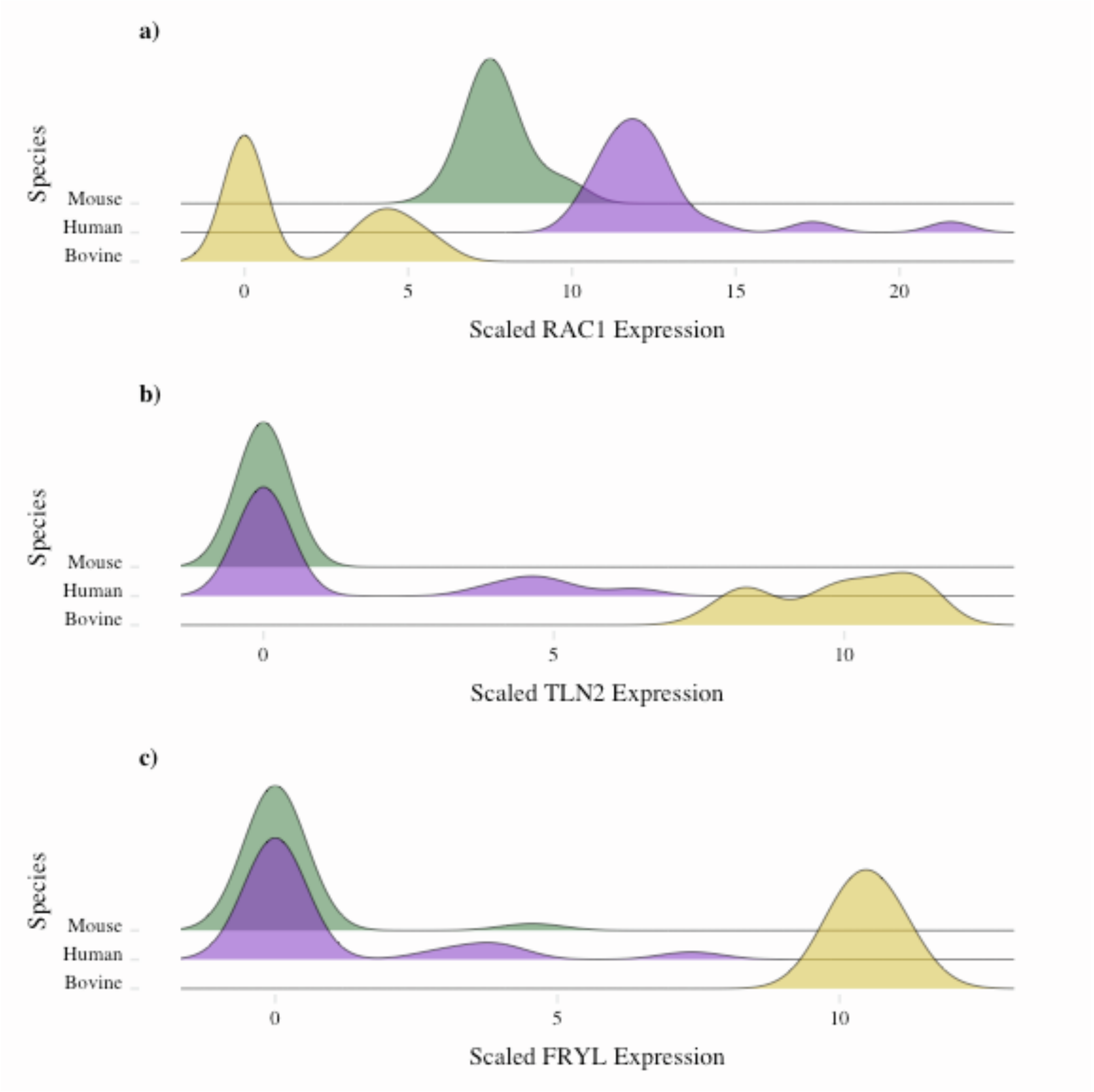

```
ggsave(FIG,
       file="Figures/Species_Density_Plot_Vitro.png",
       width = 3.23,
       height=6,
       units = "in",
       dpi = 600)
```

0.3.3 Classify Collection type for each of the species

Our next RF model aimed to classify collection type (i.e., *in vitro* vs. *in vivo*) for data from mice and cows. Here data from each species were analysed separately. Because data from humans were not collected *in vivo*, they were excluded from these analyses.

```
collection_Data <- data
collection_Data$Collection <- as.factor(IDs$Collection)
names(collection_Data) <- make.names(names(collection_Data))

collection_Data_cows <- collection_Data[which(IDs$Species == "cow"),]
collection_Data_mice <- collection_Data[which(IDs$Species == "mouse"),]

#Run the random forest model identifying vitro/vivo for cows
Collection.mod.cows <- randomForest(y = collection_Data_cows$Collection,
                                    x = collection_Data_cows[,
colnames(collection_Data_cows) != "Collection"],
                                    mtry =  14,
                                    ntree= 20000,
                                    importance=TRUE,
                                    proximity = TRUE,
                                    keep.forest=TRUE,
                                    replace = TRUE)
Collection.mod.cows

##
## Call:
##  randomForest(x = collection_Data_cows[, colnames(collection_Data_cows) !=
"Collection"], y = collection_Data_cows$Collection, ntree = 20000,      mtry
= 14, replace = TRUE, importance = TRUE, proximity = TRUE,      keep.forest =
TRUE)
##                Type of random forest: classification
##                      Number of trees: 20000
## No. of variables tried at each split: 14
##
##         OOB estimate of  error rate: 0%
## Confusion matrix:
##       vitro vivo class.error
## vitro    24    0           0
## vivo      0   12           0

#Run the random forest model identifying vitro/vivo for mice
Collection.mod.mice <- randomForest(y = collection_Data_mice$Collection,
```

```
                                x = collection_Data_mice[,
colnames(collection_Data_mice) != "Collection"],
                                mtry =  14,
                                ntree= 20000,
                                importance=TRUE,
                                proximity = TRUE,
                                keep.forest=TRUE,
                                replace = TRUE)
Collection.mod.mice

##
## Call:
##  randomForest(x = collection_Data_mice[, colnames(collection_Data_mice) !=
"Collection"], y = collection_Data_mice$Collection, ntree = 20000,      mtry
= 14, replace = TRUE, importance = TRUE, proximity = TRUE,      keep.forest =
TRUE)
##                Type of random forest: classification
##                      Number of trees: 20000
## No. of variables tried at each split: 14
##
##         OOB estimate of  error rate: 2.56%
## Confusion matrix:
##       vitro vivo class.error
## vitro    21    1  0.04545455
## vivo      0   17  0.00000000

####################################
# PCA on proximity matrix for cows

res.pca <- PCA(Collection.mod.cows$proximity, graph = FALSE) # Conduct a PCA
on the proximity matrix
PC1 <- res.pca$ind$coord[,1] #Store individual coordinates of PC1 as a vector
PC2 <- res.pca$ind$coord[,2] #Store individual coordinates of PC2 as a vector
PCs.ID <- data.frame(cbind(PC1,PC2)) #Bind the coordinates together as a
dataframe
PCs.ID$Subject <- collection_Data_cows$Collection #Add in collection type to
the df

DIM_1 <- paste("Dim 1 (", round(res.pca$eig[1,2], 1), "%)")
DIM_2 <- paste("Dim 2 (", round(res.pca$eig[2,2], 1), "%)")

#Draw ellipses around the clusters and highlighting the elipsoids rather than
the data points
centroids <- aggregate(cbind(PC1,PC2)~Subject,PCs.ID,mean)
conf.rgn  <- do.call(rbind,lapply(unique(PCs.ID$Subject),function(t)
  data.frame(Subject=as.character(t),
             ellipse(cov(PCs.ID[PCs.ID$Subject==t,1:2]),
                     centre=as.matrix(centroids[t,2:3]),
                     level=0.95),
             stringsAsFactors=FALSE)))
```

```
#Then make the figure
PCA_FIG_Cows <-
  ggplot(PCs.ID, aes(x=PC1, y=PC2, color = Subject), guide = FALSE) +
  geom_hline(aes(yintercept=0), linetype="dashed", lwd = 0.1) +
  geom_vline(aes(xintercept=0), linetype="dashed", lwd = 0.1) +
  geom_path(data=conf.rgn, alpha=0.2, size = 0) +
  geom_polygon(data=conf.rgn, aes(fill = Subject), alpha=0.1, size = 0.1,
show.legend = FALSE) +
  geom_point(size=0.4) +
  theme_bw() +
  ggtitle("a) - Bovine") +
  scale_color_manual(labels=c("Vitro", "Vivo"), values = c("#046C9A", "red"))
+
  scale_fill_manual(labels=c("Vitro", "Vivo"), values = c("#046C9A",
"red"),guide = FALSE) +
  ylab(DIM_2) +
  xlab(DIM_1) +
  theme(panel.grid.major = element_blank(),
        panel.border = element_rect(colour = "black", size=1),
        panel.grid.minor = element_blank(),
        axis.title.x  = element_text(size=10, family = "serif"),
        axis.title.y  = element_text(size=10, family = "serif"),
        plot.title = element_text(size=8, hjust = 0, family = "serif"),
        axis.text.y  = element_text(size=5, family = "serif"),
        axis.text.x  = element_text(size=5, family = "serif"),
        legend.position=c(0.8,0.15),
        legend.background = element_blank(),
        legend.title = element_blank(),
        legend.text = element_text(size=8, family = "serif"),
        legend.key.size = unit(0.3, "cm"),
        legend.key = element_blank())

####################################
# PCA on proximity matrix for mice

res.pca <- PCA(Collection.mod.mice$proximity, graph = FALSE) # Conduct a PCA
on the proximity matrix
PC1 <- res.pca$ind$coord[,1] #Store individual coordinates of PC1 as a vector
PC2 <- res.pca$ind$coord[,2] #Store individual coordinates of PC2 as a vector
PCs.ID <- data.frame(cbind(PC1,PC2)) #Bind the coordinates together as a
dataframe
PCs.ID$Subject <- collection_Data_mice$Collection #Add in Season to the df

DIM_1 <- paste("Dim 1 (", round(res.pca$eig[1,2], 1), "%)")
DIM_2 <- paste("Dim 2 (", round(res.pca$eig[2,2], 1), "%)")

#Draw ellipses around the clusters and highlighting the ellipsoids rather
than the data points
```

```
centroids <- aggregate(cbind(PC1,PC2)~Subject,PCs.ID,mean)
conf.rgn  <- do.call(rbind,lapply(unique(PCs.ID$Subject),function(t)
  data.frame(Subject=as.character(t),
             ellipse(cov(PCs.ID[PCs.ID$Subject==t,1:2]),
                     centre=as.matrix(centroids[t,2:3]),
                     level=0.95),
             stringsAsFactors=FALSE)))

#Then make the figure
PCA_FIG_Mice <-
  ggplot(PCs.ID, aes(x=PC1, y=PC2, color = Subject), guide = FALSE) +
  geom_hline(aes(yintercept=0), linetype="dashed", lwd = 0.1) +
  geom_vline(aes(xintercept=0), linetype="dashed", lwd = 0.1) +
  geom_path(data=conf.rgn, alpha=0.2, size = 0) +
  geom_polygon(data=conf.rgn, aes(fill = Subject),
               alpha=0.1,
               size = 0.1,
               show.legend = FALSE) +
  geom_point(size=0.4) +
  theme_bw() +
  ggtitle("b) - Mouse") +
  scale_color_manual(labels=c("Vitro", "Vivo"),
                     values = c("#046C9A", "red")) +
  scale_fill_manual(labels=c("Vitro", "Vivo"),
                    values = c("#046C9A", "red"),
                    guide = FALSE) +
  ylab(DIM_2) +
  xlab(DIM_1) +
  theme(panel.grid.major = element_blank(),
        panel.border = element_rect(colour = "black", size=1),
        panel.grid.minor = element_blank(),
        axis.title.x  = element_text(size=10, family = "serif"),
        axis.title.y  = element_text(size=10, family = "serif"),
        plot.title = element_text(size=8, hjust = 0, family = "serif"),
        axis.text.y  = element_text(size=5, family = "serif"),
        axis.text.x  = element_text(size=5, family = "serif"),
        legend.position="none",
        legend.background = element_blank(),
        legend.title = element_blank(),
        legend.text = element_text(size=8, family = "serif"),
        legend.key.size = unit(0.3, "cm"),
        legend.key = element_blank())

FIG <- arrangeGrob(PCA_FIG_Cows,
                   PCA_FIG_Mice,
                   ncol = 1)

grid.arrange(PCA_FIG_Cows, PCA_FIG_Mice,ncol = 1)
```

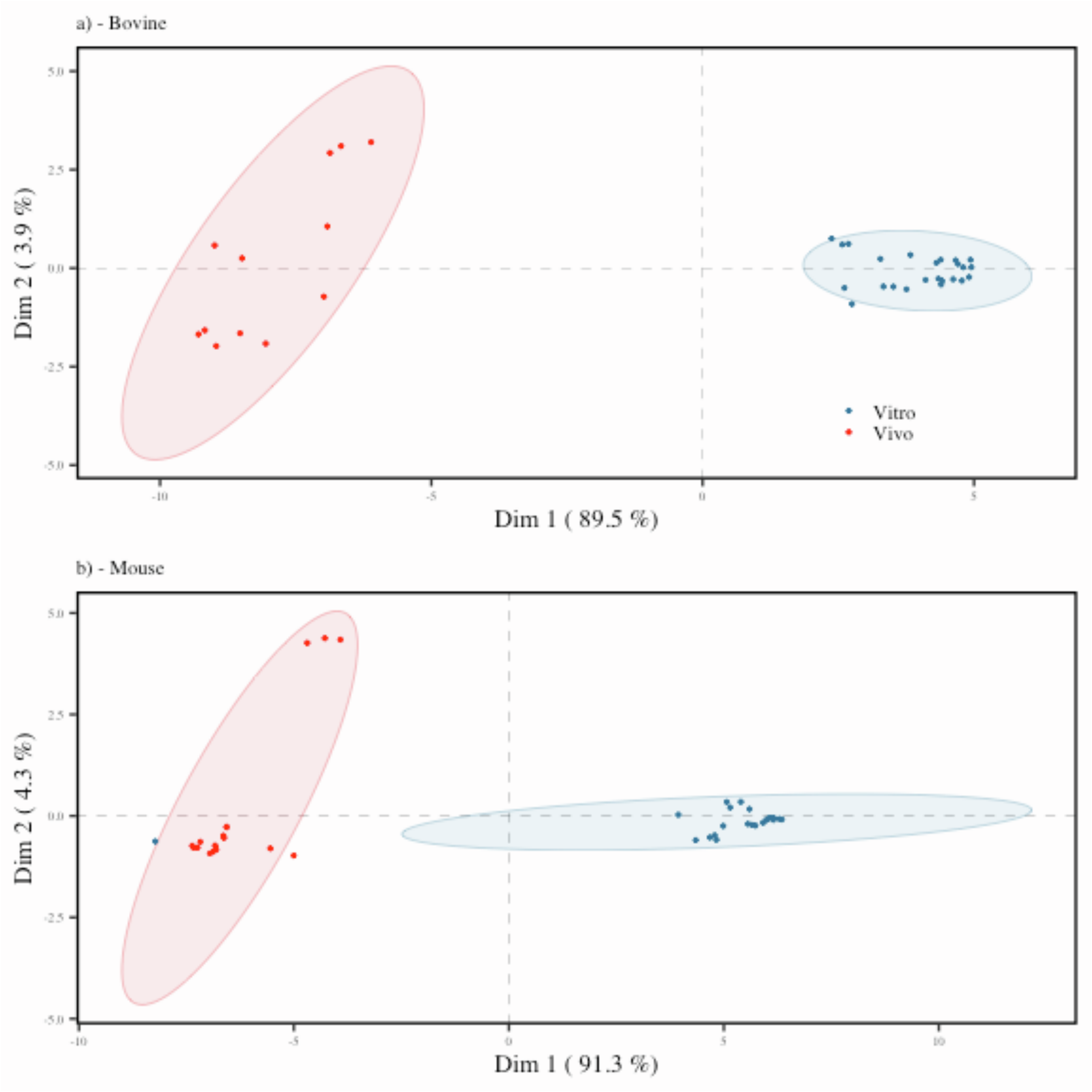


```
ggsave(FIG,
       file="Figures/Classification_Vitro_Vivo.png",
       width = 3.23,
       height=5,
       units = "in",
       dpi  = 600)
```

0.3.4 Classify Stage for each of the species

Our last set of RF models aimed to classify developmental stage for data from mice and cows and humans. Here data from each collection type (i.e., *in vitro* vs. *in vivo*) were analysed separately.

0.3.4.1 In vitro samples

```
stage_Data <- data
stage_Data$Stage <- as.factor(IDs$Stage)
names(stage_Data) <- make.names(names(stage_Data))
```

```
stage_Data_cows <- stage_Data[which(IDs$Species == "cow" & IDs$Collection ==
"vitro"),]
stage_Data_cows$Stage <- factor(stage_Data_cows$Stage)
stage_Data_mice <- stage_Data[which(IDs$Species == "mouse" & IDs$Collection
== "vitro"),]
stage_Data_mice$Stage <- factor(stage_Data_mice$Stage)
stage_Data_humans <- stage_Data[which(IDs$Species == "human"),]
stage_Data_humans$Stage <- factor(stage_Data_humans$Stage)


#Run the random forest model identifying stage in cows
stage.mod.cows <- randomForest(y = stage_Data_cows$Stage,
                               x = stage_Data_cows[,
colnames(stage_Data_cows) != "Stage"],
                               mtry =  14,
                               ntree= 20000,
                               importance=TRUE,
                               proximity = TRUE,
                               keep.forest=TRUE,
                               replace = TRUE)
stage.mod.cows

##
## Call:
##  randomForest(x = stage_Data_cows[, colnames(stage_Data_cows) !=
"Stage"], y = stage_Data_cows$Stage, ntree = 20000, mtry = 14,      replace =
TRUE, importance = TRUE, proximity = TRUE, keep.forest = TRUE)
##                Type of random forest: classification
##                      Number of trees: 20000
## No. of variables tried at each split: 14
##
##         OOB estimate of  error rate: 16.67%
## Confusion matrix:
##     16C 2C 4C 8C BL MII class.error
## 16C   2  0  0  0  1   0   0.3333333
## 2C    0  3  0  0  0   0   0.0000000
## 4C    0  0  2  0  0   1   0.3333333
## 8C    0  0  1  2  0   0   0.3333333
## BL    0  0  0  0  9   0   0.0000000
## MII   0  0  1  0  0   2   0.3333333

#Run the random forest model identifying stage in mice
stage.mod.mice <- randomForest(y = stage_Data_mice$Stage,
                               x = stage_Data_mice[,
colnames(stage_Data_mice) != "Stage"],
                               mtry =  14,
                               ntree= 20000,
                               importance=TRUE,
                               proximity = TRUE,
```

```
                                keep.forest=TRUE,
                                replace = TRUE)
stage.mod.mice

##
## Call:
##  randomForest(x = stage_Data_mice[, colnames(stage_Data_mice) !=
"Stage"], y = stage_Data_mice$Stage, ntree = 20000, mtry = 14,      replace =
TRUE, importance = TRUE, proximity = TRUE, keep.forest = TRUE)
##                Type of random forest: classification
##                      Number of trees: 20000
## No. of variables tried at each split: 14
##
##         OOB estimate of  error rate: 4.55%
## Confusion matrix:
##    2C 4C 8C BL MO class.error
## 2C  4  0  0  0  0        0.00
## 4C  0  4  0  0  0        0.00
## 8C  0  0  3  0  1        0.25
## BL  0  0  0  4  0        0.00
## MO  0  0  0  0  6        0.00

#Run the random forest model identifying stage in humans
stage.mod.humans <- randomForest(y = stage_Data_humans$Stage,
                                 x = stage_Data_humans[,
colnames(stage_Data_humans) != "Stage"],
                                 mtry =  14,
                                 ntree= 20000,
                                 importance=TRUE,
                                 proximity = TRUE,
                                 keep.forest=TRUE,
                                 replace = TRUE)
stage.mod.humans

##
## Call:
##  randomForest(x = stage_Data_humans[, colnames(stage_Data_humans) !=
"Stage"], y = stage_Data_humans$Stage, ntree = 20000, mtry = 14,      replace
= TRUE, importance = TRUE, proximity = TRUE, keep.forest = TRUE)
##                Type of random forest: classification
##                      Number of trees: 20000
## No. of variables tried at each split: 14
##
##         OOB estimate of  error rate: 30%
## Confusion matrix:
##     2C 4C 8C BL MII MO class.error
## 2C   0  3  0  0   0  0   1.0000000
## 4C   0  5  0  0   0  0   0.0000000
## 8C   0  0  3  0   0  0   0.0000000
## BL   0  0  0  2   0  1   0.3333333
```

```
## MII  1  0  0  0   2  0   0.3333333
## MO   0  0  0  1   0  2   0.3333333

####################################
# PCA on proximity matrix for cows

res.pca <- PCA(stage.mod.cows$proximity, graph = FALSE) # Conduct a PCA on
the proximity matrix
PC1 <- res.pca$ind$coord[,1] #Store individual coordinates of PC1 as a vector
PC2 <- res.pca$ind$coord[,2] #Store individual coordinates of PC2 as a vector
PCs.ID <- data.frame(cbind(PC1,PC2)) #Bind the coordinates together as a
dataframe
PCs.ID$Subject <- stage_Data_cows$Stage #Add in Stage to the df
PCs.ID$Group <- factor(IDs[which(IDs$Species == "cow" & IDs$Collection ==
"vitro"),"Stage"])

DIM_1 <- paste("Dim 1 (", round(res.pca$eig[1,2], 1), "%)")
DIM_2 <- paste("Dim 2 (", round(res.pca$eig[2,2], 1), "%)")

#Draw ellipses around the clusters and highlighting the elipsoids rather than
the data points
centroids <- aggregate(cbind(PC1,PC2)~Group,PCs.ID,mean)
conf.rgn  <- do.call(rbind,lapply(unique(PCs.ID$Group),function(t)
  data.frame(Group=as.character(t),
             ellipse(cov(PCs.ID[PCs.ID$Group==t,1:2]),
                     centre=as.matrix(centroids[t,2:3]),
                     level=0.95),
             stringsAsFactors=FALSE)))

#Then make the figure
PCA_FIG_Cows <-
  ggplot(PCs.ID, aes(x=PC1, y=PC2), guide = FALSE) +
  geom_hline(aes(yintercept=0), linetype="dashed", lwd = 0.1) +
  geom_vline(aes(xintercept=0), linetype="dashed", lwd = 0.1) +
  geom_path(data=conf.rgn, alpha=0.2, size = 0) +
  geom_polygon(data=conf.rgn,
               aes(fill = Group, colour = Group),
               alpha=0.1,
               size = 0.1,
               show.legend = FALSE) +
  geom_point(size=0.4, aes(color = Subject)) +
  theme_bw() +
  #scale_color_viridis(discrete = TRUE) +
  #scale_fill_viridis(guide = FALSE, discrete = T) +
  ggtitle("a) - Bovine") +
  ylab(DIM_2) +
  xlab(DIM_1) +
  theme(panel.grid.major = element_blank(),
        panel.border = element_rect(colour = "black", size=1),
        panel.grid.minor = element_blank(),
```

```
        axis.title.x  = element_text(size=10, family = "serif"),
        axis.title.y  = element_text(size=10, family = "serif"),
        plot.title = element_text(size=8, hjust = 0, family = "serif"),
        axis.text.y  = element_text(size=5, family = "serif"),
        axis.text.x  = element_text(size=5, family = "serif"),
        legend.position= "right",
        legend.background = element_blank(),
        legend.title = element_blank(),
        legend.text = element_text(size=8, family = "serif"),
        legend.key.size = unit(0.3, "cm"),
        legend.key = element_blank())

####################################
# PCA on proximity matrix for mice

res.pca <- PCA(stage.mod.mice$proximity, graph = FALSE) # Conduct a PCA on
the proximity matrix
PC1 <- res.pca$ind$coord[,1] #Store individual coordinates of PC1 as a vector
PC2 <- res.pca$ind$coord[,2] #Store individual coordinates of PC2 as a vector
PCs.ID <- data.frame(cbind(PC1,PC2)) #Bind the coordinates together as a
dataframe
PCs.ID$Subject <- stage_Data_mice$Stage #Add in Season to the df
PCs.ID$Group <- factor(IDs[which(IDs$Species == "mouse" & IDs$Collection ==
"vitro"),"Stage"])

DIM_1 <- paste("Dim 1 (", round(res.pca$eig[1,2], 1), "%)")
DIM_2 <- paste("Dim 2 (", round(res.pca$eig[2,2], 1), "%)")

#Draw ellipses around the clusters and highlighting the elipsoids rather than
the data points
centroids <- aggregate(cbind(PC1,PC2)~Group,PCs.ID,mean)
conf.rgn  <- do.call(rbind,lapply(unique(PCs.ID$Group),function(t)
  data.frame(Group=as.character(t),
             ellipse(cov(PCs.ID[PCs.ID$Group==t,1:2]),
                     centre=as.matrix(centroids[t,2:3]),
                     level=0.95),
             stringsAsFactors=FALSE)))

#Then make the figure
PCA_FIG_Mice <-
  ggplot(PCs.ID, aes(x=PC1, y=PC2), guide = FALSE) +
  geom_hline(aes(yintercept=0), linetype="dashed", lwd = 0.1) +
  geom_vline(aes(xintercept=0), linetype="dashed", lwd = 0.1) +
  geom_path(data=conf.rgn, alpha=0.2, size = 0) +
  geom_polygon(data=conf.rgn,
               aes(fill = Group, colour = Group),
               alpha=0.1,
               size = 0.1,
               show.legend = FALSE) +
```

```
  geom_point(size=0.4, aes(color = Subject)) +
  theme_bw() +
  #scale_color_viridis(discrete = TRUE) +
  #scale_fill_viridis(guide = FALSE, discrete = T) +
  ggtitle("b) - Mouse") +
  ylab(DIM_2) +
  xlab(DIM_1) +
  theme(panel.grid.major = element_blank(),
        panel.border = element_rect(colour = "black", size=1),
        panel.grid.minor = element_blank(),
        axis.title.x  = element_text(size=10, family = "serif"),
        axis.title.y  = element_text(size=10, family = "serif"),
        plot.title = element_text(size=8, hjust = 0, family = "serif"),
        axis.text.y  = element_text(size=5, family = "serif"),
        axis.text.x  = element_text(size=5, family = "serif"),
        legend.position= "right",
        legend.background = element_blank(),
        legend.title = element_blank(),
        legend.text = element_text(size=8, family = "serif"),
        legend.key.size = unit(0.3, "cm"),
        legend.key = element_blank())

####################################
# PCA on proximity matrix for humans

res.pca <- PCA(stage.mod.humans$proximity, graph = FALSE) # Conduct a PCA on
the proximity matrix
PC1 <- res.pca$ind$coord[,1] #Store individual coordinates of PC1 as a vector
PC2 <- res.pca$ind$coord[,2] #Store individual coordinates of PC2 as a vector
PCs.ID <- data.frame(cbind(PC1,PC2)) #Bind the coordinates together as a
dataframe
PCs.ID$Subject <- stage_Data_humans$Stage #Add in Season to the df
PCs.ID$Group <- factor(IDs[which(IDs$Species == "human"),"Stage"])

DIM_1 <- paste("Dim 1 (", round(res.pca$eig[1,2], 1), "%)")
DIM_2 <- paste("Dim 2 (", round(res.pca$eig[2,2], 1), "%)")

#Draw ellipses around the clusters and highlighting the elipsoids rather than
the data points
centroids <- aggregate(cbind(PC1,PC2)~Group,PCs.ID,mean)
conf.rgn  <- do.call(rbind,lapply(unique(PCs.ID$Group),function(t)
  data.frame(Group=as.character(t),
             ellipse(cov(PCs.ID[PCs.ID$Group==t,1:2]),
                     centre=as.matrix(centroids[t,2:3]),
                     level=0.95),
             stringsAsFactors=FALSE)))

#Then make the figure
PCA_FIG_Humans <-
```

```
  ggplot(PCs.ID, aes(x=PC1, y=PC2), guide = FALSE) +
  geom_hline(aes(yintercept=0), linetype="dashed", lwd = 0.1) +
  geom_vline(aes(xintercept=0), linetype="dashed", lwd = 0.1) +
  geom_path(data=conf.rgn, alpha=0.2, size = 0) +
  geom_polygon(data=conf.rgn,
               aes(fill = Group, colour = Group),
               alpha=0.1,
               size = 0.1,
               show.legend = FALSE) +
  geom_point(size=0.4, aes(color = Subject)) +
  theme_bw() +
  #scale_color_viridis(discrete = TRUE) +
  #scale_fill_viridis(guide = FALSE, discrete = T) +
  ggtitle("c) - Humans") +
  ylab(DIM_2) +
  xlab(DIM_1) +
  theme(panel.grid.major = element_blank(),
        panel.border = element_rect(colour = "black", size=1),
        panel.grid.minor = element_blank(),
        axis.title.x  = element_text(size=10, family = "serif"),
        axis.title.y  = element_text(size=10, family = "serif"),
        plot.title = element_text(size=8, hjust = 0, family = "serif"),
        axis.text.y  = element_text(size=5, family = "serif"),
        axis.text.x  = element_text(size=5, family = "serif"),
        legend.position= "right",
        legend.background = element_blank(),
        legend.title = element_blank(),
        legend.text = element_text(size=8, family = "serif"),
        legend.key.size = unit(0.3, "cm"),
        legend.key = element_blank())

FIG <- arrangeGrob(PCA_FIG_Cows,
                   PCA_FIG_Mice,
                   PCA_FIG_Humans,
                   ncol = 1)

ggsave(FIG,
       file="Figures/Classification_Stage_Vitro.png",
       width = 4,
       height=7,
       units = "in",
       dpi = 600)
```

0.3.4.2 In vivo samples

```
stage_Data <- data
stage_Data$Stage <- as.factor(IDs$Stage)
names(stage_Data) <- make.names(names(stage_Data))

stage_Data_cows <- stage_Data[which(IDs$Species == "cow" & IDs$Collection ==
"vivo"),]
```

```
stage_Data_cows$Stage <- factor(stage_Data_cows$Stage)
stage_Data_mice <- stage_Data[which(IDs$Species == "mouse" & IDs$Collection
== "vivo"),]
stage_Data_mice$Stage <- factor(stage_Data_mice$Stage)


#Run the random forest model identifying stage in cows
stage.mod.cows <- randomForest(y = stage_Data_cows$Stage,
                               x = stage_Data_cows[,
colnames(stage_Data_cows) != "Stage"],
                               mtry =  14,
                               ntree= 20000,
                               importance=TRUE,
                               proximity = TRUE,
                               keep.forest=TRUE,
                               replace = TRUE)

stage.mod.cows

##
## Call:
##  randomForest(x = stage_Data_cows[, colnames(stage_Data_cows) !=
"Stage"], y = stage_Data_cows$Stage, ntree = 20000, mtry = 14,      replace =
TRUE, importance = TRUE, proximity = TRUE, keep.forest = TRUE)
##                Type of random forest: classification
##                      Number of trees: 20000
## No. of variables tried at each split: 14
##
##         OOB estimate of  error rate: 75%
## Confusion matrix:
##     16C 2C 4C 8C BL MII class.error
## 16C   1  0  0  1  0   0         0.5
## 2C    0  0  1  0  0   1         1.0
## 4C    0  2  0  0  0   0         1.0
## 8C    2  0  0  0  0   0         1.0
## BL    0  0  0  0  2   0         0.0
## MII   0  2  0  0  0   0         1.0

varImpPlot(stage.mod.cows, type=1, scale = FALSE)
```

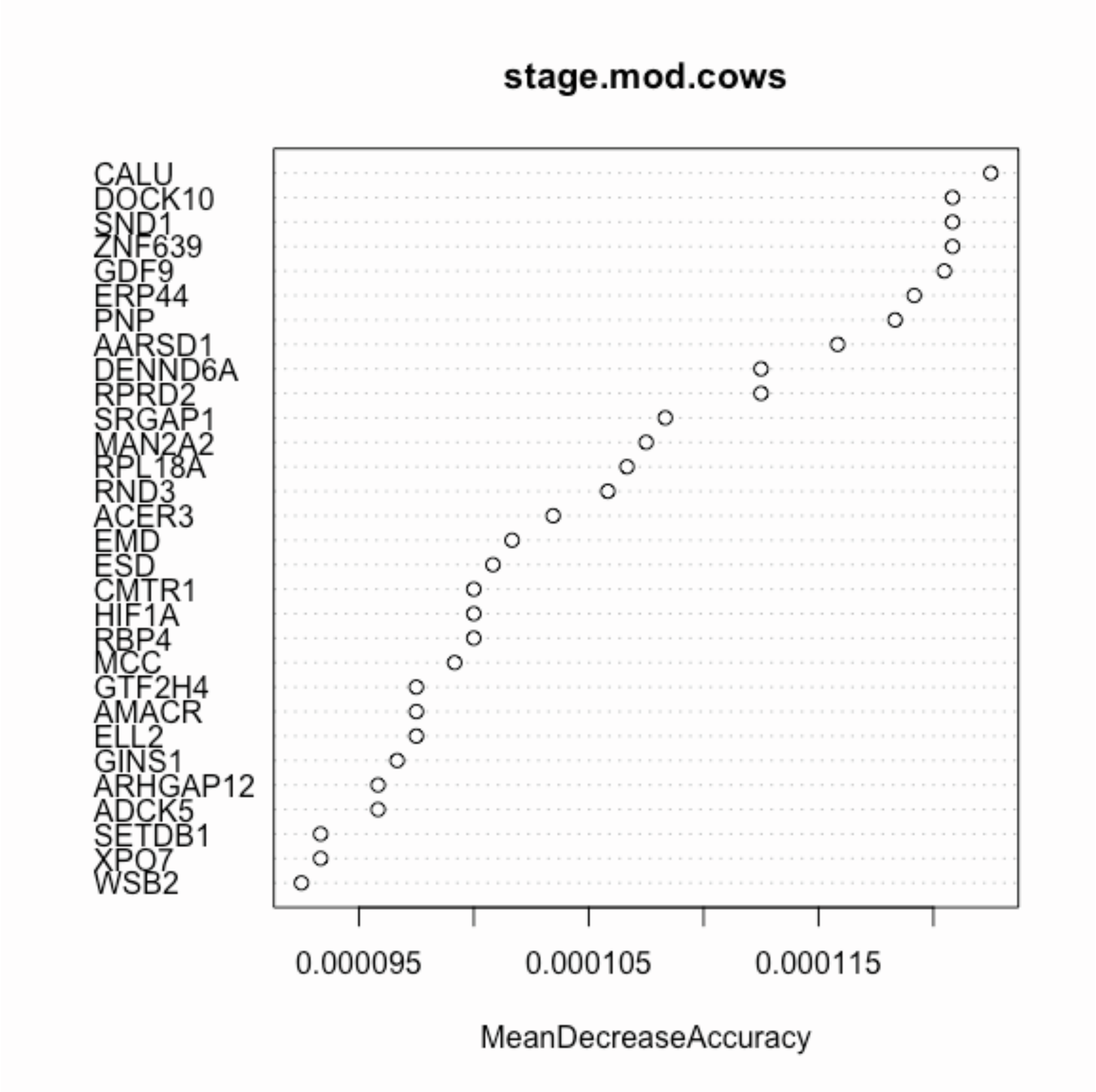


```
#Run the random forest model identifying stage in mice
stage.mod.mice <- randomForest(y = stage_Data_mice$Stage,
                               x = stage_Data_mice[,
colnames(stage_Data_mice) != "Stage"],
                               mtry =  14,
                               ntree= 20000,
                               importance=TRUE,
                               proximity = TRUE,
                               keep.forest=TRUE,
                               replace = TRUE)

stage.mod.mice

##
## Call:
##  randomForest(x = stage_Data_mice[, colnames(stage_Data_mice) !=
"Stage"], y = stage_Data_mice$Stage, ntree = 20000, mtry = 14,      replace =
```

```
TRUE, importance = TRUE, proximity = TRUE, keep.forest = TRUE)
##                Type of random forest: classification
##                      Number of trees: 20000
## No. of variables tried at each split: 14
##
##         OOB estimate of  error rate: 11.76%
## Confusion matrix:
##     2C 4C 8C BL MII MO class.error
## 2C   4  0  0  0   0  0           0
## 4C   0  4  0  0   0  0           0
## 8C   0  2  0  0   0  0           1
## BL   0  0  0  3   0  0           0
## MII  0  0  0  0   2  0           0
## MO   0  0  0  0   0  2           0

varImpPlot(stage.mod.mice, type=1, scale = FALSE)

####################################
# PCA on proximity matrix for cows

res.pca <- PCA(stage.mod.cows$proximity, graph = FALSE) # Conduct a PCA on
the proximity matrix
PC1 <- res.pca$ind$coord[,1] #Store individual coordinates of PC1 as a vector
PC2 <- res.pca$ind$coord[,2] #Store individual coordinates of PC2 as a vector
PCs.ID <- data.frame(cbind(PC1,PC2)) #Bind the coordinates together as a
dataframe
PCs.ID$Subject <- stage_Data_cows$Stage #Add in Season to the df
PCs.ID$Group <- factor(IDs[which(IDs$Species == "cow" & IDs$Collection ==
"vivo"),"Stage"])

DIM_1 <- paste("Dim 1 (", round(res.pca$eig[1,2], 1), "%)")
DIM_2 <- paste("Dim 2 (", round(res.pca$eig[2,2], 1), "%)")

#Draw ellipses around the clusters and highlighting the elipsoids rather than
the data points
centroids <- aggregate(cbind(PC1,PC2)~Group,PCs.ID,mean)
conf.rgn  <- do.call(rbind,lapply(unique(PCs.ID$Group),function(t)
  data.frame(Group=as.character(t),
             ellipse(cov(PCs.ID[PCs.ID$Group==t,1:2]),
                     centre=as.matrix(centroids[t,2:3]),
                     level=0.95),
             stringsAsFactors=FALSE)))

#Then make the figure
PCA_FIG_Cows <-
  ggplot(PCs.ID, aes(x=PC1, y=PC2), guide = FALSE) +
  geom_hline(aes(yintercept=0), linetype="dashed", lwd = 0.1) +
  geom_vline(aes(xintercept=0), linetype="dashed", lwd = 0.1) +
  geom_path(data=conf.rgn, alpha=0.2, size = 0) +
  geom_polygon(data=conf.rgn,
```

```
               aes(fill = Group, colour = Group),
               alpha=0.1,
               size = 0.1,
               show.legend = FALSE) +
  geom_point(size=0.4, aes(color = Subject)) +
  theme_bw() +
  #scale_color_viridis(discrete = TRUE) +
  #scale_fill_viridis(guide = FALSE, discrete = T) +
  ggtitle("a) - Bovine") +
  ylab(DIM_2) +
  xlab(DIM_1) +
  theme(panel.grid.major = element_blank(),
        panel.border = element_rect(colour = "black", size=1),
        panel.grid.minor = element_blank(),
        axis.title.x  = element_text(size=10, family = "serif"),
        axis.title.y  = element_text(size=10, family = "serif"),
        plot.title = element_text(size=8, hjust = 0, family = "serif"),
        axis.text.y  = element_text(size=5, family = "serif"),
        axis.text.x  = element_text(size=5, family = "serif"),
        legend.position= "right",
        legend.background = element_blank(),
        legend.title = element_blank(),
        legend.text = element_text(size=8, family = "serif"),
        legend.key.size = unit(0.3, "cm"),
        legend.key = element_blank())

####################################
# PCA on proximity matrix for mice

res.pca <- PCA(stage.mod.mice$proximity, graph = FALSE) # Conduct a PCA on
the proximity matrix
PC1 <- res.pca$ind$coord[,1] #Store individual coordinates of PC1 as a vector
PC2 <- res.pca$ind$coord[,2] #Store individual coordinates of PC2 as a vector
PCs.ID <- data.frame(cbind(PC1,PC2)) #Bind the coordinates together as a
dataframe
PCs.ID$Subject <- stage_Data_mice$Stage #Add in Season to the df
PCs.ID$Group <- factor(IDs[which(IDs$Species == "mouse" & IDs$Collection ==
"vivo"),"Stage"])

DIM_1 <- paste("Dim 1 (", round(res.pca$eig[1,2], 1), "%)")
DIM_2 <- paste("Dim 2 (", round(res.pca$eig[2,2], 1), "%)")

#Draw ellipses around the clusters and highlighting the elipsoids rather than
the data points
centroids <- aggregate(cbind(PC1,PC2)~Group,PCs.ID,mean)
conf.rgn  <- do.call(rbind,lapply(unique(PCs.ID$Group),function(t)
  data.frame(Group=as.character(t),
             ellipse(cov(PCs.ID[PCs.ID$Group==t,1:2]),
                     centre=as.matrix(centroids[t,2:3]),
```

```
                    level=0.95),
            stringsAsFactors=FALSE)))

#Then make the figure
PCA_FIG_Mice <-
  ggplot(PCs.ID, aes(x=PC1, y=PC2), guide = FALSE) +
  geom_hline(aes(yintercept=0), linetype="dashed", lwd = 0.1) +
  geom_vline(aes(xintercept=0), linetype="dashed", lwd = 0.1) +
  geom_path(data=conf.rgn, alpha=0.2, size = 0) +
  geom_polygon(data=conf.rgn,
               aes(fill = Group, colour = Group),
               alpha=0.1,
               size = 0.1,
               show.legend = FALSE) +
  geom_point(size=0.4, aes(color = Subject)) +
  theme_bw() +
  #scale_color_viridis(discrete = TRUE) +
  #scale_fill_viridis(guide = FALSE, discrete = T) +
  ggtitle("b) - Mouse") +
  ylab(DIM_2) +
  xlab(DIM_1) +
  theme(panel.grid.major = element_blank(),
        panel.border = element_rect(colour = "black", size=1),
        panel.grid.minor = element_blank(),
        axis.title.x  = element_text(size=10, family = "serif"),
        axis.title.y  = element_text(size=10, family = "serif"),
        plot.title = element_text(size=8, hjust = 0, family = "serif"),
        axis.text.y  = element_text(size=5, family = "serif"),
        axis.text.x  = element_text(size=5, family = "serif"),
        legend.position= "right",
        legend.background = element_blank(),
        legend.title = element_blank(),
        legend.text = element_text(size=8, family = "serif"),
        legend.key.size = unit(0.3, "cm"),
        legend.key = element_blank())

FIG <- arrangeGrob(PCA_FIG_Cows,
                   PCA_FIG_Mice,
                   ncol = 1)
```

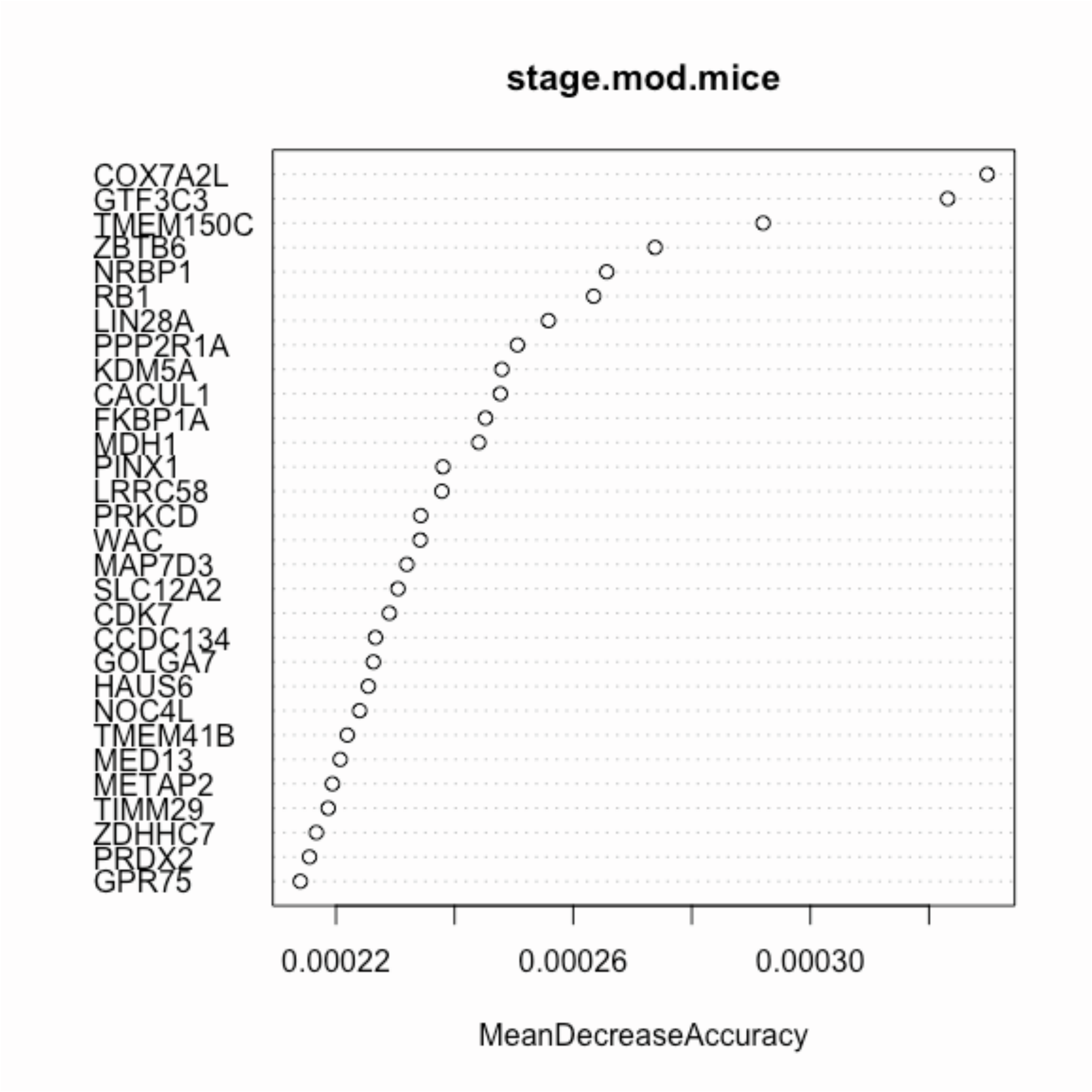


```
ggsave(FIG,
       file="Figures/Classification_Stage_Vivo.png",
       width = 3.23,
       height=5,
       units = "in",
       dpi = 600)
```

0.4 Differentially Expressed Genes (DEG)

Differentially expressed genes (DEGs) of normalized data were identified using the R package `limma`. An adjusted p-value to correct for multiple testing was calculated using the Benjamini–Hochberg method. The most widely used correction for genomic studies is the Benjamini-Hochberg (BH) correction, that aims to control the FDR across significant genes.

0.4.1 Intra-specific DEGs

```
#Load in metadata
GENES <- read.csv("metabolism_genes.csv")
KEEPERS <- read.csv("metaboloepig_genes.csv")
#Convert to a numeric matrix
DATA <- as.matrix(t(data))


#Create the design matrix
design <- cbind(species = as.numeric(as.factor(IDs$Species)),
                stage = as.numeric(as.factor(IDs$Stage)),
                collection = as.numeric(as.factor(IDs$Collection)))

STAGE_NAMES <- unique(as.factor(IDs$Stage))

#Empty list to fill
RES <- list()

#Subset the data and create the appropriate design matrix
#Pick which species you're interested in cow = 1, human = 2, mouse = 3

for(i in 1:length(unique(IDs$Species))){
  SPECIES <- c(i)
  design2 <- design[which(design[,1] == SPECIES),]
  DATA_Species <- DATA[,which(design[,1] == SPECIES)]

  #################################
  # Do all the pairwise comparisons of the different stages for each
collection type

  #The different combinations of stages to test
  stage_tests <- combn(unique(design2[,2]), 2)

  #Loop over the combinations of stage(s) 16C = 1; 2C = 2; 4C = 3; 8C = 4; BL
= 5; MII = 6; MO = 7
  for(j in 1:ncol(stage_tests)){
    STAGES <- c(stage_tests[,j])
    design3 <- design2[which(design2[,2] %in% STAGES),]
    DATA_Stages <- DATA_Species[,which(design2[,2] %in% STAGES)]

    #Added this check because humans don't have in vivo
    if(SPECIES != 2){
      #Loop over collection type(s): vitro = 1; vivo = 2
      for(k in 1:2){
        COLLECTION <- k
        design4 <- design3[which(design3[,3] %in% COLLECTION),]
        DATA_test <- DATA_Stages[,which(design3[,3] %in% COLLECTION)]

        if(length(unique(design4[,2])) > 1){
```

```
          test <- lmFit(DATA_test, design = model.matrix(~ 1 + design4[,2]))
          test2 <- eBayes(test)
          test3 <- topTable(test2, number = length(test2$coefficients),
sort.by = "none")
          results <- data.frame(test3$adj.P.Val)
          names(results) <- paste(unique(IDs$Species)[i],
                                  sort(unique(IDs$Collection))[k],

paste(sort(unique(as.factor(IDs$Stage)))[STAGES][1],
sort(unique(as.factor(IDs$Stage)))[STAGES][2], sep = "vs"),
                                  sep = "_")

          png(file=paste("Figures/Volcano_Plots/",
                         unique(IDs$Species)[i],
                         sort(unique(IDs$Collection))[k],
                         paste(sort(unique(as.factor(IDs$Stage)))[STAGES][1],
sort(unique(as.factor(IDs$Stage)))[STAGES][2], sep = "vs"),
                         ".png", sep = ""),
              type="cairo",
              units = "in",
              width = 6, height = 6,
              res = 300)
          volcanoplot(test2, col = ifelse(test3$adj.P.Val< 0.05, "red",
"#046C9A"))
          dev.off()

          RES[[length(RES)+1]] <- results
        }
      } #Closes the loop over collection type
    } else {

      #Loop over collection type(s): vitro = 1; vivo = 2
      for(k in 1:2){
        COLLECTION <- k
        design4 <- design3[which(design3[,3] %in% COLLECTION),]
        DATA_test <- DATA_Stages[,which(design3[,3] %in% COLLECTION)]

        if(length(unique(design4[,2])) > 1){
          test <- lmFit(DATA_test, design = model.matrix(~ 1 + design4[,2]))
          test2 <- eBayes(test)
          test3 <- topTable(test2, number = length(test2$coefficients),
sort.by = "none")
          results <- data.frame(test3$adj.P.Val)
          names(results) <- paste(unique(IDs$Species)[i],
                                  sort(unique(IDs$Collection))[k],

paste(sort(unique(as.factor(IDs$Stage)))[STAGES][1],
sort(unique(as.factor(IDs$Stage)))[STAGES][2], sep = "vs"),
                                  sep = "_")
```

```
          png(file=paste("Figures/Volcano_Plots/",
                         unique(IDs$Species)[i],
                         sort(unique(IDs$Collection))[k],
                         paste(sort(unique(as.factor(IDs$Stage)))[STAGES][1],
sort(unique(as.factor(IDs$Stage)))[STAGES][2], sep = "vs"),
                         ".png", sep = ""),
              type="cairo",
              units = "in",
              width = 6, height = 6,
              res = 300)
          volcanoplot(test2, col = ifelse(test3$adj.P.Val< 0.05, "red",
"#046C9A"))
          dev.off()

          RES[[length(RES)+1]] <- results
        }
      } #Closes the loop over collection type
    }
  } #Closes the loop over the stage types


  #################################
  # Do all the pairwise comparisons of the different collection types for
each DATA_Stages

  #Ignore these these for humans
  if(SPECIES != 2){
    #Loop over the combinations of stage(s) 16C = 1; 2C = 2; 4C = 3; 8C = 4;
BL = 5; MII = 6; MO = 7
    for(m in 1:length(unique(design2[,2]))){
      STAGES <- unique(design2[,2])[m]
      design3 <- design2[which(design2[,2] %in% STAGES),]
      DATA_Stages <- DATA_Species[,which(design2[,2] %in% STAGES)]

      if(length(unique(design3[,3])) > 1){

        test <- lmFit(DATA_Stages, design = model.matrix(~ 1 + design3[,3]))
        test2 <- eBayes(test)
        test3 <- topTable(test2, number = length(test2$coefficients), sort.by
= "none")
        results <- data.frame(test3$adj.P.Val)
        names(results) <- paste(unique(IDs$Species)[i],
                                sort(unique(as.factor(IDs$Stage)))[STAGES],
                                "vitro - vivo",
                                sep = "_")


        png(file=paste("Figures/Volcano_Plots/",
```

```
                       unique(IDs$Species)[i],
                       sort(unique(as.factor(IDs$Stage)))[STAGES],
                       "vitro - vivo",
                       ".png", sep = ""),
            type="cairo",
            units = "in",
            width = 6, height = 6,
            res = 300)
        volcanoplot(test2, col = ifelse(test3$adj.P.Val< 0.05, "red",
"#046C9A"))
        dev.off()


        RES[[length(RES)+1]] <- results


      }
    } #Closes the loop over the stage types


  }
} #Closes the top level loop


hist(unlist(RES), main = "Adjusted p-values")
```

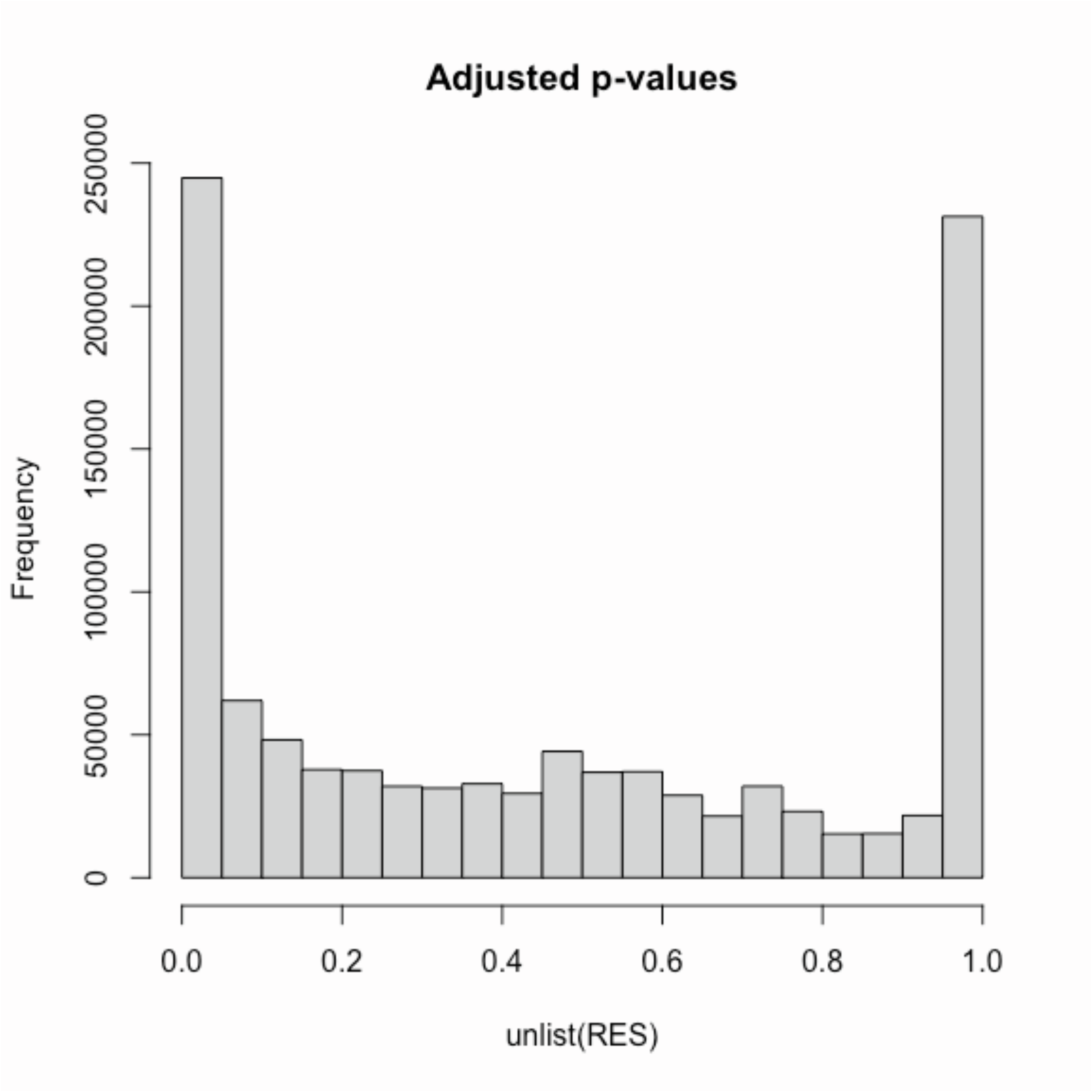


```
RESULTS <- do.call(cbind, RES)
row.names(RESULTS) <- NAMES
write.csv(RESULTS, file = "DEG_Analysis_FINAL.csv", row.names = TRUE)
pvalues <- RESULTS
REDUCED <- RESULTS[(row.names(RESULTS) %in% stringr::str_trim(KEEPERS[,1])),]
write.csv(REDUCED, file = "DEG_Analysis_Reduced_FINAL.csv", row.names = TRUE)



############## Get info on up or down regulated
RES <- list()
#Subset the data and create the appropriate design matrix
#Pick which species you're interested in cow = 1, human = 2, mouse = 3
for(i in 1:length(unique(IDs$Species))){
  SPECIES <- c(i)
  design2 <- design[which(design[,1] == SPECIES),]
```

```
  DATA_Species <- DATA[,which(design[,1] == SPECIES)]

  #################################
  # Do all the pairwise comparisons of the different stages for each
collection type

  #The different combinations of stages to test
  stage_tests <- combn(unique(design2[,2]), 2)

  #Loop over the combinations of stage(s) 16C = 1; 2C = 2; 4C = 3; 8C = 4; BL
= 5; MII = 6; MO = 7
  for(j in 1:ncol(stage_tests)){
    STAGES <- c(stage_tests[,j])
    design3 <- design2[which(design2[,2] %in% STAGES),]
    DATA_Stages <- DATA_Species[,which(design2[,2] %in% STAGES)]


    #Added this because humans don't have in vivo
    if(SPECIES != 2){
      #Loop over collection type(s): vitro = 1; vivo = 2
      for(k in 1:2){
        COLLECTION <- k
        design4 <- design3[which(design3[,3] %in% COLLECTION),]
        DATA_test <- DATA_Stages[,which(design3[,3] %in% COLLECTION)]

        if(length(unique(design4[,2])) > 1){
          test <- lmFit(DATA_test, design = model.matrix(~ 1 + design4[,2]))
          test2 <- eBayes(test)
          test3 <- topTable(test2, number = length(test2$coefficients),
sort.by = "none")
          results <- data.frame(test3$logFC)
          names(results) <- paste(unique(IDs$Species)[i],
                                  sort(unique(IDs$Collection))[k],

paste(sort(unique(as.factor(IDs$Stage)))[STAGES][1],
sort(unique(as.factor(IDs$Stage)))[STAGES][2], sep = "vs"),
                                  sep = "_")
          RES[[length(RES)+1]] <- results
        }
      } #Closes the loop over collection type
    } else {

      #Loop over collection type(s): vitro = 1; vivo = 2
      for(k in 1:2){
        COLLECTION <- k
        design4 <- design3[which(design3[,3] %in% COLLECTION),]
        DATA_test <- DATA_Stages[,which(design3[,3] %in% COLLECTION)]

        if(length(unique(design4[,2])) > 1){
```

```
          test <- lmFit(DATA_test, design = model.matrix(~ 1 + design4[,2]))
          test2 <- eBayes(test)
          test3 <- topTable(test2, number = length(test2$coefficients),
sort.by = "none")
          results <- data.frame(test3$logFC)
          names(results) <- paste(unique(IDs$Species)[i],
                                  sort(unique(IDs$Collection))[k],

paste(sort(unique(as.factor(IDs$Stage)))[STAGES][1],
sort(unique(as.factor(IDs$Stage)))[STAGES][2], sep = "vs"),
                                  sep = "_")
          RES[[length(RES)+1]] <- results
        }
      } #Closes the loop over collection type
    }
  } #Closes the loop over the stage types





  #################################
  # Do all the pairwise comparisons of the different collection types for
each DATA_Stages

  #Ignore these these for humans
  if(SPECIES != 2){
    #Loop over the combinations of stage(s) 16C = 1; 2C = 2; 4C = 3; 8C = 4;
BL = 5; MII = 6; MO = 7
    for(m in 1:length(unique(design2[,2]))){
      STAGES <- unique(design2[,2])[m]
      design3 <- design2[which(design2[,2] %in% STAGES),]
      DATA_Stages <- DATA_Species[,which(design2[,2] %in% STAGES)]

      if(length(unique(design3[,3])) > 1){

        test <- lmFit(DATA_Stages, design = model.matrix(~ 1 + design3[,3]))
        test2 <- eBayes(test)
        test3 <- topTable(test2, number = length(test2$coefficients), sort.by
= "none")
        results <- data.frame(test3$logFC)
        names(results) <- paste(unique(IDs$Species)[i],
                                sort(unique(as.factor(IDs$Stage)))[STAGES],
                                "vitro - vivo",
                                sep = "_")
        RES[[length(RES)+1]] <- results

      }
    } #Closes the loop over the stage types
```

```
  }
} #Closes the top level loop



RESULTS <- do.call(cbind, RES)
row.names(RESULTS) <- NAMES
write.csv(RESULTS, file = "DEG_Analysis_log2FC_FINAL.csv", row.names = TRUE)
REDUCED <- RESULTS[(row.names(RESULTS) %in% stringr::str_trim(KEEPERS[,1])),]
write.csv(REDUCED, file = "DEG_Analysis_log2FC_Reduced_FINAL.csv", row.names
= TRUE)

# numbers of significant up and down regulated for each and volcano plots
REGULATION <- RESULTS[0,]

for(i in 1:ncol(RESULTS)){

  SIGS <- RESULTS[which(pvalues[,i] < 0.05),i]

  REGULATION[1,i] <- length(which(SIGS < 0))

  REGULATION[2,i] <- length(which(SIGS > 0))
}

row.names(REGULATION) <- c("down", "up")

write.csv(REGULATION, file = "Number_Sig_FINAL.csv", row.names = TRUE)
```

0.4.2 Inter-specific DEGs

```
RES <- list()

for(i in 1:length(unique(IDs$Stage))){
  STAGE <- c(i)
  design2 <- design[which(design[,2] == STAGE),]
  DATA_Stage <- DATA[,which(design[,2] == STAGE)]

  if(length(unique(design2[,1]))>1){
    ##################################
    # Do all the pairwise comparisons of the different stages for each
collection type

    #The different combinations of species to test
    species_tests <- combn(unique(design2[,1]), 2)

    for(j in 1:ncol(species_tests)){
      SPECIES <- c(species_tests[,j])
      design3 <- design2[which(design2[,1] %in% SPECIES),]
      DATA_Stages <- DATA_Stage[,which(design2[,1] %in% SPECIES)]
```

```
      design4 <- design3[which(design3[,3] == 2 & design3[,1] == 1),]
      design4 <- rbind(design4, design3[which(design3[,3] == 1 & design3[,1]
== 2),])
      design4 <- rbind(design4, design3[which(design3[,3] == 2 & design3[,1]
== 3),])
      DATA_Stages <-
DATA_Stages[,as.numeric(row.names(plyr::match_df(as.data.frame(design3),
as.data.frame(design4))))]


      test <- lmFit(DATA_Stages, design = model.matrix(~ 1 + design4[,1]))
      test2 <- eBayes(test)
      test3 <- topTable(test2, number = length(test2$coefficients), sort.by =
"none")
      results <- data.frame(test3$adj.P.Val)
      names(results) <- paste(sort(unique(as.factor(IDs$Stage)))[i],

paste(sort(unique(as.factor(IDs$Species)))[SPECIES][1],
sort(unique(as.factor(IDs$Species)))[SPECIES][2], sep = "vs"),
                              sep = "_")

      png(file=paste("Figures/Volcano_Plots/",
                     sort(unique(as.factor(IDs$Stage)))[i],
                     paste(sort(unique(as.factor(IDs$Species)))[SPECIES][1],
sort(unique(as.factor(IDs$Species)))[SPECIES][2], sep = "vs"),
                     ".png", sep = ""),
          type="cairo",
          units = "in",
          width = 6, height = 6,
          res = 300)
      volcanoplot(test2, col = ifelse(test3$adj.P.Val< 0.05, "red",
"#046C9A"))
      dev.off()


      RES[[length(RES)+1]] <- results

    } # Ends loop over species to compare
  } #Closes the if statement to correct for stages that don't have potential
inter-species comparisons
}


hist(unlist(RES), main = "Adjusted p-values")
```

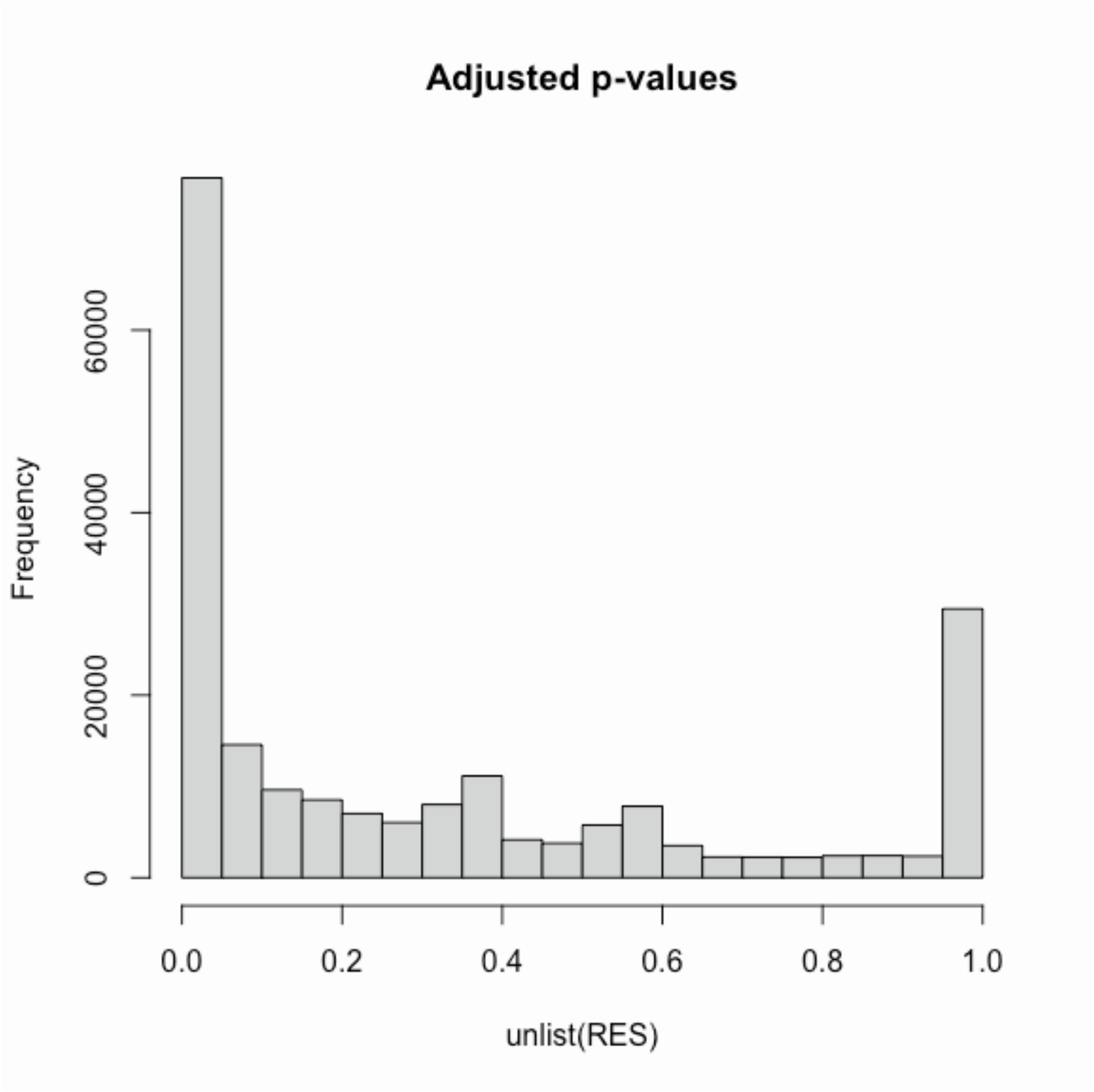


```
RESULTS <- do.call(cbind, RES)
row.names(RESULTS) <- NAMES
write.csv(RESULTS, file = "DEG_Analysis_InterSpecies_FINAL.csv", row.names =
TRUE)
pvalues <- RESULTS
REDUCED <- RESULTS[(row.names(RESULTS) %in% stringr::str_trim(KEEPERS[,1])),]
write.csv(REDUCED, file = "DEG_Analysis_InterSpecies_Reduced_FINAL.csv",
row.names = TRUE)


#Empty list to fill with resuts
RES <- list()

for(i in 1:length(unique(IDs$Stage))){
  STAGE <- c(i)
  design2 <- design[which(design[,2] == STAGE),]
```

```r
  DATA_Stage <- DATA[,which(design[,2] == STAGE)]

  if(length(unique(design2[,1]))>1){
    ##################################
    # Do all the pairwise comparisons of the different stages for each
collection type

    #The different combinations of species to test
    species_tests <- combn(unique(design2[,1]), 2)

    for(j in 1:ncol(species_tests)){
      SPECIES <- c(species_tests[,j])
      design3 <- design2[which(design2[,1] %in% SPECIES),]
      DATA_Stages <- DATA_Stage[,which(design2[,1] %in% SPECIES)]


      design4 <- design3[which(design3[,3] == 2 & design3[,1] == 1),]
      design4 <- rbind(design4, design3[which(design3[,3] == 1 & design3[,1]
== 2),])
      design4 <- rbind(design4, design3[which(design3[,3] == 2 & design3[,1]
== 3),])
      DATA_Stages <-
DATA_Stages[,as.numeric(row.names(plyr::match_df(as.data.frame(design3),
as.data.frame(design4))))]


      test <- lmFit(DATA_Stages, design = model.matrix(~ 1 + design4[,1]))
      test2 <- eBayes(test)
      test3 <- topTable(test2, number = length(test2$coefficients), sort.by =
"none")
      results <- data.frame(test3$logFC)
      names(results) <- paste(sort(unique(as.factor(IDs$Stage)))[i],

paste(sort(unique(as.factor(IDs$Species)))[SPECIES][1],
sort(unique(as.factor(IDs$Species)))[SPECIES][2], sep = "vs"),
                              sep = "_")

      png(file=paste("Figures/Volcano_Plots/",
                     sort(unique(as.factor(IDs$Stage)))[i],
                     paste(sort(unique(as.factor(IDs$Species)))[SPECIES][1],
sort(unique(as.factor(IDs$Species)))[SPECIES][2], sep = "vs"),
                     ".png", sep = ""),
          type="cairo",
          units = "in",
          width = 6, height = 6,
          res = 300)
      volcanoplot(test2, col = ifelse(test3$adj.P.Val< 0.05, "red",
"#046C9A"))
      dev.off()
```

```
      RES[[length(RES)+1]] <- results

    } # Ends loop over species to compare
  } #Closes the if statement to correct for stages that don't have potential
inter-species comparisons
}


RESULTS <- do.call(cbind, RES)
row.names(RESULTS) <- NAMES
write.csv(RESULTS, file = "DEG_Analysis_InterSpecies_logFC_FINAL.csv",
row.names = TRUE)
REDUCED <- RESULTS[(row.names(RESULTS) %in% stringr::str_trim(KEEPERS[,1])),]
write.csv(REDUCED, file =
"DEG_Analysis_InterSpecies_logFC_Reduced_FINAL.csv", row.names = TRUE)


# numbers of significant up and down regulated for each and volcano plots
REGULATION <- RESULTS[0,]

for(i in 1:ncol(RESULTS)){

  SIGS <- RESULTS[which(pvalues[,i] < 0.05),i]

  REGULATION[1,i] <- length(which(SIGS < 0))

  REGULATION[2,i] <- length(which(SIGS > 0))
}

row.names(REGULATION) <- c("down", "up")

write.csv(REGULATION, file = "InterSpecies_Number_Sig_FINAL.csv", row.names =
TRUE)
```

0.5 ROAST

We accessed information of all epigenetic and metabolic pathways for humans (Reactome terms: “epigenetic regulation of gene expression”, “metabolism”, “metabolism of proteins” and “metabolism of RNA”) from the Reactome pathways database version 74 (Jassal et al., 2020). Reactome pathways are arranged into several tiers, the Reactome term “epigenetic regulation of gene expression” (Reactome ID: R-HSA-212165.2), included curated pathways involving 122 genes; the Reactome term “metabolism” (Reactome ID: R-HSA-1430728.10) involved 2,210 genes; the curated pathways of the Reactome term “metabolism of proteins” (Reactome ID: R-HSA-392499.7) involved 2,095 genes; and the curated pathways of the Reactome term “metabolism of RNA” (Reactome ID: R-HSA-

8953854.4) involved 739 genes. The rotation gene set (ROAST) algorithm was used to perform self-contained gene set analysis of each metabolic pathway, for the different developmental stages and species (Wu et al., 2010).

```
RES <- list()

#Subset the data and create the appropriate design matrix
#Pick which species you're interested in cow = 1, human = 2, mouse = 3

for(i in 1:length(unique(IDs$Species))){
  SPECIES <- c(i)
  design2 <- design[which(design[,1] == SPECIES),]
  DATA_Species <- DATA[,which(design[,1] == SPECIES)]

  #################################
  # Do all the pairwise comparisons of the different stages for each
collection type

  #The different combinations of stages to test
  stage_tests <- combn(unique(design2[,2]), 2)

  #Loop over the combinations of stage(s) 16C = 1; 2C = 2; 4C = 3; 8C = 4; BL
= 5; MII = 6; MO = 7
  for(j in 1:ncol(stage_tests)){
    STAGES <- c(stage_tests[,j])
    design3 <- design2[which(design2[,2] %in% STAGES),]
    DATA_Stages <- DATA_Species[,which(design2[,2] %in% STAGES)]

    #Added this because humans don't have in vivo
    if(SPECIES != 2){
      #Loop over collection type(s): vitro = 1; vivo = 2
      for(k in 1:2){
        COLLECTION <- k
        design4 <- design3[which(design3[,3] %in% COLLECTION),]
        DATA_test <- DATA_Stages[,which(design3[,3] %in% COLLECTION)]

        if(length(unique(design4[,2])) > 1){
          # Loop over the different pathways that are going to be tested
          for(l in 1:ncol(GENES)){
            #Select genes in the pathway
            Index <- which(NAMES %in% GENES[,l])

            #Conduct the test (REMEBER TO CHANGE CONTRAST 1 = species, 2 =
stages, 3 = collection type)
            ROAST <- mroast(DATA_test,Index,design4[,1:2],contrast=2)
            ROAST$Stages <-
paste(sort(unique(as.factor(IDs$Stage)))[STAGES][1],
sort(unique(as.factor(IDs$Stage)))[STAGES][2], sep = "_")
            ROAST$Collection <- sort(unique(IDs$Collection))[k]
```

```
            ROAST$Pathway <- names(GENES)[l]
            ROAST$Species <- unique(IDs$Species)[i]

            #Save the results
            RES[[length(RES)+1]] <- ROAST

          } }#Closes the loop over the different pathways
      } #Closes the loop over collection type
    } else {

      #Loop over collection type(s): vitro = 1; vivo = 2
      for(k in 1){
        COLLECTION <- k
        design4 <- design3[which(design3[,3] %in% COLLECTION),]
        DATA_test <- DATA_Stages[,which(design3[,3] %in% COLLECTION)]

        # Loop over the different pathways that are going to be tested
        for(l in 1:ncol(GENES)){
          #Select genes in the pathway
          Index <- which(NAMES %in% GENES[,l])

          #Conduct the test (REMEBER TO CHANGE CONTRAST 1 = species, 2 =
stages, 3 = collection type)
          ROAST <- mroast(DATA_test,Index,design4[,1:2],contrast=2)
          ROAST$Stages <-
paste(sort(unique(as.factor(IDs$Stage)))[STAGES][1],
sort(unique(as.factor(IDs$Stage)))[STAGES][2], sep = "_")
          ROAST$Collection <- rev(unique(IDs$Collection))[k]
          ROAST$Pathway <- names(GENES)[l]
          ROAST$Species <- unique(IDs$Species)[i]

          #Save the results
          RES[[length(RES)+1]] <- ROAST

        } #Closes the loop over the different pathways
      } #Closes the loop over collection type
    } # Closes the else from the ifelse
  } #Closes the loop over the stage types

  #################################
  # Do all the pairwise comparisons of the different stages for each
collection type

  #Ignore these these for humans
  if(SPECIES != 2){
    #Loop over the combinations of stage(s) 16C = 1; 2C = 2; 4C = 3; 8C = 4;
BL = 5; MII = 6; MO = 7
    for(m in 1:length(unique(design2[,2]))){
      STAGES <- unique(design2[,2])[m]
```

```
      design3 <- design2[which(design2[,2] %in% STAGES),]
      DATA_Stages <- DATA_Species[,which(design2[,2] %in% STAGES)]

      if(length(unique(design3[,3])) > 1){
        # Loop over the different pathways that are going to be tested
        for(n in 1:ncol(GENES)){
          #Select genes in the pathway
          Index <- which(NAMES %in% GENES[,n])

          #Conduct the test (REMEBER TO CHANGE CONTRAST 1 = species, 2 =
stages, 3 = collection type)
          ROAST <- mroast(DATA_Stages,Index,design3[,c(1,3)],contrast=3)

          ROAST$Stages <- sort(unique(as.factor(IDs$Stage)))[STAGES]
          ROAST$Collection <- "vitro - vivo"
          ROAST$Pathway <- names(GENES)[n]
          ROAST$Species <- unique(IDs$Species)[i]

          #Save the results
          RES[[length(RES)+1]] <- ROAST

        } } #Closes the loop over the different pathways
    } #Closes the loop over the stage types
  } #Closes the if statement
} #Closes the top level loop

test <- do.call(rbind, RES)

write.csv(test, file = "Roast_FINAL.csv", row.names = FALSE)
```

0.6 Expression Figures

The following script recreates the expression pannels shown in Figure 4 in the main text.

```
IDs_2 <- read.csv("all_extra2.csv")

for(i in 1:length(NAMES)){
  MEANS <- aggregate(DATA[i,], by = IDs_2, FUN = "mean")
  CI <- function(x) sqrt(var(x)/length(x))*1.96
  CIs <- aggregate(DATA[i,], by = IDs_2, FUN = "CI")
  MEANS$CIs <- CIs$x
  MEANS <- MEANS[-which(MEANS$Species == "cow" & MEANS$Collection ==
"vitro"),]
  MEANS <- MEANS[-which(MEANS$Species == "mouse" & MEANS$Collection ==
"vitro"),]

  path <- paste("Figures/Expression_Figures/", NAMES[i], "_Expression.png",
sep = "")
```

```r
  YLAB <- paste(NAMES[i], " Adjusted expression", sep = "")

  FIG <-
    ggplot(MEANS, aes(x=Stage, y=x, color = Species), guide = FALSE) +
    geom_line(size = 1.5) +
    geom_errorbar(aes(ymin=x-CIs, ymax=x+CIs), width=.1) +
    theme_bw() +
    scale_color_manual(labels=c("Bovine", "Human", "Mouse"), values =
c("#e6c141", "#8a3bb8", "#3c7a47")) +
    ylab(YLAB) +
    xlab("Stage") +
    theme(panel.grid.major = element_blank(),
          panel.border = element_rect(colour = "black", size=1),
          panel.grid.minor = element_blank(),
          axis.title.x  = element_blank(),
          axis.title.y  = element_text(size=10),
          plot.title = element_text(size=8, hjust = 0),
          axis.text.y  = element_text(size=5),
          axis.text.x  = element_text(size=7),
          legend.position="top",
          legend.background = element_blank(),
          legend.title = element_blank(),
          legend.text = element_text(size=8),
          legend.key.size = unit(0.3, "cm"),
          legend.key = element_blank(),
          panel.background = element_rect(fill = "transparent"),
          plot.background = element_rect(fill = "transparent", color = NA)) +
    scale_x_continuous(breaks = 1:6, labels = c("MII", "2C", "4C", "8C",
"16C/MO", "BL"))

  ggsave(FIG,
         file=path,
         bg = "transparent",
         width = 3.23,
         height=3,
         units = "in",
         dpi = 600)
}


for(i in 1:length(NAMES)){
  MEANS <- aggregate(DATA[i,], by = IDs_2, FUN = "mean")
  CI <- function(x) sqrt(var(x)/length(x))*1.96
  CIs <- aggregate(DATA[i,], by = IDs_2, FUN = "CI")
  MEANS$CIs <- CIs$x

  path <- paste("Figures/Expression_Figures_2/", NAMES[i], "_Expression.png",
sep = "")
```

```
  YLAB <- paste(NAMES[i], " Adjusted expression", sep = "")

  FIG <-
    ggplot(MEANS, aes(x=Stage, y=x, color = Species), guide = FALSE) +
    geom_line(size = 1, aes(linetype = Collection)) +
    geom_errorbar(aes(ymin=x-CIs, ymax=x+CIs), width=.1) +
    theme_bw() +
    scale_color_manual(labels=c("Bovine", "Human", "Mouse"), values =
c("#e6c141", "#8a3bb8", "#3c7a47")) +
    scale_linetype_manual(values = c("dashed", "solid"), guide = FALSE) +
    ylab(YLAB) +
    xlab("Stage") +
    theme(panel.grid.major = element_blank(),
          panel.border = element_rect(colour = "black", size=1),
          panel.grid.minor = element_blank(),
          axis.title.x  = element_blank(),
          axis.title.y  = element_text(size=10),
          plot.title = element_text(size=8, hjust = 0),
          axis.text.y  = element_text(size=5),
          axis.text.x  = element_text(size=7),
          legend.position="top",
          legend.background = element_blank(),
          legend.title = element_blank(),
          legend.text = element_text(size=8),
          legend.key.size = unit(0.3, "cm"),
          legend.key = element_blank(),
          panel.background = element_rect(fill = "transparent"),
          plot.background = element_rect(fill = "transparent", color = NA)) +
    scale_x_continuous(breaks = 1:6, labels = c("MII", "2C", "4C", "8C",
"16C/MO", "BL"))

  ggsave(FIG,
         file=path,
         bg = "transparent",
         width = 3.23,
         height=3,
         units = "in",
         dpi = 600)
}
```

0.7 Correlations Between Methylated Genes and Gene Expression

```
#Load in the packages
library(ggplot2)
library(viridisLite)
library(gridExtra)

################################################################
# Data import and preparation
```

```r
###############################################################

#Load in the raw RNA Seq data data
data <- read.csv("All_Merged_Selected.csv")
data <- data[1:13134,]
SPECIES <- data[1,-1]
GROUP <- data[2,-1]
NAMES <- data[-c(1:2),1]
data <- data.frame(t(data[-c(1:2),-1]))

data <- as.data.frame(do.call(cbind,lapply(1:ncol(data), function(t)
  data[,t] <- as.numeric(data[,t])
)))

colnames(data) <- NAMES

#Load in the meta data
IDs <- na.omit(read.csv("all_extra.csv"))


# PQN Normalisation of the data

#Calculate the median of each gene's expression to generate a reference
sample
ref <- apply(data, 2, median)

for(i in 1:nrow(data)){
  QUOTIENTS <- data[i,]/ref
  m_j <- median(t(QUOTIENTS))
  data[i,] <- data[i,]/m_j
  data[i,][data[i,] == min(data[i,])] <- 0
}

# Split into individual datasets for each species/stage
DATA <- data
names(DATA) <- make.names(names(DATA))
#Subset only the most important genes.
SELECTED <- read.csv("genes_selected 2.csv")
DATA <- DATA[,which(names(DATA) %in% SELECTED$Genes)]

DATA$Stage <- factor(IDs$Stage, ordered = TRUE, levels = c("MII", "2C", "4C",
"8C", "16C", "MO", "BL"))
DATA$Species <- as.factor(IDs$Species)

#Parse the data out by species and collection type
cow_vitro_data <- DATA[which(IDs$Collection == "vitro" & IDs$Species ==
"cow"),]
cow_vivo_data <- DATA[which(IDs$Collection == "vivo" & IDs$Species ==
"cow"),]
```

```r
mouse_vitro_data <- DATA[which(IDs$Collection == "vitro" & IDs$Species ==
"mouse"),]
mouse_vivo_data <- DATA[which(IDs$Collection == "vivo" & IDs$Species ==
"mouse"),]

human_vitro_data <- DATA[which(IDs$Collection == "vitro" & IDs$Species ==
"human"),]


##################
# import and subset the methylation data
meth_data <- read.csv('methylation_data_2.csv')
meth_data$Stage <- factor(meth_data$phase, ordered = TRUE, levels = c("MII",
"2C", "4C", "8C", "16C", "MO", "BL"))
meth_data$meth_scaled <- (meth_data$Methylation/100)*4 - 2

##################
# Bovine in vitro
cow_vitro_methylation <- meth_data[which(meth_data$condition == "vitro" &
meth_data$species == "b"),]
# mean methylation values
cow_vitro_methylation <- aggregate(meth_scaled ~ Stage,
                                   data = cow_vitro_methylation,
                                   FUN = 'mean')

# convert to ordered factor for plotting
cow_vitro_methylation$Stage <- factor(cow_vitro_methylation$Stage,
                                      ordered = TRUE,
                                      levels = c("MII","2C","4C","8C",
                                                 "16C","MO","BL"))

cow_vitro_methylation <- cow_vitro_methylation[-
which(cow_vitro_methylation$Stage == "MO"),]

##################
# Bovine in vivo
cow_vivo_methylation <- meth_data[which(meth_data$condition == "vivo" &
meth_data$species == "b"),]

# mean methylation values
cow_vivo_methylation <- aggregate(meth_scaled ~ Stage,
                                  data = cow_vivo_methylation,
                                  FUN = 'mean')

# convert to ordered factor for plotting
cow_vivo_methylation$Stage <- factor(cow_vivo_methylation$Stage,
                                     ordered = TRUE,
                                     levels = c("MII","2C","4C","8C",
```

```
                                                    "16C","MO","BL"))

##################
# Mouse in vitro
mouse_vitro_methylation <- meth_data[which(meth_data$condition == "vitro" &
meth_data$species == "m"),]

# mean methylation values
mouse_vitro_methylation <- aggregate(meth_scaled ~ Stage,
                                     data = mouse_vitro_methylation,
                                     FUN = 'mean')

# convert to ordered factor for plotting
mouse_vitro_methylation$Stage <- factor(mouse_vitro_methylation$Stage,
                                        ordered = TRUE,
                                        levels = c("MII","2C","4C","8C",
                                                   "16C","MO","BL"))

##################
# Mouse in vivo
mouse_vivo_methylation <- meth_data[which(meth_data$condition == "vivo" &
meth_data$species == "m"),]

# mean methylation values
mouse_vivo_methylation <- aggregate(meth_scaled ~ Stage,
                                    data = mouse_vivo_methylation,
                                    FUN = 'mean')

# convert to ordered factor for plotting
mouse_vivo_methylation$Stage <- factor(mouse_vivo_methylation$Stage,
                                       ordered = TRUE,
                                       levels = c("MII","2C","4C","8C",
                                                  "16C","MO","BL"))

##################
# Human in vitro
human_vitro_methylation <- meth_data[which(meth_data$condition == "vitro" &
meth_data$species == "h"),]

# mean methylation values
human_vitro_methylation <- aggregate(meth_scaled ~ Stage,
                                     data = human_vitro_methylation,
                                     FUN = 'mean')

# convert to ordered factor for plotting
human_vitro_methylation$Stage <- factor(human_vitro_methylation$Stage,
                                        ordered = TRUE,
                                        levels = c("MII","2C","4C","8C",
                                                   "16C","MO","BL"))
```

```
###########################################
# Bovine analyses
###########################################

# Bovine Vitro
cow_vitro_data$Species <- NULL

# Stage means across all the genes
MEANS <- aggregate(. ~ Stage, data =cow_vitro_data, FUN = "mean")
ALL_MEANS <- data.frame(Stage = MEANS[,1],
                        Expression = rowMeans(MEANS[,2:ncol(MEANS)]))
ALL_MEANS$Expression <- scale(ALL_MEANS$Expression, center = TRUE, scale =
TRUE)


RES <- list()
COR <- list()
for(i in 1:103){
  MEANS <- aggregate(cow_vitro_data[,i] ~ cow_vitro_data$Stage, FUN = "mean")
  names(MEANS) <- c("Stage","Expression")
  MEANS$Gene <- names(cow_vitro_data)[i]
  MEANS$Expression <- scale(MEANS$Expression, center = TRUE, scale = TRUE)
  RES[[i]] <- MEANS

  # correlations
  EXP <- data.frame(expression = cow_vitro_data[,i], Stage =
cow_vitro_data$Stage)
  MERGE <- merge(EXP, cow_vitro_methylation, by.x = 'Stage', by.y = 'Stage')

  COR[[i]] <- data.frame(Gene = names(cow_vitro_data)[i],
                         rho = cor(MERGE$expression, MERGE$meth_scaled),
                         source = 'cow_vitro')
}

COR_cow_vitro <- do.call(rbind, COR)

RES <- do.call(rbind, RES)
RES[is.na(RES)] <- 0

ALL_MEANS <- aggregate(Expression ~ Stage, data =RES, FUN = "mean")
names(ALL_MEANS)[2] <- "Expression"

cow_vitro <-
  ggplot(RES, aes(x=Stage, y=Expression, fill = Gene, group = Gene), color =
'grey70', guide = FALSE) +
  stat_summary(fun=sum, geom="line", size = 0.2, alpha = 0.4, color =
'grey70') +
  geom_line(data = cow_vitro_methylation, aes(x=Stage, y=meth_scaled, fill =
```

```
'1', group = '1'), size = 1, col = "#e6c141", linetype = 'dashed') +
  geom_line(data = ALL_MEANS, aes(x=Stage, y=Expression, fill = '1', group =
'1'), size = 1, col = "grey30", linetype = 'dashed') +
  theme_bw() +
  ylab('Scaled expression values') +
  xlab("Stage") +
  theme(panel.grid.major = element_blank(),
        panel.border = element_rect(colour = "black", size=1),
        panel.grid.minor = element_blank(),
        axis.title.x  = element_blank(),
        axis.title.y  = element_text(size=10),
        plot.title = element_text(size=8, hjust = 0),
        axis.text.y  = element_text(size=5),
        axis.text.x  = element_text(size=7),
        legend.position="top",
        legend.background = element_blank(),
        legend.title = element_blank(),
        legend.text = element_text(size=8),
        legend.key.size = unit(0.3, "cm"),
        legend.key = element_blank(),
        panel.background = element_rect(fill = "transparent"),
        plot.background = element_rect(fill = "transparent", color = NA)) +
  scale_y_continuous(limits = c(-2.2,2.2),
                     sec.axis = sec_axis(~ (. + 2)/4*100, name = "Percent
Methylation")) +
  scale_x_discrete(expand = c(0,0))


# Cow Vivo

# Stage means across all the genes
cow_vivo_data$Species <- NULL
MEANS <- aggregate(. ~ Stage, data =cow_vivo_data, FUN = "mean")
ALL_MEANS <- data.frame(Stage = MEANS[,1],
                        Expression = rowMeans(MEANS[,2:ncol(MEANS)]))
ALL_MEANS$Expression <- scale(ALL_MEANS$Expression, center = TRUE, scale =
TRUE)

RES <- list()
COR <- list()
for(i in 1:103){
  MEANS <- aggregate(cow_vivo_data[,i] ~ cow_vivo_data$Stage, FUN = "mean")
  names(MEANS) <- c("Stage","Expression")
  MEANS$Gene <- names(cow_vivo_data)[i]
  MEANS$Expression <- scale(MEANS$Expression, center = TRUE, scale = TRUE)
  RES[[i]] <- MEANS

  # correlations
  EXP <- data.frame(expression = cow_vivo_data[,i], Stage =
```

```
cow_vivo_data$Stage)
  MERGE <- merge(EXP, cow_vivo_methylation, by.x = 'Stage', by.y = 'Stage')

  COR[[i]] <- data.frame(Gene = names(cow_vivo_data)[i],
                         rho = cor(MERGE$expression, MERGE$meth_scaled),
                         source = 'cow_vivo')
}

COR_cow_vivo <- do.call(rbind, COR)

RES <- do.call(rbind, RES)
RES[is.na(RES)] <- 0

ALL_MEANS <- aggregate(Expression ~ Stage, data =RES, FUN = "mean")
names(ALL_MEANS)[2] <- "Expression"

cow_vivo <-
  ggplot(RES, aes(x=Stage, y=Expression, fill = Gene, group = Gene),
         color = 'grey70', guide = FALSE) +
  stat_summary(fun=sum, geom="line", size = 0.2, alpha = 0.4, color =
'grey70') +
  geom_line(data = cow_vivo_methylation, aes(x=Stage, y=meth_scaled, fill =
'1', group = '1'), size = 1, col = "#e6c141") +
  geom_line(data = ALL_MEANS, aes(x=Stage, y=Expression, fill = '1', group =
'1'), size = 1, col = "grey30") +
  theme_bw() +
  ylab('Scaled expression values') +
  xlab("Stage") +
  theme(panel.grid.major = element_blank(),
        panel.border = element_rect(colour = "black", size=1),
        panel.grid.minor = element_blank(),
        axis.title.x  = element_blank(),
        axis.title.y  = element_text(size=10),
        plot.title = element_text(size=8, hjust = 0),
        axis.text.y  = element_text(size=5),
        axis.text.x  = element_text(size=7),
        legend.position="top",
        legend.background = element_blank(),
        legend.title = element_blank(),
        legend.text = element_text(size=8),
        legend.key.size = unit(0.3, "cm"),
        legend.key = element_blank(),
        panel.background = element_rect(fill = "transparent"),
        plot.background = element_rect(fill = "transparent", color = NA)) +
  scale_y_continuous(limits = c(-2.2,2.2),
                     sec.axis = sec_axis(~ (. + 2)/4*100, name = "Percent
Methylation")) +
  scale_x_discrete(expand = c(0,0))
```

```r
############################################
# Mice
############################################

# Mouse Vitro

# Stage means across all the genes
mouse_vitro_data$Species <- NULL
MEANS <- aggregate(. ~ Stage, data =mouse_vitro_data, FUN = "mean")
ALL_MEANS <- data.frame(Stage = MEANS[,1],
                        Expression = rowMeans(MEANS[,2:ncol(MEANS)]))
ALL_MEANS$Expression <- scale(ALL_MEANS$Expression, center = TRUE, scale =
TRUE)

RES <- list()
COR <- list()
for(i in 1:103){
  MEANS <- aggregate(mouse_vitro_data[,i] ~ mouse_vitro_data$Stage, FUN =
"mean")
  names(MEANS) <- c("Stage","Expression")
  MEANS$Gene <- names(mouse_vitro_data)[i]
  MEANS$Expression <- scale(MEANS$Expression, center = TRUE, scale = TRUE)
  RES[[i]] <- MEANS

  # correlations
  EXP <- data.frame(expression = mouse_vitro_data[,i], Stage =
mouse_vitro_data$Stage)
  MERGE <- merge(EXP, mouse_vitro_methylation, by.x = 'Stage', by.y =
'Stage')

  COR[[i]] <- data.frame(Gene = names(mouse_vitro_data)[i],
                         rho = cor(MERGE$expression, MERGE$meth_scaled),
                         source = 'mouse_vitro')
}

COR_mouse_vitro <- do.call(rbind, COR)

RES <- do.call(rbind, RES)
RES[is.na(RES)] <- 0

ALL_MEANS <- aggregate(Expression ~ Stage, data =RES, FUN = "mean")
names(ALL_MEANS)[2] <- "Expression"

mouse_vitro <-
  ggplot(RES, aes(x=Stage, y=Expression, fill = Gene, group = Gene), color =
'grey70', guide = FALSE) +
  stat_summary(fun=sum, geom="line", size = 0.2, alpha = 0.4, color =
'grey70') +
```

```
  geom_line(data = mouse_vitro_methylation, aes(x=Stage, y=meth_scaled, fill
= '1', group = '1'), size = 1, col = "#3c7a47", linetype = 'dashed') +
  geom_line(data = ALL_MEANS, aes(x=Stage, y=Expression, fill = '1', group =
'1'), size = 1, col = "grey30", linetype = 'dashed') +
  theme_bw() +
  ylab('Scaled expression values') +
  xlab("Stage") +
  theme(panel.grid.major = element_blank(),
        panel.border = element_rect(colour = "black", size=1),
        panel.grid.minor = element_blank(),
        axis.title.x  = element_blank(),
        axis.title.y  = element_text(size=10),
        plot.title = element_text(size=8, hjust = 0),
        axis.text.y  = element_text(size=5),
        axis.text.x  = element_text(size=7),
        legend.position="top",
        legend.background = element_blank(),
        legend.title = element_blank(),
        legend.text = element_text(size=8),
        legend.key.size = unit(0.3, "cm"),
        legend.key = element_blank(),
        panel.background = element_rect(fill = "transparent"),
        plot.background = element_rect(fill = "transparent", color = NA)) +
  scale_y_continuous(limits = c(-2.2,2.2),
                     sec.axis = sec_axis(~ (. + 2)/4*100, name = "Percent
Methylation")) +
  scale_x_discrete(expand = c(0,0))


# Mouse Vivo

# Stage means across all the genes
mouse_vivo_data$Species <- NULL
MEANS <- aggregate(. ~ Stage, data =mouse_vivo_data, FUN = "mean")
ALL_MEANS <- data.frame(Stage = MEANS[,1],
                        Expression = rowMeans(MEANS[,2:ncol(MEANS)]))
ALL_MEANS$Expression <- scale(ALL_MEANS$Expression, center = TRUE, scale =
TRUE)

RES <- list()
COR <- list()
for(i in 1:103){
  MEANS <- aggregate(mouse_vivo_data[,i] ~ mouse_vivo_data$Stage, FUN =
"mean")
  names(MEANS) <- c("Stage","Expression")
  MEANS$Gene <- names(mouse_vivo_data)[i]
  MEANS$Expression <- scale(MEANS$Expression, center = TRUE, scale = TRUE)
  RES[[i]] <- MEANS
```

```
  # correlations
  EXP <- data.frame(expression = mouse_vivo_data[,i], Stage =
mouse_vivo_data$Stage)
  MERGE <- merge(EXP, mouse_vivo_methylation, by.x = 'Stage', by.y = 'Stage')

  COR[[i]] <- data.frame(Gene = names(mouse_vivo_data)[i],
                         rho = cor(MERGE$expression, MERGE$meth_scaled),
                         source = 'mouse_vivo')
}

COR_mouse_vivo <- do.call(rbind, COR)

RES <- do.call(rbind, RES)
RES[is.na(RES)] <- 0

ALL_MEANS <- aggregate(Expression ~ Stage, data =RES, FUN = "mean")
names(ALL_MEANS)[2] <- "Expression"

mouse_vivo <-
  ggplot(RES, aes(x=Stage, y=Expression, fill = Gene, group = Gene), color =
'grey70', guide = FALSE) +
  stat_summary(fun=sum, geom="line", size = 0.2, alpha = 0.4, color =
'grey70') +
  geom_line(data = mouse_vivo_methylation, aes(x=Stage, y=meth_scaled, fill =
'1', group = '1'), size = 1, col = "#3c7a47") +
  geom_line(data = ALL_MEANS, aes(x=Stage, y=Expression, fill = '1', group =
'1'), size = 1, col = "grey30") +
  theme_bw() +
  ylab('Scaled expression values') +
  xlab("Stage") +
  theme(panel.grid.major = element_blank(),
        panel.border = element_rect(colour = "black", size=1),
        panel.grid.minor = element_blank(),
        axis.title.x  = element_blank(),
        axis.title.y  = element_text(size=10),
        plot.title = element_text(size=8, hjust = 0),
        axis.text.y  = element_text(size=5),
        axis.text.x  = element_text(size=7),
        legend.position="top",
        legend.background = element_blank(),
        legend.title = element_blank(),
        legend.text = element_text(size=8),
        legend.key.size = unit(0.3, "cm"),
        legend.key = element_blank(),
        panel.background = element_rect(fill = "transparent"),
        plot.background = element_rect(fill = "transparent", color = NA)) +
  scale_y_continuous(limits = c(-2.2,2.2),
                     sec.axis = sec_axis(~ (. + 2)/4*100, name = "Percent
Methylation")) +
```

```r
  scale_x_discrete(expand = c(0,0))


###########################################
# Humans
###########################################

# Human Vitro

# Stage means across all the genes
human_vitro_data$Species <- NULL
MEANS <- aggregate(. ~ Stage, data =human_vitro_data, FUN = "mean")
ALL_MEANS <- data.frame(Stage = MEANS[,1],
                        Expression = rowMeans(MEANS[,2:ncol(MEANS)]))
ALL_MEANS$Expression <- scale(ALL_MEANS$Expression, center = TRUE, scale =
TRUE)

RES <- list()
COR <- list()
for(i in 1:103){
  MEANS <- aggregate(human_vitro_data[,i] ~ human_vitro_data$Stage, FUN =
"mean")
  names(MEANS) <- c("Stage","Expression")
  MEANS$Gene <- names(human_vitro_data)[i]
  MEANS$Expression <- scale(MEANS$Expression, center = TRUE, scale = TRUE)
  RES[[i]] <- MEANS

  # correlations
  EXP <- data.frame(expression = human_vitro_data[,i], Stage =
human_vitro_data$Stage)
  MERGE <- merge(EXP, human_vitro_methylation, by.x = 'Stage', by.y =
'Stage')

  COR[[i]] <- data.frame(Gene = names(human_vitro_data)[i],
                         rho = cor(MERGE$expression, MERGE$meth_scaled),
                         source = 'human_vitro')
}

COR_human_vitro <- do.call(rbind, COR)

RES <- do.call(rbind, RES)
RES[is.na(RES)] <- 0

ALL_MEANS <- aggregate(Expression ~ Stage, data =RES, FUN = "mean")
names(ALL_MEANS)[2] <- "Expression"

human_vitro <-
  ggplot(RES, aes(x=Stage, y=Expression, fill = Gene, group = Gene), color =
'grey70', guide = FALSE) +
```

```
  stat_summary(fun=sum, geom="line", size = 0.2, alpha = 0.4, color = 
'grey70') +
  geom_line(data = human_vitro_methylation, aes(x=Stage, y=meth_scaled, fill 
= '1', group = '1'), size = 1, col = "#8a3bb8", linetype = 'dashed') +
  geom_line(data = ALL_MEANS, aes(x=Stage, y=Expression, fill = '1', group = 
'1'), size = 1, col = "grey30", linetype = 'dashed') +
  theme_bw() +
  ylab('Scaled expression values') +
  xlab("Stage") +
  theme(panel.grid.major = element_blank(),
        panel.border = element_rect(colour = "black", size=1),
        panel.grid.minor = element_blank(),
        axis.title.x  = element_blank(),
        axis.title.y  = element_text(size=10),
        plot.title = element_text(size=8, hjust = 0),
        axis.text.y  = element_text(size=5),
        axis.text.x  = element_text(size=7),
        legend.position="top",
        legend.background = element_blank(),
        legend.title = element_blank(),
        legend.text = element_text(size=8),
        legend.key.size = unit(0.3, "cm"),
        legend.key = element_blank(),
        panel.background = element_rect(fill = "transparent"),
        plot.background = element_rect(fill = "transparent", color = NA)) +
  scale_y_continuous(limits = c(-2.2,2.2),
                     sec.axis = sec_axis(~ (. + 2)/4*100, name = "Percent 
Methylation")) +
  scale_x_discrete(expand = c(0,0))



grid.arrange(cow_vivo, cow_vitro, mouse_vivo, mouse_vitro, human_vitro, ncol 
= 1)
```

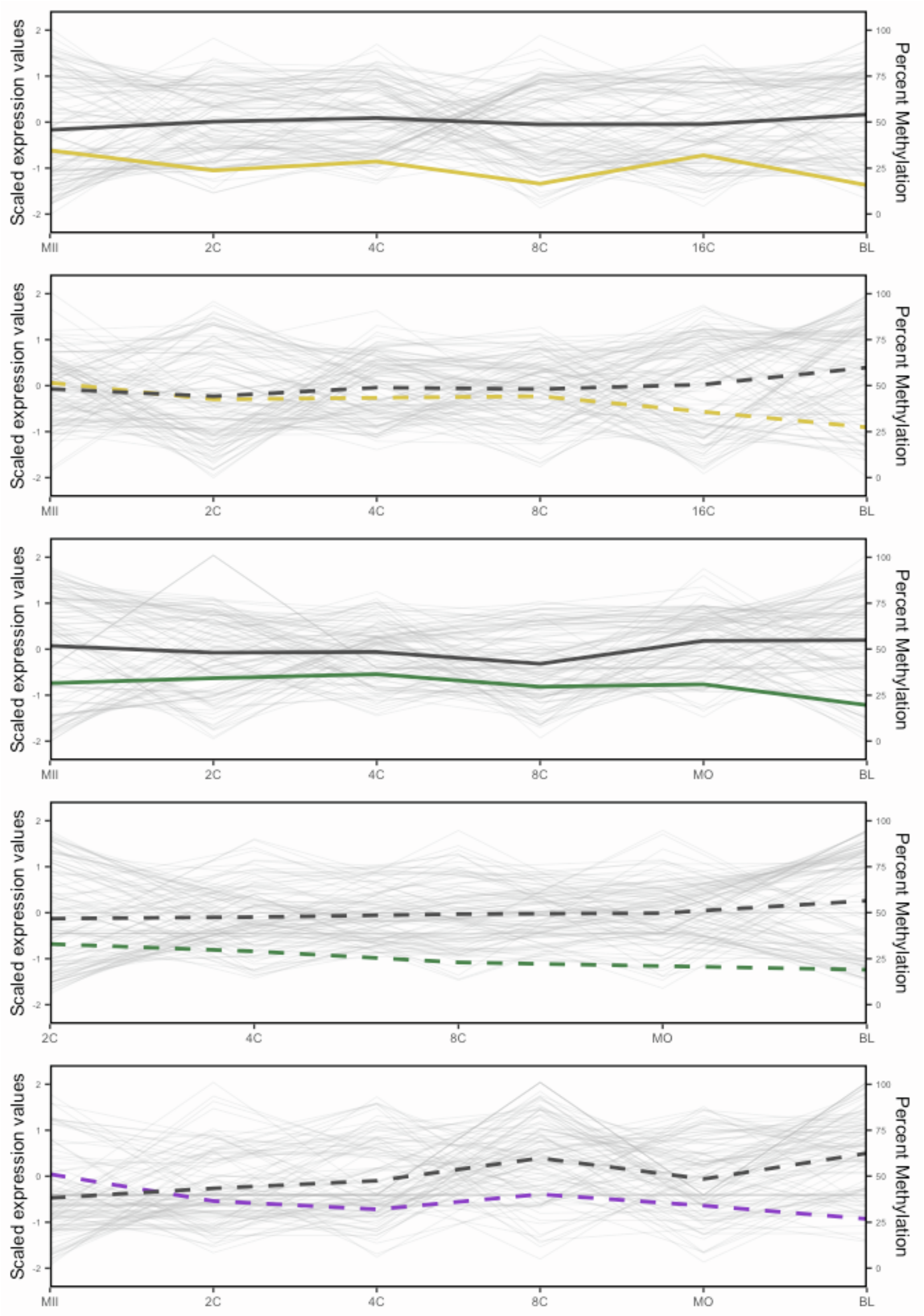

Scaled expression values
Percent Methylation
2
1
0
-1
-2
100
75
50
25
0
MII
2C
4C
8C
16C
BL
Scaled expression values
Percent Methylation
MII
2C
4C
8C
16C
BL
Scaled expression values
Percent Methylation
MII
2C
4C
8C
MO
BL
Scaled expression values
Percent Methylation
2C
4C
8C
MO
BL
Scaled expression values
Percent Methylation
MII
2C
4C
8C
MO
BL


```
#############################
# Heatmap
```

```r
#Data for correlation heatmap

correlations <- do.call(rbind, list(COR_cow_vivo, COR_cow_vitro,
COR_mouse_vivo,COR_mouse_vitro,COR_human_vitro))

correlations$source <- factor(correlations$source, ordered = TRUE, levels =
c("cow_vivo", "cow_vitro", "mouse_vivo", "mouse_vitro", "human_vitro"))

#SELECTED <- read.csv("genes_selected.csv")
#correlations <- correlations[which(correlations$Gene %in% SELECTED$Genes),]

correlations[is.na(correlations)] <- 0

#Plot the results
  ggplot() +
  geom_tile(data = correlations, aes(x = source, y = Gene, fill = rho),
            color = "black", size = 0.01) +
  scale_fill_gradient2(low = "#008282", high = "#c53f27",
                       midpoint = 0, mid = "white",
                       na.value = "black", name = expression(rho),
                       limits=c(-1,1)) +
  ylab('') +
  xlab("") +
  theme_bw() +
  theme(legend.title = element_text(size = 10, family = "serif"),
        legend.text = element_text(size = 10, family = "serif"),
        legend.position="top",
        plot.title = element_text(size=16, family = "serif"),
        axis.title = element_text(size=12, family = "serif"),
        axis.text.y = element_text(size=7, family = "serif"),
        axis.text.x = element_text(size=5.5, family = "serif")) +
  scale_x_discrete(expand = c(0,0.1), breaks = c("cow_vivo", "cow_vitro",
                                                 "mouse_vivo", "mouse_vitro",
                                                 "human_vitro"),
                   labels = c("Bovine in vivo", "Bovine in vitro",
                              "Mouse in vivo", "Mouse in vitro",
                              "Human in vitro")) +
  scale_y_discrete(expand = c(0,0))
```

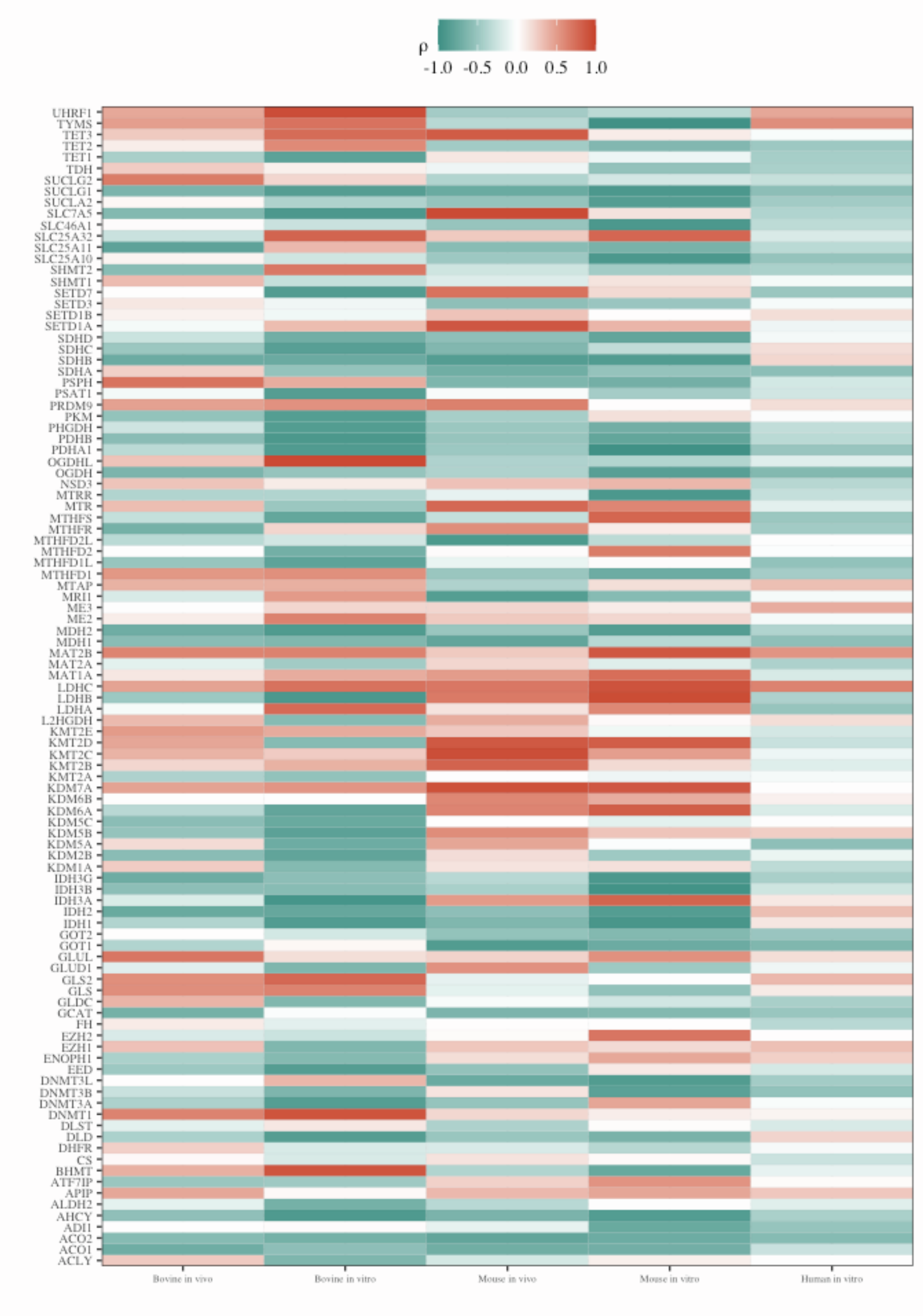

ρ
-1.0 -0.5 0.0 0.5 1.0
UHRF1
TYMS
TET3
TET2
TET1
TDH
SUCLG2
SUCLG1
SUCLA2
SLC7A5
SLC46A1
SLC25A32
SLC25A11
SLC25A10
SHMT2
SHMT1
SETD7
SETD3
SETD1B
SETD1A
SDHD
SDHC
SDHB
SDHA
PSPH
PSAT1
PRDM9
PKM
PHGDH
PDHB
PDHA1
OGDHL
OGDH
NSD3
MTRR
MTR
MTHFS
MTHFR
MTHFD2L
MTHFD2
MTHFD1L
MTHFD1
MTAP
MRI1
ME3
ME2
MDH2
MDH1
MAT2B
MAT2A
MAT1A
LDHC
LDHB
LDHA
L2HGDH
KMT2E
KMT2D
KMT2C
KMT2B
KMT2A
KDM7A
KDM6B
KDM6A
KDM5C
KDM5B
KDM5A
KDM2B
KDM1A
IDH3G
IDH3B
IDH3A
IDH2
IDH1
GOT2
GOT1
GLUL
GLUD1
GLS2
GLS
GLDC
GCAT
FH
EZH2
EZH1
ENOPH1
EED
DNMT3L
DNMT3B
DNMT3A
DNMT1
DLST
DLD
DHFR
CS
BHMT
ATF7IP
APIP
ALDH2
AHCY
ADI1
ACO2
ACO1
ACLY
Bovine in vivo
Bovine in vitro
Mouse in vivo
Mouse in vitro
Human in vitro

0.8 Correlations Between Methylated Genes and Reactome Pathways

```
#Import the roast information
DATA <- read.csv('Roast_FINAL.csv')

#Subset the correct data
DATA <- DATA[-which(DATA$Collection == "vitro - vivo"),]
DATA <- DATA[which(DATA$Stages == "MII_2C" | DATA$Stages == "2C_MII" |
                     DATA$Stages == "4C_2C" | DATA$Stages == "2C_4C" |
                     DATA$Stages == "4C_8C" | DATA$Stages == "8C_4C" |
                     DATA$Stages == "8C_16C" | DATA$Stages == "16C_8C" |
                     DATA$Stages == "MO_8C" | DATA$Stages == "8C_MO" |
                     DATA$Stages == "16C_BL" | DATA$Stages == "BL_16C" |
                     DATA$Stages == "MO_BL" | DATA$Stages == "BL_MO"),]

#line up the stages
DATA[which(DATA$Stages == "MII_2C" | DATA$Stages == "2C_MII"), 'Stages'] <-
"2C"
DATA[which(DATA$Stages == "4C_2C" | DATA$Stages == "2C_4C"), 'Stages'] <-
"4C"
DATA[which(DATA$Stages == "4C_8C" | DATA$Stages == "8C_4C"), 'Stages'] <-
"8C"
DATA[which(DATA$Stages == "8C_16C" | DATA$Stages == "16C_8C"), 'Stages'] <-
"16C"
DATA[which(DATA$Stages == "8C_MO" | DATA$Stages == "MO_8C"), 'Stages'] <-
"MO"
DATA[which(DATA$Stages == "16C_BL" | DATA$Stages == "BL_16C"), 'Stages'] <-
"BL"
DATA[which(DATA$Stages == "MO_BL" | DATA$Stages == "BL_MO"), 'Stages'] <-
"BL"

DATA$Stages <- factor(DATA$Stages, ordered = TRUE, levels = c("MII",
"MII_2C", "2C_MII", "2C", "2C_4C", "4C_2C", "4C","4C_8C", "8C_4C", "8C",
"8C_16C", "16C_8C", "16C", "16C_BL", "BL_16C", "MO", "MO_BL", "BL_MO", "BL"))



cow_vitro_data <- DATA[which(DATA$Collection == "vitro" & DATA$Species ==
"cow"),]
cow_vivo_data <- DATA[which(DATA$Collection == "vivo" & DATA$Species ==
"cow"),]

mouse_vitro_data <- DATA[which(DATA$Collection == "vitro" & DATA$Species ==
"mouse"),]
mouse_vivo_data <- DATA[which(DATA$Collection == "vivo" & DATA$Species ==
"mouse"),]

human_vitro_data <- DATA[which(DATA$Collection == "vitro" & DATA$Species ==
"human"),]
```

```
# import and subset the methylation data
meth_data <- read.csv('methylation_data_2.csv')
meth_data$Stage <- factor(meth_data$phase, ordered = TRUE, levels = c("MII",
"MII_2C", "2C_MII", "2C", "2C_4C", "4C_2C", "4C","4C_8C", "8C_4C", "8C",
"8C_16C", "16C_8C", "16C", "16C_BL", "BL_16C", "MO", "MO_BL", "BL_MO", "BL"))
meth_data$meth_scaled <- (meth_data$Methylation/100)*2 - 1

cow_vitro_methylation <- meth_data[which(meth_data$condition == "vitro" &
meth_data$species == "b"),]
cow_vivo_methylation <- meth_data[which(meth_data$condition == "vivo" &
meth_data$species == "b"),]

mouse_vitro_methylation <- meth_data[which(meth_data$condition == "vitro" &
meth_data$species == "m"),]
mouse_vivo_methylation <- meth_data[which(meth_data$condition == "vivo" &
meth_data$species == "m"),]

human_vitro_methylation <- meth_data[which(meth_data$condition == "vitro" &
meth_data$species == "h"),]

###########################################
# Cows
###########################################

# Cow Vitro

#Data wrangling
cow_vitro_data$Prop <- NA
for(i in 1:nrow(cow_vitro_data)){
  #Which direction
  if(cow_vitro_data[i,'Direction'] == 'Down'){
    cow_vitro_data$Prop[i] <- -cow_vitro_data[i,'PropDown']} else {
      cow_vitro_data$Prop[i] <- cow_vitro_data[i,'PropUp']
    }

  #Which direction
  if(cow_vitro_data[i,'Stages'] == '2C_MII' | cow_vitro_data[i,'Stages'] ==
'4C_2C' |
     cow_vitro_data[i,'Stages'] == '8C_4C' | cow_vitro_data[i,'Stages'] ==
'16C_8C' |
     cow_vitro_data[i,'Stages'] == "BL_16C"| cow_vitro_data[i,'Stages'] ==
'MO_BL'){

    if(cow_vitro_data[i,'Direction'] == 'Down'){
      cow_vitro_data$Prop[i] <- cow_vitro_data[i,'PropDown']} else {
        cow_vitro_data$Prop[i] <- -cow_vitro_data[i,'PropUp']
      }
  }
}
```

```r
# mean methylation values
cow_vitro_methylation <- aggregate(meth_scaled ~ Stage, data =
cow_vitro_methylation, FUN = 'mean')
cow_vitro_methylation$Stage <- factor(cow_vitro_methylation$Stage, ordered =
TRUE, levels = c("MII", "MII_2C", "2C_MII", "2C", "2C_4C", "4C_2C",
"4C","4C_8C", "8C_4C", "8C",              "8C_16C", "16C_8C", "16C", "16C_BL",
"BL_16C", "MO", "MO_BL", "BL_MO", "BL"))



PATHWAYS <- unique(cow_vitro_data$Pathway)
COR <- list()
for(i in 1:length(PATHWAYS)){
  PATH <- cow_vitro_data[which(cow_vitro_data$Pathway == PATHWAYS[i]),
c('Stages', 'Prop')]
  MERGE <- merge(PATH, cow_vitro_methylation, by.x = 'Stages', by.y =
'Stage')
  COR[[i]] <- data.frame(Pathway = PATHWAYS[i],
                         rho = cor(MERGE$Prop, MERGE$meth_scaled),
                         source = 'cow_vitro')
}
COR_cow_vitro <- do.call(rbind, COR)

# Stage means across all the genes
ALL_MEANS <- aggregate(Prop ~ Stages, data =cow_vitro_data, FUN = "mean")
ALL_MEANS$Prop_Scaled <- scale(ALL_MEANS$Prop, center = TRUE, scale = TRUE)

# # # # # # # # #
# Cow Vivo

#Data wrangling
cow_vivo_data$Prop <- NA
for(i in 1:nrow(cow_vivo_data)){
  #Which direction
  if(cow_vivo_data[i,'Direction'] == 'Down'){
    cow_vivo_data$Prop[i] <- -cow_vivo_data[i,'PropDown']} else {
      cow_vivo_data$Prop[i] <- cow_vivo_data[i,'PropUp']
    }

  #Which direction
  if(cow_vivo_data[i,'Stages'] == '2C_MII' | cow_vivo_data[i,'Stages'] ==
'4C_2C' |
     cow_vivo_data[i,'Stages'] == '8C_4C' | cow_vivo_data[i,'Stages'] ==
'16C_8C' |
     cow_vivo_data[i,'Stages'] == "BL_16C"| cow_vivo_data[i,'Stages'] ==
'MO_BL'){

    if(cow_vivo_data[i,'Direction'] == 'Down'){
```

```
      cow_vivo_data$Prop[i] <- cow_vivo_data[i,'PropDown']} else {
        cow_vivo_data$Prop[i] <- -cow_vivo_data[i,'PropUp']
      }
  }
}

# mean methylation values
cow_vivo_methylation <- aggregate(meth_scaled ~ Stage, data =
cow_vivo_methylation, FUN = 'mean')
cow_vivo_methylation$Stage <- factor(cow_vivo_methylation$Stage, ordered =
TRUE, levels = c("MII", "MII_2C", "2C_MII", "2C", "2C_4C", "4C_2C",
"4C","4C_8C", "8C_4C", "8C", "8C_16C", "16C_8C", "16C", "16C_BL", "BL_16C",
"MO", "MO_BL", "BL_MO", "BL"))


#Correlations between methylation and pathway up/down regulation
PATHWAYS <- unique(cow_vivo_data$Pathway)
COR <- list()
for(i in 1:length(PATHWAYS)){
  PATH <- cow_vivo_data[which(cow_vivo_data$Pathway == PATHWAYS[i]),
c('Stages', 'Prop')]
  MERGE <- merge(PATH, cow_vivo_methylation, by.x = 'Stages', by.y = 'Stage')
  COR[[i]] <- data.frame(Pathway = PATHWAYS[i],
                         rho = cor(MERGE$Prop, MERGE$meth_scaled),
                         source = 'cow_vivo')
}
COR_cow_vivo <- do.call(rbind, COR)


# Stage means across all the genes
ALL_MEANS <- aggregate(Prop ~ Stages, data =cow_vivo_data, FUN = "mean")
ALL_MEANS$Prop_Scaled <- scale(ALL_MEANS$Prop, center = TRUE, scale = TRUE)


###########################################
# Mice
###########################################

# Mouse Vitro

#Data wrangling
mouse_vitro_data$Prop <- NA
for(i in 1:nrow(mouse_vitro_data)){
  #Which direction
  if(mouse_vitro_data[i,'Direction'] == 'Down'){
    mouse_vitro_data$Prop[i] <- -mouse_vitro_data[i,'PropDown']} else {
      mouse_vitro_data$Prop[i] <- mouse_vitro_data[i,'PropUp']
    }
```

```
  #Which direction
  if(mouse_vitro_data[i,'Stages'] == '2C_MII' | mouse_vitro_data[i,'Stages']
== '4C_2C' |
     mouse_vitro_data[i,'Stages'] == '8C_4C' | mouse_vitro_data[i,'Stages']
== '16C_8C' |
     mouse_vitro_data[i,'Stages'] == "BL_16C"| mouse_vitro_data[i,'Stages']
== 'MO_BL'){


    if(mouse_vitro_data[i,'Direction'] == 'Down'){
      mouse_vitro_data$Prop[i] <- mouse_vitro_data[i,'PropDown']} else {
        mouse_vitro_data$Prop[i] <- -mouse_vitro_data[i,'PropUp']
      }
  }
}

# mean methylation values
mouse_vitro_methylation <- aggregate(meth_scaled ~ Stage, data =
mouse_vitro_methylation, FUN = 'mean')
mouse_vitro_methylation$Stage <- factor(mouse_vitro_methylation$Stage,
ordered = TRUE, levels = c("MII", "MII_2C", "2C_MII", "2C", "2C_4C", "4C_2C",
"4C","4C_8C", "8C_4C", "8C",              "8C_16C", "16C_8C", "16C",
"16C_BL", "BL_16C", "MO", "MO_BL", "BL_MO", "BL"))


#Correlations between methylation and pathway up/down regulation
PATHWAYS <- unique(mouse_vitro_data$Pathway)
COR <- list()
for(i in 1:length(PATHWAYS)){
  PATH <- mouse_vitro_data[which(mouse_vitro_data$Pathway == PATHWAYS[i]),
c('Stages', 'Prop')]
  MERGE <- merge(PATH, mouse_vitro_methylation, by.x = 'Stages', by.y =
'Stage')
  COR[[i]] <- data.frame(Pathway = PATHWAYS[i],
                         rho = cor(MERGE$Prop, MERGE$meth_scaled),
                         source = 'mouse_vitro')
}
COR_mouse_vitro <- do.call(rbind, COR)

# Stage means across all the genes
ALL_MEANS <- aggregate(Prop ~ Stages, data =mouse_vitro_data, FUN = "mean")
ALL_MEANS$Prop_Scaled <- scale(ALL_MEANS$Prop, center = TRUE, scale = TRUE)


# Mouse Vivo

#Data wrangling
mouse_vivo_data$Prop <- NA
for(i in 1:nrow(mouse_vivo_data)){
```

```
  #Which direction
  if(mouse_vivo_data[i,'Direction'] == 'Down'){
    mouse_vivo_data$Prop[i] <- -mouse_vivo_data[i,'PropDown']} else {
      mouse_vivo_data$Prop[i] <- mouse_vivo_data[i,'PropUp']
    }

  #Which direction
  if(mouse_vivo_data[i,'Stages'] == '2C_MII' | mouse_vivo_data[i,'Stages'] ==
'4C_2C' |
     mouse_vivo_data[i,'Stages'] == '8C_4C' | mouse_vivo_data[i,'Stages'] ==
'16C_8C' |
     mouse_vivo_data[i,'Stages'] == "BL_16C"| mouse_vivo_data[i,'Stages'] ==
'MO_BL'){


    if(mouse_vivo_data[i,'Direction'] == 'Down'){
      mouse_vivo_data$Prop[i] <- mouse_vivo_data[i,'PropDown']} else {
        mouse_vivo_data$Prop[i] <- -mouse_vivo_data[i,'PropUp']
      }
  }
}

# mean methylation values
mouse_vivo_methylation <- aggregate(meth_scaled ~ Stage, data =
mouse_vivo_methylation, FUN = 'mean')
mouse_vivo_methylation$Stage <- factor(mouse_vivo_methylation$Stage, ordered
= TRUE, levels = c("MII", "MII_2C", "2C_MII", "2C", "2C_4C", "4C_2C",
"4C","4C_8C", "8C_4C", "8C",

"8C_16C", "16C_8C", "16C", "16C_BL", "BL_16C", "MO", "MO_BL", "BL_MO", "BL"))


#Correlations between methylation and pathway up/down regulation
PATHWAYS <- unique(mouse_vivo_data$Pathway)
COR <- list()
for(i in 1:length(PATHWAYS)){
  PATH <- mouse_vivo_data[which(mouse_vivo_data$Pathway == PATHWAYS[i]),
c('Stages', 'Prop')]
  MERGE <- merge(PATH, mouse_vivo_methylation, by.x = 'Stages', by.y =
'Stage')
  COR[[i]] <- data.frame(Pathway = PATHWAYS[i],
                         rho = cor(MERGE$Prop, MERGE$meth_scaled),
                         source = 'mouse_vivo')
}
COR_mouse_vivo <- do.call(rbind, COR)

# Stage means across all the genes
ALL_MEANS <- aggregate(Prop ~ Stages, data =mouse_vivo_data, FUN = "mean")
ALL_MEANS$Prop_Scaled <- scale(ALL_MEANS$Prop, center = TRUE, scale = TRUE)
```

```
############################################
# Humans
############################################

# Human Vitro

#Data wrangling
human_vitro_data$Prop <- NA
for(i in 1:nrow(human_vitro_data)){
  #Which direction
  if(human_vitro_data[i,'Direction'] == 'Down'){
    human_vitro_data$Prop[i] <- -human_vitro_data[i,'PropDown']} else {
      human_vitro_data$Prop[i] <- human_vitro_data[i,'PropUp']
    }

  #Which direction
  if(human_vitro_data[i,'Stages'] == '2C_MII' | human_vitro_data[i,'Stages']
== '4C_2C' |
     human_vitro_data[i,'Stages'] == '8C_4C' | human_vitro_data[i,'Stages']
== '16C_8C' |
     human_vitro_data[i,'Stages'] == "BL_16C"| human_vitro_data[i,'Stages']
== 'MO_BL'){

    if(human_vitro_data[i,'Direction'] == 'Down'){
      human_vitro_data$Prop[i] <- human_vitro_data[i,'PropDown']} else {
        human_vitro_data$Prop[i] <- -human_vitro_data[i,'PropUp']
      }
  }
}

# mean methylation values
human_vitro_methylation <- aggregate(meth_scaled ~ Stage, data =
human_vitro_methylation, FUN = 'mean')
human_vitro_methylation$Stage <- factor(human_vitro_methylation$Stage,
ordered = TRUE, levels = c("MII", "MII_2C", "2C_MII", "2C", "2C_4C", "4C_2C",
"4C","4C_8C", "8C_4C", "8C", "8C_16C", "16C_8C", "16C", "16C_BL", "BL_16C",
"MO", "MO_BL", "BL_MO", "BL"))


#Correlations between methylation and pathway up/down regulation
PATHWAYS <- unique(human_vitro_data$Pathway)
COR <- list()
for(i in 1:length(PATHWAYS)){
  PATH <- human_vitro_data[which(human_vitro_data$Pathway == PATHWAYS[i]),
c('Stages', 'Prop')]
  MERGE <- merge(PATH, human_vitro_methylation, by.x = 'Stages', by.y =
'Stage')
```

```
  COR[[i]] <- data.frame(Pathway = PATHWAYS[i],
                         rho = cor(MERGE$Prop, MERGE$meth_scaled),
                         source = 'human_vitro')
}
COR_human_vitro <- do.call(rbind, COR)


# Stage means across all the genes
ALL_MEANS <- aggregate(Prop ~ Stages, data =human_vitro_data, FUN = "mean")
ALL_MEANS$Prop_Scaled <- scale(ALL_MEANS$Prop, center = TRUE, scale = TRUE)


#Data for correlation heatmap
correlations <- do.call(rbind, list(COR_cow_vivo, COR_cow_vitro,
COR_mouse_vivo,COR_mouse_vitro,COR_human_vitro))

correlations$source <- factor(correlations$source, ordered = TRUE, levels =
c("cow_vivo", "cow_vitro", "mouse_vivo", "mouse_vitro", "human_vitro"))

#Drop pathway with insufficient data
correlations <- correlations[-which(correlations$Pathway %in%
PATHWAYS[c(38,64,65,66,67,68,69,71,72,73,78,
                                           79,81,83,86:104,107,108)]),]

correlations$Pathway <- factor(correlations$Pathway, ordered = TRUE, levels =
COR_cow_vivo$Pathway)


correlations[is.na(correlations)] <- 0
LABELS <- c("Glycogen metabolism",
            "Gluconeogenesis",
            "Glycolysis",
            "Fuctose biosynthesis",
            "Fructose catabolism",
            "Lactose synthesis",
            "Galactose catabolism",
            "PPP",
            "Glycosaminoglycan metabolism",
            "Inositol phosphate metabolism",
            "Fatty AcylCoA byosynthesis",
            "Arachdonic acid metabolism",
            "O3 and O6 metabolism",
            "Carnitine metabolism",
            "Beta-oxidation",
            "Peroxisomal lipid metabolism",
            "Triglycerid biosynthesis",
            "Tryglicerid catabolism",
            "Phospholipid metabolism",
            "Sphingolipid metabolism",
```

```
            "Steroids metabolism",
            "Ketone body metabolism",
            "Nitric oxide metabolism",
            "Pyruvate metabolism",
            "TCA",
            "ETC",
            "Nucleotides metabolism",
            "Ascorbate metabolism",
            "Thiamine metabolism",
            "Riboflavin metabolism",
            "Panthotenate metabolsim",
            "Cobalamine metabolism",
            "Biotin metabolism",
            "Nicotinate metabolism",
            "Folate metabolism",
            "Vitamin K metabolism",
            "Retinoid metabolism",
            "Co-factors metabolism",
            "Aspartate and asparagine metabolism",
            "Glutamate and glutaminemetabolism",
            "Alanine metabolism",
            "Branched chain aa catabolism",
            "Histidine catabolism",
            "Lysine catabolism",
            "Phenylalanine metabolism",
            "Tyrosine catabolism",
            "Proline catabolism",
            "Serine biosynthesis",
            "Threonine catabolism",
            "Tryptophan catabolism",
            "Methionine salvage pathway",
            "Sulfur aa metabolism",
            "Selenoamino acid metabolism",
            "Glyoxylate metabolism",
            "Urea cycle",
            "Carnitine synthesis",
            "Creatine metabolism",
            "Choline catabolism",
            "Polyamines metabolism",
            "Melanin byosinthesis",
            "Amine derived hormones metabolism",
            "Mitochondria iron sulfur biogenesis",
            "Mitochondrial translation",
            "Asparagine N linked glycosylation",
            "O linked glycosylation",
            "SUMOylation",
            "Deubiquitination",
            "Protein methylation",
            "Neddylation",
            "Peptide hormone metabolism",
```

```
             "IGF transport and uptake by IGFBPs",
             "PRC2 methylates histones and DNA",
             "TET1, 2, 3 and TDG demethylate DNA",
             "DNA methylation",
             "HATs acetylate histones",
             "HDACs deacetylate histones",
             "HDMs demethylate histones",
             "PKMTs methylate histone lysines",
             "RMTs methylate histone arginines",
             "Cellular response to hypoxia",
             "Detoxification of Reactive Oxygen Species",
             "Cristae formation")

#Plot the results
ggplot() +
  geom_tile(data = correlations, aes(x = source, y = Pathway, fill = rho),
            color = "black", size = 0.01) +
  scale_fill_gradient2(low = "#008282", high = "#c53f27",
                       midpoint = 0, mid = "white",
                       na.value = "black", name = expression(rho),
                       limits=c(-1,1)) +
  ylab('') +
  xlab("") +
  theme_bw() +
  theme(legend.title = element_text(size = 10, family = "serif"),
        legend.text = element_text(size = 10, family = "serif"),
        plot.title = element_text(size=16, family = "serif"),
        axis.title = element_text(size=12, family = "serif"),
        axis.text.y = element_text(size=8, family = "serif"),
        axis.text.x = element_text(size=7, family = "serif")) +
  scale_x_discrete(expand = c(0,0.1), breaks = c("cow_vivo", "cow_vitro",
                                                  "mouse_vivo", "mouse_vitro",
                                                  "human_vitro"),
                   labels = c("Bovine in vivo", "Bovine in vitro",
                              "Mouse in vivo", "Mouse in vitro",
                              "Human in vitro")) +
  scale_y_discrete(expand = c(0,0),
                   labels = LABELS)
```

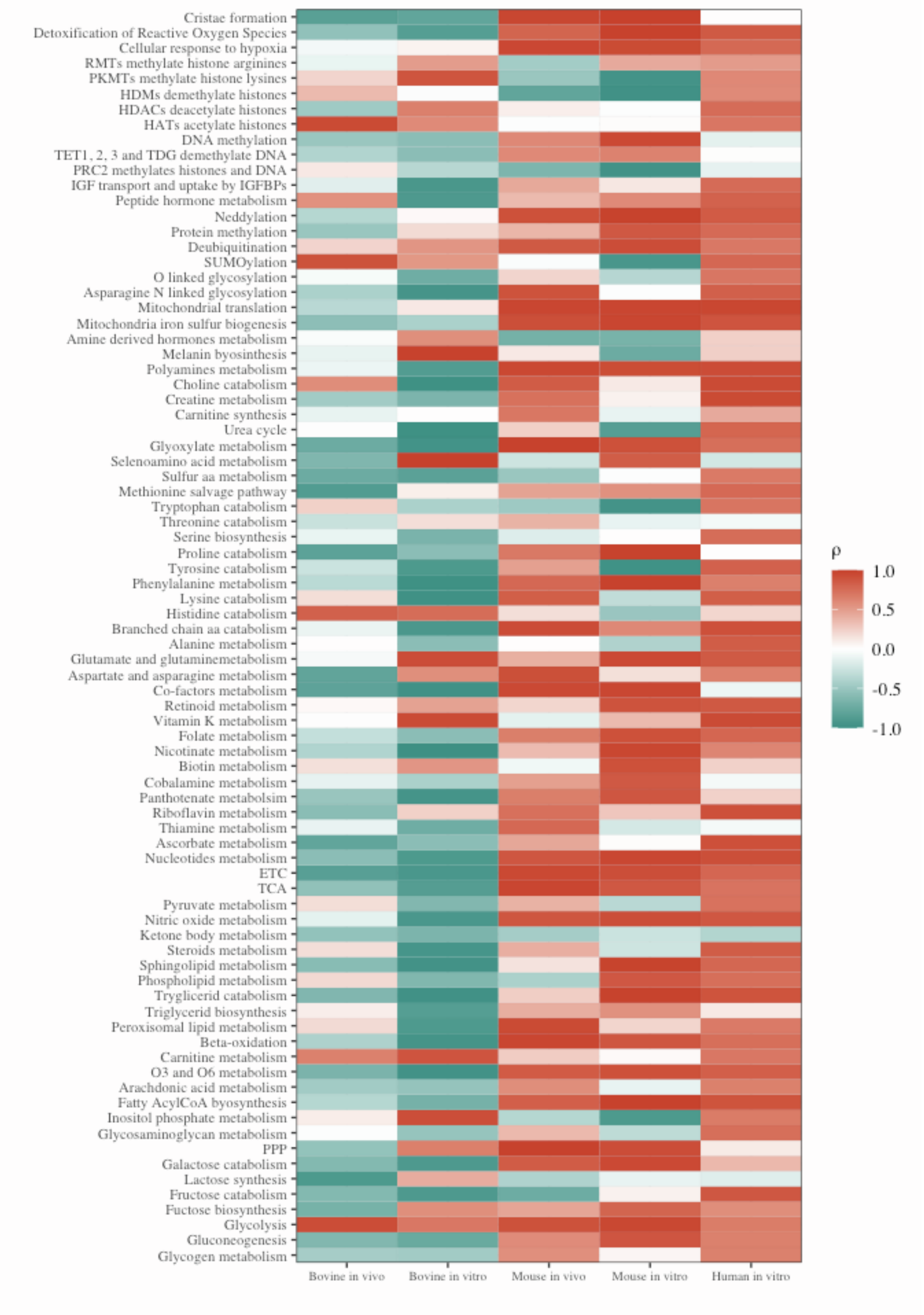

Cristae formation
Detoxification of Reactive Oxygen Species
Cellular response to hypoxia
RMTs methylate histone arginines
PKMTs methylate histone lysines
HDMs demethylate histones
HDACs deacetylate histones
HATs acetylate histones
DNA methylation
TET1, 2, 3 and TDG demethylate DNA
PRC2 methylates histones and DNA
IGF transport and uptake by IGFBPs
Peptide hormone metabolism
Neddylation
Protein methylation
Deubiquitination
SUMOylation
O linked glycosylation
Asparagine N linked glycosylation
Mitochondrial translation
Mitochondria iron sulfur biogenesis
Amine derived hormones metabolism
Melanin byosinthesis
Polyamines metabolism
Choline catabolism
Creatine metabolism
Carnitine synthesis
Urea cycle
Glyoxylate metabolism
Selenoamino acid metabolism
Sulfur aa metabolism
Methionine salvage pathway
Tryptophan catabolism
Threonine catabolism
Serine biosynthesis
Proline catabolism
Tyrosine catabolism
Phenylalanine metabolism
Lysine catabolism
Histidine catabolism
Branched chain aa catabolism
Alanine metabolism
Glutamate and glutaminemetabolism
Aspartate and asparagine metabolism
Co-factors metabolism
Retinoid metabolism
Vitamin K metabolism
Folate metabolism
Nicotinate metabolism
Biotin metabolism
Cobalamine metabolism
Panthotenate metabolsim
Riboflavin metabolism
Thiamine metabolism
Ascorbate metabolism
Nucleotides metabolism
ETC
TCA
Pyruvate metabolism
Nitric oxide metabolism
Ketone body metabolism
Steroids metabolism
Sphingolipid metabolism
Phospholipid metabolism
Tryglicerid catabolism
Triglycerid biosynthesis
Peroxisomal lipid metabolism
Beta-oxidation
Carnitine metabolism
O3 and O6 metabolism
Arachdonic acid metabolism
Fatty AcylCoA byosynthesis
Inositol phosphate metabolism
Glycosaminoglycan metabolism
PPP
Galactose catabolism
Lactose synthesis
Fructose catabolism
Fuctose biosynthesis
Glycolysis
Gluconeogenesis
Glycogen metabolism
Bovine in vivo
Bovine in vitro
Mouse in vivo
Mouse in vitro
Human in vitro
ρ
1.0
0.5
0.0
-0.5
-1.0

0.9 Session Info

Detail of the `R` session info for reproducibility.

```
sessionInfo()
```

```
## R version 4.1.2 (2021-11-01)
## Platform: x86_64-apple-darwin17.0 (64-bit)
## Running under: macOS Big Sur 10.16
##
## Matrix products: default
## BLAS:
/Library/Frameworks/R.framework/Versions/4.1/Resources/lib/libRblas.0.dylib
## LAPACK:
/Library/Frameworks/R.framework/Versions/4.1/Resources/lib/libRlapack.dylib
##
## locale:
## [1] en_US.UTF-8/en_US.UTF-8/en_US.UTF-8/C/en_US.UTF-8/en_US.UTF-8
##
## attached base packages:
## [1] stats     graphics  grDevices utils     datasets  methods   base
##
## other attached packages:
##  [1] limma_3.50.0        gridExtra_2.3       viridis_0.6.2
##  [4] viridisLite_0.4.0   ggridges_0.5.3      caret_6.0-90
##  [7] lattice_0.20-45     randomForest_4.6-14 ellipse_0.4.2
## [10] ggplot2_3.3.5       FactoMineR_2.4
##
## loaded via a namespace (and not attached):
##  [1] splines_4.1.2        foreach_1.5.1        prodlim_2019.11.13
##  [4] highr_0.9            stats4_4.1.2         yaml_2.2.1
##  [7] ggrepel_0.9.1        globals_0.14.0       ipred_0.9-12
## [10] pillar_1.6.4         glue_1.6.0           pROC_1.18.0
## [13] digest_0.6.29        colorspace_2.0-2     recipes_0.1.17
## [16] htmltools_0.5.2      Matrix_1.4-0         plyr_1.8.6
## [19] timeDate_3043.102    pkgconfig_2.0.3      listenv_0.8.0
## [22] bookdown_0.24        purrr_0.3.4          scales_1.1.1
## [25] gower_0.2.2          lava_1.6.10          tibble_3.1.6
## [28] farver_2.1.0         generics_0.1.1       ellipsis_0.3.2
## [31] DT_0.20              withr_2.4.3          nnet_7.3-16
## [34] survival_3.2-13      magrittr_2.0.1       crayon_1.4.2
## [37] evaluate_0.14        future_1.23.0        fansi_0.5.0
## [40] parallelly_1.30.0    nlme_3.1-153         MASS_7.3-54
## [43] class_7.3-19         tools_4.1.2          data.table_1.14.2
## [46] lifecycle_1.0.1      stringr_1.4.0        munsell_0.5.0
## [49] cluster_2.1.2        flashClust_1.01-2    compiler_4.1.2
## [52] rlang_0.4.12         grid_4.1.2           iterators_1.0.13
## [55] htmlwidgets_1.5.4    leaps_3.1            labeling_0.4.2
## [58] rmarkdown_2.11       gtable_0.3.0         ModelMetrics_1.2.2.2
## [61] codetools_0.2-18     reshape2_1.4.4       R6_2.5.1
```

```
## [64] lubridate_1.8.0      knitr_1.37           dplyr_1.0.7
## [67] fastmap_1.1.0        future.apply_1.8.1   utf8_1.2.2
## [70] stringi_1.7.6        parallel_4.1.2       Rcpp_1.0.7
## [73] vctrs_0.3.8          rpart_4.1-15         scatterplot3d_0.3-41
## [76] tidyselect_1.1.1     xfun_0.29
```
